# Phylogeny and species diversity of *Armillaria* in China based on morphological, mating test, and GCPSR criteria

**DOI:** 10.1080/21501203.2024.2404121

**Published:** 2024-11-13

**Authors:** Guo-Fu Qin, Wen-Min Qin, Han-Chen Wang, Jun Zhao, Kari Korhonen, Jian Chen, Yu-Cheng Dai, Yuan Yuan

**Affiliations:** a State Key Laboratory of Efficient Production of Forest Resources, School of Ecology and Nature Conservation, Beijing Forestry University, Beijing, China; b Center for Biological Disaster Prevention and Control, National Forestry and Grassland Administration, Shenyang, China; c Institute of Applied Ecology, Chinese Academy of Sciences, Shenyang, China; d College of Life Science, Chongqing Normal University, Chongqing, China; e Natural Resources Institute Finland (Luke), Kirkkonummi, Finland

**Keywords:** Agaricales, biological species, phylogenetic species, new species description, GCPSR

## Abstract

More than 600 Chinese specimens of *Armillaria* were identified by mating tests, Genealogical Concordance Phylogenetic Species Recognition (GCPSR), and comparison of morphological characteristics. Sixteen Chinese Biological Species (CBS) of *Armillaria* were identified by 30,340 mate pair combinations. Fifteen Chinese Phylogenetic Species (CPS) were recognised based on Independent Evolutionary Lineage (IEL) recognition and concatenated six-gene analysis (*actin*, *h3h*, *hisps*, LSU rDNA, *rpb*1, and *tef*1α). All the biological species and phylogenetic species were identical and possessed the same species boundary, except for CBS K (*A. mellea*) and CBS G (*A. mellea* ssp. *nipponica*) which were the same phylogenetic species. On the basis of CBS and CPS, eight new species of *Armillaria* in China were distinguished using macro and micro morphology, and they are described as *A. algida*, *A. amygdalispora*, *A. bruneocystidia*, *A. luteopileata*, *A. pungentisquamosa*, *A. sinensis*, *A. tibetica*, and *A. violacea*. This study indicates that the GCPSR approach provides the same resolution as mating tests in identification of *Armillaria* species.

## 1. Introduction

The type species of the genus *Armillaria* (Fr.) Staude was described by the Danish botanist Martin Vahl in 1766 (Watling et al. 1982, 1991; Pegler 2000). Since then, 281 names have been included in the genus but only 41–47 species have been accepted by researchers (Watling et al. 1991; Volk and Burdsall 1995; Pegler 2000; Lima et al. 2008; Pildain et al. 2010; Brazee et al. 2012; Hood and Ramsfield 2016; Elias-Roman et al. 2018). During the last two centuries, a large number of species have been distinguished based mainly on morphology, and transferred in and out of the genus depending on its delimitation and typification (Watling et al. 1982; Volk and Burdsall 1995), until the intersterility groups (ISGs) or biological species of *Armillaria* were identified (Korhonen 1978; Ullrich and Anderson 1978). The biological species concept of *Armillaria* was soon accepted by both systematic mycologists and plant pathologists, because the gene pool of an intersterility group allows convergent evolution of all genetic traits, including physiological and morphological characteristics, whereas genetic barriers between intersterility groups lead to divergent evolution (Korhonen 1978; Anderson and Ullrich 1979; Guillaumin et al. 1991). A series of new taxonomic species of *Armillaria* have been either described or classical phenotypic species re-identified by systematic mycologists around the world. Ten species have been reported to occur in North America (Bérubé and Dessureault 1988, 1989; Darmono et al. 1992; Volk et al. 1996; Brazee et al. 2012), seven in Europe (Gregory and Watling 1985; Watling 1987, 1992; Termorshuizen and Arnolds 1987, 1997; Marxmüller 1992), eight in Australia and New Zealand (Kile and Watling 1983, 1988; Pegler 2000; Coetzee et al. 2001; Hood and Ramsfield 2016), and twelve in Japan (Cha et al. 1994; Cha and Igarashi 1995; Ota et al. 1998, 2009, 2011; Kudo and Nagasawa 2003; Hasegawa et al. 2010). In the past four decades, all of the above studies have demonstrated that the identification of biological species is a vital prerequisite for distinguishing species of *Armillaria*, and the taxonomy of *Armillaria* based on biological species is quite reliable and effective, especially for the heterothallic species.

On the other hand, DNA-based diagnostic tools play an increasingly important role in distinguishing species and understanding phylogenetic relationships among *Armillaria* species. Initially, non-coding IGS1 and ITS sequences were used in analysing phylogenetic relationships of *Armillaria* species (Anderson and Stasovski 1992; Chillali et al. 1998; Terashima et al. 1998). Although rDNA (IGS, ITS, and nLSU) sequences cannot discriminate the species of the “Gallica cluster” in North America (Kim et al. 2006), IGS1-RFLP and ITS-RFLP can successfully distinguish most species of *Armillaria* in Europe and North America, and IGS1-RFLP typing technique has become an important molecular tool for distinguishing Euramerican species of *Armillaria* (Harrington and Wingfield 1995; Terashima et al. 1998; Pérez-Sierra et al. 1999; Kim et al. 2000; Tsykun et al. 2013). Recently, the coding-gene sequences of the actin gene (*actin*), translation elongation factor subunit 1-alpha gene (*tef*1α), glyceraldehyde 3-phosphate dehydrogenase gene (*gpd*), RNA polymerase subunit II gene (*rpb*2), ATP synthase subunit 6 gene (*atp*6), beta-tubulin, and 28S rDNA have been used to distinguish *Armillaria* species and analyse their phylogenetic relationships (Hasegawa et al. 2010; Brazee et al. 2011; Baumgartner et al. 2012; Ross-Davis et al. 2012; Guo et al. 2016; Klopfenstein et al. 2017; Koch et al. 2017). Among these genes, *tef*1α sequence had a better resolution than the other loci (Hasegawa et al. 2010; Brazee et al. 2011, 2012; Baumgartner et al. 2012; Mulholland et al. 2012; Ross-Davis et al. 2012; Tsykun et al. 2013; Klopfenstein et al. 2017). Other molecular polymorphic tools, e.g. amplified fragment length polymorphisms (AFLP) and random amplified microsatellites (RAMS) have been quite useful for understanding phylogenetic relationships among *Armillaria* species (Qin et al. 2001; Qin and Hantula 2002; Kim et al. 2006; Terashima et al. 2006). The genomes of three European species (*A. ostoyae*, *A. cepistipes*, and *A. gallica*) and one North American taxon (*A*. *solidipes*) were sequenced, and a phylogenetic tree was reconstructed with 188,895 amino acid sites of 835 conserved single-copy genes (Sipos et al. 2017). In addition, annotated genome drafts of *A. mellea* (Collins et al. 2013), *A. fuscipes* (Wingfield et al. 2016), *A. borealis*, *A. ectypa*, *A. fumosa*, *A. luteobubalina*, *A. nabsnona*, *A. novae-zealandiae*, *A. ostoyae*, *A. solidipes*, and *Desarmillaria tabescens* have been released and are available online (https://genome.jgi.doe.gov/).

The concept of evolutionary species was proposed by Simpson (1951), and although it was not widely adopted due to lack of recognition criterion it laid the foundation for the follow-up phylogenetic species concept (PSC) (Taylor et al. 2000). Then, various PSCs were proposed and, in general, could be classified into two types: character-based and lineage-based species concepts (Baum and Donoghue 1995; Balakrishnan 2005), but both have shortages in implementation. For example, the former greatly increased the number of species, and the latter was difficult to give monophyly and exclusivity consideration simultaneously (Baum 1992; Baum and Donoghue 1995). On the basis of PSC, a concept of multilocus genealogical concordance, reconciling multilocus PSC and BSC, was proposed by Avise (1990), Avise and Wollenberg (1997), and Graybeal (1995). Then, Taylor et al. (2000) applied this concept to fungi and modified it as Genealogical Concordance Phylogenetic Species Recognition (GCPSR). The basic idea of GCPSR was to look for a boundary of biological species by comparison of multi-gene phylogeny, that is, between genetically isolated species, the extinction of ancestral alleles by genetic drift would lead to the congruence of genealogies. In contrast, within a single interbreeding species, the mixing effects of recombination caused unlinked loci to have incongruent genealogies. Therefore, the transition between deep genealogical concordance and shallow genealogical discordance was used to recognise phylogenetic species (Taylor et al. 2000; Dettman et al. 2003a). The purpose was to provide concordance of at least four unlinked nuclear loci. However, it is necessary to pay special attention to the fact that it is not possible to recognise phylogenetic species based on a single or a few genes. In the light of coalescent theory, a clade based on one or a few genes makes less sense regardless of any approach (Avise and Wollenberg 1997; Grube and Kroken 2000; Taylor et al. 2000). The GCPSR is currently more widely implemented in the field of fungal systematics, especially in plant pathogens that are clonal lineages (Laurence et al. 2014), or difficult to cultivate (Vialle et al. 2013), or belong to secondary homothallic species complex (Menkis et al. 2009). Up to now, however, GCPSR has not yet been applied to the taxonomy of *Armillaria* due to the absence of unlinked single-copy genes that can identify Independent Evolutionary Lineage (IEL) (Coetzee et al. 2018).

In China, modern systematics of *Armillaria* on the basis of biological species started 28 years ago (He et al. 1996). Fourteen Chinese Biological Species (CBS) of *Armillaria* were recognised by 2004 (Qin et al. 2007). A preliminary phylogeny on 35 strains of the nine CBS was established based on the sequence of Intergenic Space1 (IGS1) (Qin 2002). Recently, Coetzee et al. (2015), Guo et al. (2016), and Liang et al. (2021) used the *tef*1α gene sequence to perform phylogenetic analysis on limited isolates and species. Peng and Zhao (2020) described a new species of *Armillaria* based only on the *tef*1 sequence, but the new species lacked intersterility information. Liu et al. (2024) have published *Armillaria korhonenii*, a new taxonomic species from Yunnan Province in China. So far, most CBS have not been described as taxonomic species. The main objective of this study was to identify biological species of *Armillaria* by means of the mating tests, to design a series of primers of the protein-coding gene and perform a GCPSR, and to formally describe new species in China.

## 2. Materials and methods

### 2.1. Collection and morphological studies

Since 1996, more than 600 *Armillaria* specimens have been collected from 26 provinces in China. Each specimen was photographed *in situ* and macromorphological characteristics were noted, with notes on the substrate and other ecological information such as surrounding vegetation type. Colour codes followed by Kornerup (1984). Spore prints were made on sterilised filter paper and carried to the laboratory in a cooled thermos bottle for single spore isolation. Fresh basidiomata were dried in a mushroom dryer at 40 °C and used for morphological and microscopic examination. The voucher specimens, holotypes, and isotypes were stored at the herbarium of the Institute of Applied Ecology, Chinese Academy of Science (IFP). The information on the specimens used in the microscopic and molecular phylogenetic studies is listed in Table S1.

A total of 256 basidiomata were used in the micromorphology studies (Table S1). Dried specimens were rehydrated in 2%–5% KOH and mounted in different mountants, viz., 1% Congo Red for photographing, 0.0006 mol/L Cotton Blue for measuring, and Melzer’s reagents for any amyloid or dextrinoid reaction. At least 30 basidiospores were measured from a specimen in Cotton Blue, and the measurements were used to calculate the proportion of spore length to width (Q value); the shape of the spores was described according to Bas (1969): globose Q = 1.0–1.05; subglobose Q = 1.05–1.15; broadly ellipsoid Q = 1.15–1.3; ellipsoid Q = 1.3–1.6; elongate Q = 1.6–1.8; cylindrical Q = 2.0–3.0; bacilliform Q > 3.0. To represent variation in the size of spores, 10% of measurements were excluded from each end of the range and are given in parentheses. Q refers to the range of the length-breadth ratio of the spores, *Q*_*m*_ ± SD refers to the mean and standard deviation of the Q value whereas *Q*_*m*_ ± EL refers to the mean and error limits of the Q value. Similarly, *L*_*m*_ ± SD refers to the mean and standard deviation of the spore length whereas *L*_*m*_ ± EL refers to the mean and error limits of the spore length.

### 2.2. Single spore isolation and identification of intersterility groups

See Qin et al. (2007). The L-DOPA method was used for the judgement of cross results between secondary homothallic and heterothallic isolates (Hopkin et al. 1989; Mallett et al. 1989). North American isolates of *Armillaria* were kindly provided by Prof. T.C. Harrington (Iowa State University, USA), Dr. D.J. Morrison (Pacific Forestry Centre, Canada), Dr. J. Mclaughlin and Dr. G.H. Hayden (Ontario Forest Research Institute, Canada). African isolates were obtained from Dr. J.J. Guillaumin (INRA, Centre de Clermont-Theix, France). The isolates from Belarus and Greece were provided by Dr. V. Zviahintsau and Dr. P. Tsopelas, respectively. The species of the above *Armillaria* haploid isolates were identified by mating tests.

### 2.3. DNA extraction and PCR amplification

For DNA extraction, haploid isolates were grown on cellophane membranes placed on a medium that was composed of 2% malt extract, 2% glucose, and 0.5% peptone. Harvesting was done after incubation at 25 °C for 3 weeks. DNA was extracted according to Liu et al. (1997) and Vainio et al. (1998).

The primers Fact1421 and Ract2258 were re-designed for amplifying the actin gene (*actin*), because some Chinese isolates of *Desarmillaria tabescens* were difficult to amplify using ArmActF and ArmActR primers (Baumgartner et al. 2012). According to the sequences published by Kotlobay et al. (2018), two forward primers, H3hf1 and H3hf3, and two reverse primers H3hr1 and H3hr3, were designed for amplifying the hispidin-3-hydroxylase gene (*h3h*), and two forward primers, HPf1655 and HPf1676, and a reverse primer HPr3131, were designed for amplifying the hispidin synthase gene (*hisps*). The partial sequences of the large subunit of the rRNA gene (LSU rDNA) were amplified by primers ArmLRoR and ArmLR7, which were modified from Vilgalys and Hester (1990) and Moncalvo et al. (2000), respectively. Two primer pairs of the largest subunit of RNA polymerase II gene (*rpb*1), AF7786/R9448 and Rpbf1/Rpbr1 were designed based on the GenBank LR732075 sequence of *A. ostoyae*. For partial translation elongation factor subunit 1-alpha gene (*tef*1α), forward primer EF595F (Maphosa et al. 2006) and a reverse primer QtefR were used for amplifying a longer fragment. The QtefR was designed according to 36 sequences of *Armillaria* and *Guyanagaster* in GenBank (Ross-Davis et al. 2012; Koch et al. 2017). All primer sequences and annealing temperatures are listed in Table 1.

**Table 1.** Primer sequences and annealing temperatures used in this study.

Locus	Primers	Primer sequences (5'→3')	Annealing temperature (°C)	Reference
*actin*	Fact1421	GAT GAA GCA CAA TCT AAG CGA GGT	60	This study
	Ract2258	TCA CGA CGG ATA TCA AGA TC	60	This study
*h3h*	H3hf1	ATG CAA CAA ATC GAC GAA G	60	This study
	H3hr1	CTG AGA TAC AAG CCT CGC TTT	60	This study
	H3hf3	TAG ATA CTT GAA TCT TCA A	49	This study
	H3hr3	AGA CCC ATC CCG AAG CCT TG	60	This study
*hisps*	HPf1655	CGA GGA AAG GCG ATG TGT T	58–60	This study
	HPf1676	TCC GTA AGA AGA TTG AAG AT	50	This study
	HPr3131	CCC TTG AGA ACG GCC GTT ATG	58–60	This study
LSU rDNA	ArmLRoR	ACC CGC TGA ACT TAA GCA TAT C	58–60	This study
	ArmLR7	GCT ACT ACC ACC AAG ATC TGC	58–60	This study
*rpb*1	AF7786	CCC AAT TTT CTG GGG GCT CTC	58	This study
	R9448	CAC AAA ATG AGT ATG ATG AGT C	58	This study
	Rpbf1	GCG TTT TCG GTC GCT TGA TCG CCG	60–72	This study
	Rpbr1	TAC GAA CCA GTT CCT GCA GGT AAG C	60–72	This study
*tef*1α	EF595F	CGT GAC TTC ATC AAG AAC ATG	60	Maphosa et al. (2006)
	QtefR	GAT TTA CCC GTT CGG CGA TCA AT	60	This study

Amplification was carried out in a 30 μL reaction mixture using Phire Hot Start II DNA Polymerase and Thermo Scientific Phire Plant Direct PCR Kit according to the manufacturer’s instructions. The PCR conditions were as follows: 98 °C for 30 s, 36 cycles of 98 °C for 6 s, 50 °C to 62 °C (depending on the primers) for 6 s, and 72 °C for 15 s per kb; then 72 °C for 5 min. For primer Rpbf1/Rpbr1, a two-step PCR was carried out: 98 °C for 30 s, 36 cycles of 98 °C for 6 s, and 72 °C for 25 s, then 72 °C for 5 min.

### 2.4. Sequence alignment and phylogenetic analysis

We chose the Muscle program as the multiple sequence alignment (MSA) method because Muscle achieved a better resolution than ClustalW, MAFFT, and PRANK for the 111 *tef*1α sequences from GenBank in our pre-analysis. Muscle alignments were carried out on the MEGA version X platform with refinements to the alignment done manually. For evolutionary analysis algorithms, Maximum Parsimony (MP) and MrBayes (MB) were used for the recognition of independent evolutionary lineage (IEL) according to Dettman et al. (2003a) and Dettman et al. (2003b). The best-fit substitution model of each combination was selected by the lowest BIC scores (Bayesian Information Criterion) on MEGA version X according to cAIC value (Corrected Akaike Information Criterion) and the number of parameters including branch lengths (Nei and Kumar 2000; Kumar et al. 2018). MP evolutionary analysis method followed Felsenstein (1985). The MP tree was obtained using the Subtree-Pruning-Regrafting (SPR) algorithm (Nei and Kumar 2000). For MB analysis, DataConvert and jEdit 5.5 were used to create an input file. MB analysis was conducted with MrBayes 3.2.6 (Ronquist et al. 2012) using four simultaneous, independent Markov Chain Monte Carlo (MCMC), and two current runs of 30 million generations (150 million generations for a six-locus concatenated phylogeny). Trees were sampled every 100 generations and the first 25% were discarded as burn-in. Scatterplots were generated to determine stationarity (*n* = 1,000). The remaining posterior probability distributions were used to compute a 50% majority-rule tree. For concatenated six-gene dataset analysis, the RAxML version 8 (Stamatakis 2014) was used to reconstruct a Maximum Likelihood (ML) PS tree and calculate the MLBS (Maximum Likelihood Bootstrap Support) of each PS. Tree files were visualised in FigTree v.1.4.4 (http://tree.bio.ed.ac.uk/software.figtree/). *Desarmillaria ectypa* was used as an outgroup because the species does not occur in China and is at the most basic position on the phylogenetic tree of *Armillaria* and *Desarmillaria* (Koch et al. 2017; Coetzee et al. 2018).

### 2.5. Phylogenetic species recognition by genealogical concordance

MP and MB analyses were carried out for each gene dataset. Phylogenetic species were recognised using grouping and ranking methods defined by (Dettman et al. 2003a, 2003b). A clade was recognised as an IEL if it satisfied either of two grouping criteria: (1) Genealogical concordance, i.e. the clade was present in the majority (3/4) of the single-locus genealogies. To identify such clades, a majority-rule consensus tree was produced from at least four single-locus trees, regardless of levels of support. (2) Genealogical nondiscordance, i.e. the clade was well supported by both Maximum Parsimony Bootstrap Proportion (MPBP) and Bayesian Posterior Probabilities (BPP) in at least one single-locus genealogy, and was not contradicted in any other single-locus genealogy at the same level of support. However, because both LSU rDNA and nuclear protein-coding genes (*rpb*1, *tef*1α, *actin*, *hisps*, and *h3h*) used in the study were considered overly conservative, a clade was considered to be genealogical concordant and thus an IEL if it were found in at least one single-gene majority-rule consensus tree of maximum parsimony and Bayesian analyses and the clade was required to have both MPBP and BPP support value ≥ 70% and ≥ 95%, respectively. After IEL recognition for each gene dataset, all sequences from the above six genes were concatenated using Mesquite (Maddison and Maddison 2023, v.3.81); this concatenated data matrix was calculated using MEGA, RAxML, and MrBayes. Then, a Bayesian multigenic species tree with the MPBP, MLBS, and BPP support values was generated. The second step included two ranking criteria: (1) Genetic differentiation required that a clade is well differentiated from other species to prevent minor tip clades from being recognised as phylogenetic species. (2) Exhaustive subdivision required that all individuals had to be placed into a phylogenetic species so no individuals remain unplaced (Dettman et al. 2003a, 2003b; Menkis et al. 2009; Laurence et al. 2014). The MrBayes multigenic species tree and IELs on single-gene trees were used simultaneously to distinguish phylogenetic species. In this study, two ranking criteria were used in the identification of phylogenetic species on the MrBayes species tree.

The structural annotation and translation of the coding sequence were completed by the Augustus software (Bioinf.uni-greifswald.de/bioinf/) and EMBL-EBI’s cloud services (Madeira et al. 2022), respectively. All sequences are deposited in GenBank and the accession numbers are listed in Table S1. The concatenated sequence matrix and phylogenetic trees are stored in TreeBASE: http://purl.org/phylo/treebase/phylows/study/TB2:S31129.

## 3. Results

### 3.1. Phylogenetic species recognition based on independent evolutionary lineage (IEL) analysis

We designed *de novo* amplification primers for *actin*, *h3h*, *hisps*, and *rpb*1. The primer pair Fact1421/Ract2258 acquired higher amplification efficiency in the amplification of *actin*. The primer pair H3hf1/H3hr1 obtained satisfactory amplification of the *h3h* gene of most species of *Armillaria*, and the primer pair H3hf3/h3hr3 was used in the amplification of *Desarmillaria tabescens* and *A. korhonenii*. For the amplification of *hisps*, usually the primer pair HPf1655/HPr3131 was used, however, the primer pair HPf1676/HPr3131 was alternatively used when products could not be obtained from isolates of *A. mellea*. For the amplification of *rpb*1, both the primers Rpbf1/Rpbr1 and AF7786/R9448 always produced good amplification, but sometimes the sequence resulting from AF7786/R9448 was too long (1,650 bp) to splice. Using the primers listed in Table 2, we sequenced a part of each of *actin*, *h3h*, *hisps*, LSU rDNA, *rpb*1, and *tef*1α for approximately 500 Chinese isolates or specimens. These sequences covered all geographical distribution regions and genetic diversity of each CBS. The product size of the six genes was approximately 810 bp, 1,450 bp, 1,550 bp, 1,350 bp, 1,300 bp, and 1,100 bp. After the ends of the individual alignments were trimmed, the size of the aligned datasets was as follows: 755 bp for *actin*; 1,288 bp for *h3h*; 1,451 bp for *hisps*; 1,338 bp for LSU rDNA; 1,160 bp *rpb*1; 893 bp for *tef*1α. The six-gene dataset was composed of 192 *Armillaria* specimens, representing 16 known CBS, and the European species *A. ectypa* was chosen as the outgroup (Table 2).

**Table 2.** The IEL recognised by single coding gene and the phylogenetic species recognised by concatenated six-gene.

Name of gene	*actin*	*h3h*	*hisps*	LSU rDNA	*rpb1*	*tef*1α	Concatenated six-gene
Number of sequences	238	211	232	228	223	285	212
Full sequence length	−810 bp	−1,450 bp	−1,550 bp	−1,350 bp	−1,300 bp	−1,100 bp	
Aligned length	755 bp	1,283 bp	1,452 bp	1,338 bp	1,160 bp	893 bp	6,880 bp
Intron	2	6	7	0	3	3	
Substitution model	K2+G; gamma	K2+G; gamma	T92+G; gamma	K2+G+I; invgamma	K2+G+I; invgamma	K2+G; gamma	K2+G+I; invgamma
IEL resolution	12/15	7/15	13/15	2/15	11/15	9/15	PS
*Armillaria algida*	IEL (96/100)				IEL (90/100)	IEL (95/100)	PS (94/100/100)
*A. amygdalispora*	IEL (71/100)		IEL (99/100)		IEL (33/100)	IEL (76/100)	PS (99/100/100)
*A. borealis*	IEL (98/100)		IEL (95/100)		IEL (73/100)		PS (51/30/95)
*A. bruneocystidia*							PS (89/100/100)
*A. gallica*	IEL (99/100)	IEL (97/100)	IEL (50/100)		IEL (99/100)		PS (99/100/100)
*A. korhonenii*	IEL (99/100)	IEL (99/100)	IEL (99/100)		IEL (100/100)	IEL (100/100)	PS (99/100/100)
*A. luteopileata*		IEL (91/100)	IEL (99/100)			IEL (77/100)	PS (99/100/100)
*A. mellea* (CBS G)	IEL (84/100)	IEL (99/100)	IEL (99/100)	IEL (99/100)	IEL (100/100)	IEL (100/100)	PS (99/100/100)
*A. ostoyae*	IEL (86/97)		IEL (93/100)			IEL (97/100)	PS (99/100/81)
*A. pungentisquamosa*	IEL (89/100)	IEL (99/100)	IEL (99/100)		IEL (100/100)	IEL (100/100)	PS (99/100/100)
*A. sinapina*	IEL (97/100)		IEL (93/100)		IEL (51/93)	IEL (100/100)	PS (99/100/100)
*A. sinensis*	IEL (90/100)		IEL (83/92)				PS (87/100/100)
*A. tibetica*			IEL (73/99)		IEL (89/100)		PS (95/100/100)
*A. violacea*	IEL (74/100)	IEL (92/100)	IEL (92/100)		IEL (63/100)		PS (99/100/100)
*Desarmillaria tabescens*	IEL (100/100)	IEL (99/100)	IEL (93/100)	IEL (90/96)	IEL (100/100)	IEL (100/100)	PS (99/100/100)

IEL: Independent evolutionary lineage. PS: Phylogenetic species. Two support values of IEL in brackets were MPBP/BPP. Three support values of PS in brackets were MPBP/MLBS/BPP.

Five protein-coding sequences (*actin*, *h3h*, *hisps*, *rpb*1, and *tef*1α), which are located on four unlinked nuclear loci, were subjected to IEL recognition by Maximum Parsimony (MP) and MrBayes (MB) analysis. CBS K (*Armillaria mellea*) and CBS G were recognised as the same IEL by all the protein-coding sequences (*actin*, *h3h*, *hisps*, *rpb*1, and *tef*1α) and LSU rDNA sequence, therefore, we count these two CBS as one phylogenetic species or species in the following text. Among 15 Chinese species of *Armillaria*, two species were recognised as IEL by LSU rDNA sequences, being the concatenated backbone in the multigenic dataset, seven species were recognised as IEL by *h3h* sequences (Figure S2, Table 2), nine species were recognised as IEL by *tef*1α sequences (Figure S6, Table 2), 11 species were recognised as IEL by *rpb*1 sequences (Figure S5, Table 2), 12 species were recognised as IEL by *actin* sequences (Figure S1, Table 2), 13 species were recognised as IEL by *hisps* sequences (Figure S3, Table 2). *A. bruneocystidia* was not recognised as IEL by any coding sequences (Figures S1–S6, Table 2). In brief, the resolution of recognising IEL was *hisps* > a*ctin* > *rpb*1 > *tef*1α > *h3h* (Figures S1–S6, Table 2). It is worth paying attention to the fact that only 55% of Chinese isolates of *Armillaria* could be differentiated as IEL or species by *tef*1α sequences.

### 3.2. *Phylogenetic species recognition based on concatenated six-gene* (actin, h3h, hisps, *LSU rDNA*, rpb*1*, *and* tef*1α)* *dataset*

**Figure 1.**
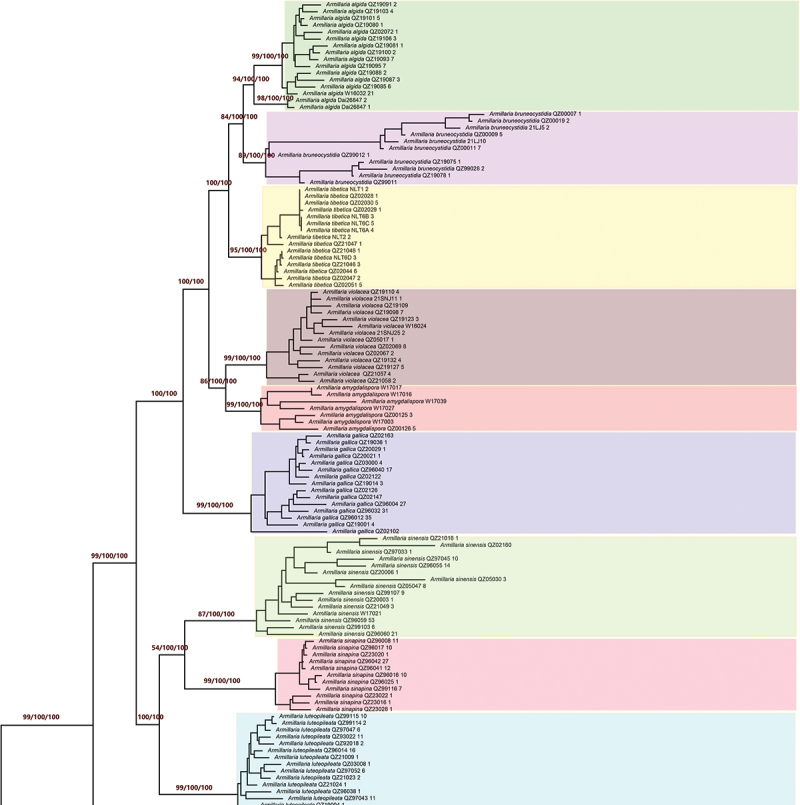
Phylogenetic species tree generated from concatenated *actin*, *h3h*, *hisps*, LSU rDNA, *rpb1*, and *tef*1α sequences. Statistical support refers to Maximum Parsimony Bootstrap Proportion (MPBP)/Maximum Likelihood Bootstrap Support (MLBS)/Bayesian Posterior Probability (BPP) values. If there are only two values then these are the supports of MLBS and BPP.

All six coding sequences were concatenated and a data matrix containing 192 sequences multiplying 6,880 bp was generated. The data matrix was analysed with MEGA, RaxML, and MrBayes programs and a Bayesian six-gene species tree with MPBP, MLBS, and BPP support values was finally produced. These 190 Chinese sequences of *Armillaria* were recognised as 15 Chinese Phylogenetic Species (CPS), which are almost completely consistent with Chinese Biological Species (CBS) of *Armillaria*, with just the exception of CBS K and CBS G which formed the same CPS on the Bayesian six-gene species tree. The CPS resolution of six-gene species tree was better than the IEL recognition of five protein-coding genes (*actin*, *h3h*, *hisps*, *rpb*1, and *tef*1α), because CPS *A. bruneocystidia* was only recognised by the six-gene species tree (Figure 1, Table 2). All the phylogenetic species are described below.

**Figure:**
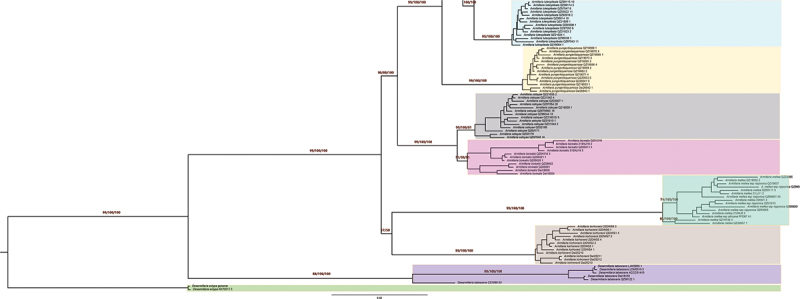
Figure 1. (Continued).

The Chinese phylogenetic species, *Armillaria algida*, was represented by 16 sequences and was strongly supported by the six-gene species tree (MPBP/MLBS/BPP = 94/100/100). The monophyletic CPS had two significant substructures: a large lineage (CBS O) strongly supported both by the six-gene species tree (MPBP/MLBS/BPP = 99/100/100) and by the three IELs (*actin*, *rpb*1, and *tef*1α), and a small lineage (only two sequences of Dai26847) strongly supported by the six-gene species tree (MPBP/MLBS/BPP = 98/100/100) and by the IEL of *hisps*. There is 37.5% incompatibility between the two lineages (Figure 3); it was not clear whether Dai 26847 represents a different phylogenetic species or subspecies (Figure 1, Figures S1, S5, S6, Table 2).

The Chinese phylogenetic species, *Armillaria amygdalispora*, was represented by 7 specimens and *A. violacea* by 14 specimens. Their reciprocal monophyly was strongly supported both by the six-gene species tree (MPBP/MLBS/BPP = 99/100/100 and 99/100/100) and by four IELs (*actin*, *hisps*, *rpb*1, and *tef*1α) and three IELs (*actin*, *hisps* and *rpb*1). These two CPS possessed the closest sibling relationships. *Armillaria violacea* had two phylogenetic subclades, whereas *A. amygdalispora* had no subclades (Figure 1, Figures S1–S3, S5, S6, Table 2).

The Chinese phylogenetic species, *Armillaria bruneocystidia*, was represented by 11 specimens and its monophyly was strongly supported by the six-gene species tree (MPBP/MLBS/BPP = 91/100/100). The monophyletic clade has no significant substructure (Figure 1, Table 2).

The Chinese phylogenetic species, *Armillaria gallica*, was represented by 15 specimens, and was strongly supported in the six-gene species tree (MPBP/MLBS/BPP = 99/100/100) and in four IELs (*actin*, *h3h*, *hisps* and *rpb*1). The monophyletic CPS has no phylogenetic substructure (Figure 1, Figures S1–S3, S5, Table 2).

The Chinese phylogenetic species, *Armillaria luteopileata*, was represented by 14 specimens. Its monophyly was strongly supported by the six-gene species tree (MPBP/MLBS/BPP = 99/100/100) and three IELs (*h3h*, *hisps*, and *tef*1α). The CPS has no phylogenetic substructure (Figure 1, Figures S2, S3, S6, Table 2).

The Chinese phylogenetic species, *Armillaria mellea*, was represented by 15 specimens and strongly supported by the six-gene species tree (MPBP/MLBS/BPP = 99/100/100) and all the IELs (Figures S1–S6). The heterothallic, *A. mellea*, and secondary homothallic, *A. mellea* ssp. *nipponica*, showed different sexuality and life cycles, and belonged to distinct biological species (Table S1), however, there was no phylogenetic differentiation between them and they belong to the same CPS. In addition, an African isolate (PFD87_41) and a Japanese isolate (QZ83003) were also clustered to the phylogenetic species (Figure 1, Figures S1–S6, Table 2).

The Chinese phylogenetic species, *Armillaria ostoyae* and *Armillaria borealis*, were represented by 14 specimens and 11 specimens, respectively. The reciprocal monophyly of the two clades was strongly supported by three IELs (*actin*, *hisps*, and *tef*1α) and weakly supported by the six-gene species tree (MPBP/MLBS/BPP = 99/100/81 for *A. ostoyae* and 51/30/95 for *A. borealis*). These two phylogenetic species have no internal phylogenetic structure and possess the closest phylogenetic relationship (Figure 1, Figures S1, S3, S5, S6, Table 2).

The Chinese phylogenetic species, *Armillaria pungentisquamosa*, was represented by 16 specimens; its monophyly was strongly supported both by the six-gene species tree (MPBP/MLBS/BPP = 99/100/100) and each IEL (*actin*, *h3h*, *hisps*, *rpb*1, and *tef*1α). This species has two phylogenetic subclades, the smaller one collected from the south of the Xizang's Plateau and the larger one from the east of the Xizang's Plateau, and may represent the most basal lineage of the “Gallica cluster” (Figure 1, Figures S1–S3, S5, S6, Table 2).

The Chinese phylogenetic species, *Armillaria sinapina*, was represented by 11 specimens and was strongly supported both by the six-gene species tree (MPBP/MLBS/BPP = 99/100/100) and four IELs (*actin*, *hisps*, *rpb*1, and *tef*1α). The species has no phylogenetic substructure and its closest phylogenetic relative is *A. sinensis* (Figure 1, Figures S1, S3, S5, S6, Table 2).

The Chinese phylogenetic species, *Armillaria sinensis*, was represented by 15 specimens, and was strongly supported both by the six-gene species tree (MPBP/MLBS/BPP = 87/100/100) and two IELs (*actin* and *hisps*). This monophyletic species has no phylogenetic substructure (Figure 1, Figures S1, S3, Table 2) and is phylogenetically closest to *A. sinapina*.

The Chinese phylogenetic species, *Armillaria tibetica*, was represented by 15 specimens, and its monophyly was strongly supported both by the six-gene species tree (MPBP/MLBS/BPP = 95/100/100) and three IELs (LSU rDNA, *hisps*, and *rpb*1). The clade included two subclades which have no significant difference (Figure 1, Figures S3, S5, Table 2).

The Chinese phylogenetic species, *Armillaria korhonenii*, was represented by 12 isolates, and its monophyly was strongly supported both by the six-gene species tree (MPBP/MLBS/BPP = 99/100/100) and each IEL of the protein-coding genes (*actin*, *h3h*, *hisps*, *rpb*1, and *tef*1α). The clade has no internal phylogenetic structure (Figure 1, Figures S1–S3, S5, S6, Table 2).

The Chinese phylogenetic species, *Desarmillaria tabescens*, was represented by 6 specimens; its monophyly was strongly supported both in the six-gene species tree (MPBP/MLBS/BPP = 99/100/100) and each IEL of the coding genes (*actin*, *h3h*, *hisps*, LSU rDNA, *rpb*1, and *tef*1α) (Figure 1, Figures S1–S6, Table 2).

### 3.3. Intersterility groups (ISGs)

**Figure 2.**
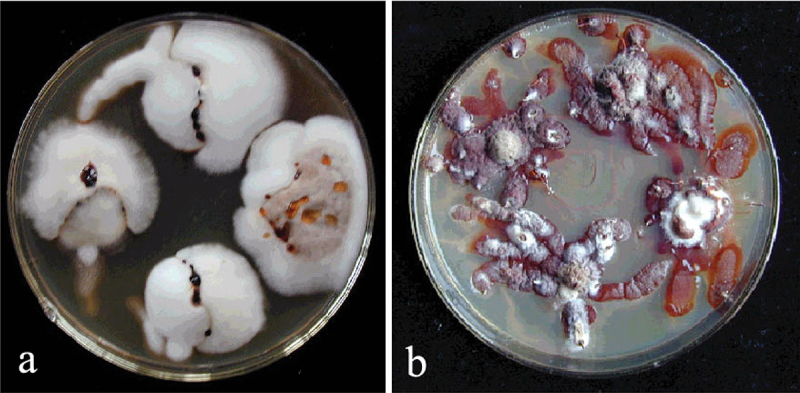
The results of the mating test. (a) The intersterile mating reactions. (b) The interfertile mating reactions.

A total of 30,340 mate pairing combinations among Chinese, European, and North American haploid isolates was carried out. Almost all pairing combinations between species displayed a typical intersterile mating reaction, viz. the paired haploid colonies retained white fluffy and formed an antagonistic black line, a few pairs of interspecies displayed incompatible mating reaction, viz. the paired haploid colonies retained white fluffy but did not form a black line. Almost pairing combination of intraspecies displayed a typical intersfertile mating reaction, viz. the paired haploid colonies fused and changed into brown crustose diploid morphology (Figure 2). According to the criteria of the mating test, a total of 16 intersterility groups (ISGs) of *Armillaria* in China was identified. All the haploid isolates identified by the mating test were used in the sequence (Table S1).

**Figure 3.**
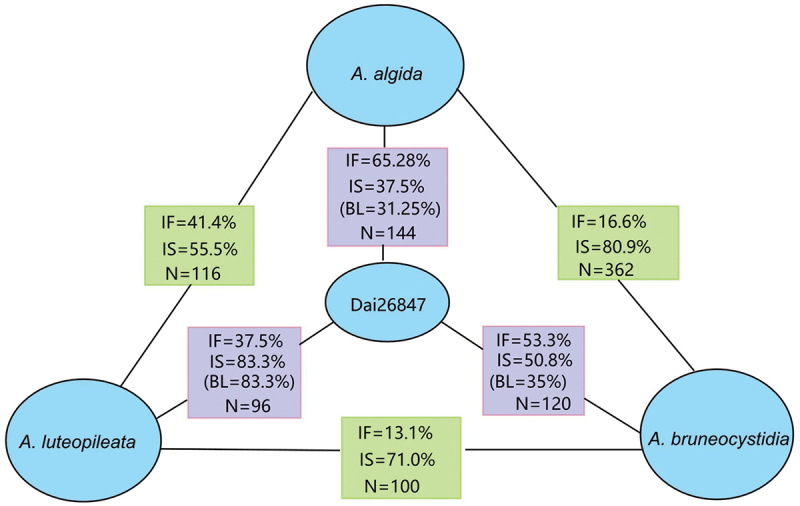
The mating relationships among Dai 26847, *Armillaria algida*, *A. bruneocystidia*, and *A. luteopileata*. IF: Interfertility; IS: Intersterility; BL: Antagonistic black line; N: Number of pair combinations.

The reproductive isolation among some species-pairs of *Armillaria* in China has not been completely established, or the gene flow between species has not been totally interrupted. The proportion of interfertile mating reactions between *A. algida* (CBS O) and *A. luteopileata* (CBS C) was 41.1%, that between *A. bruneocystidia* (CBS H) and *A. luteopileata* was 16.0%, that between *A. bruneocystidia* and *A. algida* was 16.6%, and that between *A. bruneocystidia* and *A. korhonenii* (CBS Q) was 13.1% (Figure 3, Table 3). It was worth knowing that gene flow of interspecies only exists in a specific subpopulation of a species in this study. All isolates of *A. luteopileata* from the high-elevation birch forest population in Changbai Mountain were compatible with *A. algida*, but all other isolates were incompatible with *A. algida*. The Dawei Mountain’s isolates of *A. korhonenii* were partially compatible with *A. bruneocystidia*, while the Ailao Mountain’s isolates of *A. korhonenii* were not. All mating interactions among the species in the Northern Hemisphere are summarised in Table 3.

**Table 3.** Mating interactions among biological species of *Armillaria* and *Desarmillaria* in the Northern Hemisphere.

	*Armillaria sinapina*			*A. gallica*			*A. luteopileata*			*A. ostoyae*			*A. sinensis*			*A. mellea* ssp. *nipponica*			*A. bruneocystidia*			*Desarmillaria tabescens*		
	IF (%)	IS (%)	N	IF (%)	IS (%)	N	IF (%)	IS (%)	N	IF (%)	IS (%)	N	IF (%)	IS (%)	N	IF (%)	IS (%)	N	IF (%)	IS (%)	N	IF (%)	IS (%)	N
*A. sinapina*	79.8	0.5	426	0.1	92.1	680	0	85.9	439	0	86.6	744	0	85.2	650	0	95.3	128	0	100	99	0	100	32
*A. gallica*				84	3.9	738	0.3	86	392	0	86	599	4.3	78.4	556	0	74.2	66	0	96.5	170	0	100	32
*A. luteopileata*							95.2	0	272	0	87	723	0	89.9	553	0	83.3	60	16	71	100	0	100	32
*A. ostoyae*										79.7	2.2	1,161	0	93	769	0	48.4	64	0	97.2	36	0	100	32
*A. sinensis*													84.9	3.6	365	0	78.9	76	0	97.1	68	0	100	80
*A. mellea* ssp. *nipponica*																0	0	20	0	86.1	36	0	96.9	32
*A. bruneocystidia*																			89.4	0.4	283	0	96.8	31
*D. tabescens*																						89.3	0	28
*A. tibetica*																								
*A. mellea*																								
*A. amygdalispora*																								
*A. borealis*																								
*A. violacea*																								
*A. algida*																								
*A. pungentisquamosa*																								
*A. korhonenii*																								
*A. calvescens*	0	86.5	96	0	87.5	16	3.6	71.4	28	0	50	32	0	92.5	80	0	92.6	27	0	100	36	0	0	0
*A. cepistipes*	0	97.2	72	3.7	80.2	81	0	84.1	44	0	100	45	0	96.7	61	0	95.5	44	0.9	96.4	112	0	0	0
*A. gemina*	0	94.2	86	0	73.3	15	0	72.1	43	0	50	32	0	100	74	0	81.8	44	0	97.2	36	0	0	0
*A. nabsnona*	0	95.5	44	0	100	8	0	63.6	22	0	75	16	0	92.5	40	0	100	22	0	94.4	18	0	0	0
*A. altimontana*	0	76.6	47	0	0	4	0	44.8	29	0	54.5	11	0	52.5	40	0	95.5	22	0	83.3	18	0	0	0
	***A.tibetica***			***A.mellea***			***A.amygdalispora***			***A.** **borealis***			***A.violacea***			***A.algida***			***A.pungentisquamosa***			***A.korhonenii***		
	**IF (%)**	**IS (%)**	**N**	**IF (%)**	**IS (%)**	**N**	**IF (%)**	**IS (%)**	**N**	**IF (%)**	**IS (%)**	**N**	**IF (%)**	**IS (%)**	**N**	**IF (%)**	**IS (%)**	**N**	**IF (%)**	**IS (%)**	**N**	**IF (%)**	**IS (%)**	**N**
*A. sinapina*	1.4	95.8	212	0	97.9	146	0	100	16	1.4	89.3	140	0	98.5	67	0	100	52	0	100	80	0	100	48
*A. gallica*	0	95.5	358	0	98.8	84	3.6	74.6	138	0	93.3	104	0	98.1	108	0	98.2	114	0	100	172	0	100	48
*A. luteopileata*	0	97	231	0	99	100	0	100	16	1.7	90	120	0	98.5	68	41.4	55.2	116	0	100	48	0	100	48
*A. ostoyae*	0	94.1	203	0	91.6	83	0	100	16	0	89.7	87	0	100	63	1.9	90.4	52	0	100	64	0	90.9	44
*A. sinensis*	0	93.5	619	0	92.9	154	0	100	48	0.3	92.5	319	0	92.9	156	0	99.6	232	0	100	162	0	100	48
*A. mellea* ssp. *nipponica*	0	94	67	5.4	58.9	241	0	0	0	0	81.5	81	0	100	27	0	93	43	0	100	40	0	100	24
*A. bruneocystidia*	0	98	717	0	93.7	95	3.4	84.5	116	0	96.9	129	0.9	99.1	116	16.6	80.9	362	0	100	223	13.1	84.7	176
*D. tabescens*	0	95.8	236	0	100	64	0	100	16	0	100	64	0	100	68	0	90.3	31	0	100	64	0	100	48
*A. tibetica*	85.4	1.3	2,309	0	97.9	236	0	99.5	212	0	87.6	622	0	90.7	507	0	98.6	352	0	97.5	402	0	96.9	64
*A. mellea*				84.9	7.3	192	0	98.1	104	0	95.6	183	0	98.9	90	0	97.3	74	0	100	88	0	95	40
*A. amygdalispora*							31.3	0	16	0	96.6	116	4.8	89.7	126	0	100	259	1	99	193	0	97.9	48
*A. borealis*										56.9	2.1	281	0	88.7	194	0	96.7	60	0	92.6	136	0	93.8	48
*A. violacea*													71.5	13.5	347	0	97.5	406	0	99.6	256	0	89.6	48
*A. algida*																82.7	12.2	254	0	100	179	0	97.9	48
*A. pungentisquamosa*																			72.9	6.3	48	0	100	64
*A. korhonenii*																						92.4	7.6	92
*A. calvescens*	0.5	82.4	188	0	98.5	68	0	100	16	0	91.3	80	0	97.7	44	0	72.2	18	0	100	24	0	100	16
*A. cepistipes*	0	99.2	236	0	95.6	68	0	100	20	0	94	100	0	94.6	56	0	100	24	0	100	32	0	100	44
*A. gemina*	0	95.4	195	0	97.1	68	0	100	16	0	100	56	0	98.1	53	0	89.5	19	0	100	16	0	0	0
*A. nabsnona*	0	90.7	97	0	100	34	0	100	8	0	100	40	0	100	28	0	80	10	0	100	8	0	0	0
*A. altimontana*	0	59.1	88	0	100	22	0	66.7	6	0	58.3	24	0	100	25	0	100	6	0	100	8	0	0	0

IF: Interfertility percentage, the proportion of an obviously crustose diploid colony was formed in the mating test. IS: Intersterility percentage, the proportion of an obviously black demarcation line was formed or both sides remained a white fluffy colony in the mating test. N: Number of pairing combinations.

A specimen (Dai26847) collected from Medog County of Xizang Autonomous Region, the southern Himalayas, showed simultaneously a partial intersterility with *Armillaria algida*, *A. bruneocystidia*, and *A. luteopileata*; the proportions of antagonistic black lines are 31.25%, 35%, and 83.3%, respectively. Considering that it possessed the closest mating relationships (65.28% interfertility ratio) and closest phylogenetic relationships on the Bayesian multigenic species tree with *A. algida*, we tentatively identified Dai26847 as *A. algida* (Figures 1 and 3).

### 3.4. Statistical comparisons of basidiospore micromorphology

**Figure 4.**
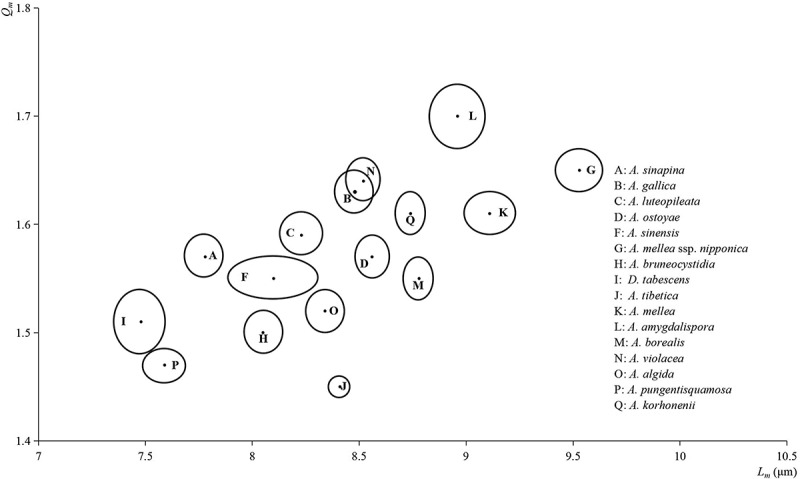
The basidiospore confidence interval of Chinese *Armillaria* and *Desarmillaria* species. The center of the ellipse was determined by *L*_*m*_ and *Q*_*m*_, and the radius of the ellipse was determined by the error limits of length and Q value of spore population.

The length and shape (Q value) of the basidiospores of studied specimens from all different locations were measured, and a significant test of sampled basidiospores means between species both in length or Q value was performed. The length and Q value of basidiospores were significantly different in each pair of the biological species, with the exception of an insignificant difference between *Armillaria gallica* and *A. violacea* (Table 4). In addition, on the basis of the mean of length (*L*_*m*_), Q value (*Q*_*m*_), and their error limits (EL), the statistical differences among species are displayed in Figure 4. This result demonstrated that most Chinese species of *Armillaria* can be distinguished according to the population morphology of the basidiospore. It should be paid attention that the mean plus error limit accurately displayed the estimated range of the population means of basidiospores at 95% confidence level and are very useful statistical values in the classification of the Agaricales.

**Table 4.** The statistical data on length and Q value of basidiospores of *Armillaria* and *Desarmillaria* species.

Species*	Length	Q value	n
*Armillaria algida*	8.34 ± 0.09	1.52 ± 0.02	331
*A. amygdalispora*	8.96 ± 0.13	1.70 ± 0.03	120
*A. borealis*	8.78 ± 0.07	1.55 ± 0.02	390
*A. bruneocystidia*	8.05 ± 0.09	1.50 ± 0.02	235
*A. gallica*	8.48 ± 0.09	1.63 ± 0.02	285
*A. korhonenii*	8.74 ± 0.07	1.61 ± 0.02	490
*A. luteopileata*	8.23 ± 0.10	1.59 ± 0.02	314
*A. mellea*	9.11 ± 0.12	1.61 ± 0.02	287
*A. mellea* ssp. *nipponica*	9.53 ± 0.11	1.65 ± 0.02	429
*A. ostoyae*	8.56 ± 0.08	1.57 ± 0.02	365
*A. pungentisquamosa*	7.59 ± 0.08	1.47 ± 0.02	300
*A. sinapina*	7.78 ± 0.09	1.57 ± 0.02	243
*A. sinensis*	8.10 ± 0.21	1.55 ± 0.02	332
*A. tibetica*	8.41 ± 0.05	1.45 ± 0.01	657
*A. violacea*	8.52 ± 0.08	1.64 ± 0.02	210
*Desarmillaria tabescens*	7.93 ± 0.10	1.40 ± 0.02	218

*Interspecies and inter-subspecies displayed extremely significant difference according to significant test (*P* < 0.01) excluding that between *A. gallica* and *A. violacea*.

### 3.5. Taxonomy

**Figure 5.**
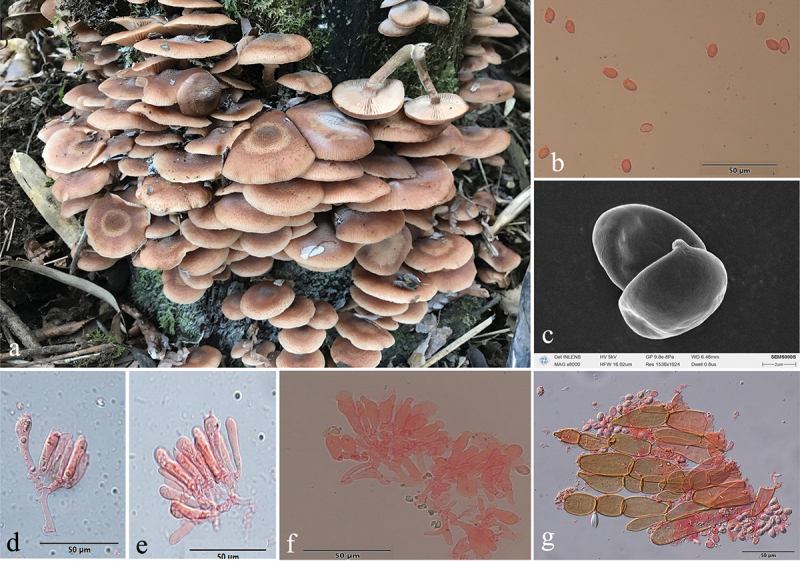
*Armillaria algida*. (a) Basidiomata (QZ19095). (b) Basidiospores. (c) Basidiospores under SEM. (d) Basidia and basidioles. (e) Clamp connection at the base of basidium and basidioles. (f) Cheilocystidia. (g) Hyphae of pileal squamules (QZ19095).

***Armillaria algida*** G.F. Qin, J. Zhao, W.M. Qin & Y.C. Dai, sp. nov. Figure 5

*MycoBank*: MB 836020.

Chinese Biological Species (CBS) O.

Chinese Phylogenetic Species (CPS) *Armillaria algida*.

*Etymology*: *Algida* (Lat.): refers to the species fruiting in the cold environment.

*Holotype*: China, Hubei Province, Wufeng County, Houhe Nature Reserve, Yangzixi, 30°04' N, 110°32' E, elev. 1,700 m, 21 October 2019, G.F. Qin (IPF QZ19095).

*Descriptions*: Basidiomata small to medium-sized, solitary, fasciculate to compactly caespitose, overall a reddish brown to rust brown with black-brown scales. Pilei 1.5–7.0 cm in diam., 0.4–1.2 cm thick, at first convex or hemispherical, then plano-convex to applanate or slightly depressed, sometimes umbonate; usually dry; orange-white (6A2), pale orange (5A3), orange-grey (6B2), greyish orange (6B3), greyish red (7B4), light brown (6D4, 7D5), brown (6E4, 7E4, 7E6), dark brown (6F7) at the margin, greyish orange (6B4), light brown (6D4), brown (6E4, 7E5–7E6), reddish brown (8E6), dark brown (6F4–6F5–6F6, 7F4–7F5) at the centre in youth; greyish orange (6B4), brownish orange (6C3–C4–7C4–7C5), light brown (6D3–6D4–7D4–7D5), brown (6E4–6E5) at the margin; greyish brown (6F3), brown (6E5, 7E4), dark brown (6F4–6F5, 7F4) at the centre when mature. Pileal scales long stiff erect hairs (0.2–0.4 cm) at the centre, light brown (6D46D5), brown (6E4–6E5, 7E4–7E5), dark brown (6F5) at the margin, brown (6E4), dark brown (6F4–6F5, 7F4–7F5) at the centre, distributed over entire pileal surface but more concentrated and erect towards the centre, more dispersed and flattened at the margin. Margin entire, incurved, or straight, usually with striations when mature.

Lamellae sinuate or subdecurrent, sometimes with striped ridges downwards to annulus, orange-white (5A2–6A2), pale orange (6A3), greyish orange (6B3) when young, greyish orange (5B4–6B3), brownish orange (6C4–6C5), light brown (6D5–7D5), brown (7E6), dark brown (6F5) when mature, subdistant, lamellulae present, margin smooth.

Stipe central, 3–9 cm long, 0.3–0.7 cm in diam. at the apex, cylindrical, rarely broadening at base; above the annulus orange-white (6A2), pale orange (6A3), greyish orange (6B3), the middle portion greyish orange (6B3), brownish orange (6C3), greyish brown (6D3), the base brownish orange (6C3–6C4), light brown (6D4), greyish brown (6E3–6F3) when young; above the annulus greyish orange (5B5, 6B3, 6B4), brownish orange (6C3, 6C4, 7C4), light brown (6D4, 7D4), the middle portion brownish orange (6C3, 6C5), light brown (6D4), brown (6E4, 7E4), the base brownish orange (6C3), light brown (6D4–6D5), brown (6E4–6E5), dark brown (6F4–7F4) when mature; covered with white fibrillose veil remnants; sometimes resembling snake-skin markings.

Annulus arachnoid, thin, ephemeral, white, or nearly white.

Basidia clavate 23–46 × 6.5–11 μm, upper portion slightly constricted or constriction inconspicuous, with a clamp connection at the base, four-sterigmate, thin- to slightly thick-walled, yellowish green to brownish yellow. The pigments of the basidia disappeared gradually in KOH. Sometimes with brownish yellow crassobasidia (≤1 μm thick). Sterigmata 2–6 (4.15 ± 0.73) μm.

Basidiospores white in mass, ellipsoid, lacking a suprahilar depression, [331/11/10] (6.9)7.0–10.0(11.2) × (4.5)4.9–6.5(7.0) μm, [Q = (1.18)1.25–1.80(1.96), *Q*_*m*_ = 1.52 ± SD0.14]; thin- to thick-walled (≤1 μm), hyaline, or honey yellow, smooth, cyanophilous, inamyloid, with a prominent apiculus.

Cheilocystidia 13.0–47.0 × 4.5–11.0 μm, nearly cylindrical, with a clamp connection at the base, usually with irregular papilla at the apex; nearly hyaline, thin-walled.

Pleurocystidia absent.

Pileal squamules were composed of more or less parallel, cylindrical, thin- to thick-walled (≤2 μm) hyphae, simple septate, greenish yellow to brownish yellow, 17.0–127.0 × 5.0–28.0 μm.

*Specimens examined*: China, Hubei Wufeng County, Houhe Nature Reserve, Yangzixi, on angiosperm stump, 21 October 2019, Guo-Fu Qin, QZ19091, QZ19095, Guo-Fu Qin, QZ19102; on fallen angiosperm wood, 21 October 2019, Guo-Fu Qin, QZ19080; on rotten wood, 21 October 2019, Guo-Fu Qin, QZ19081; on fallen trunk of *Acer* sp., 21 October 2019, Guo-Fu Qin, QZ19085; on stump of *Morus alba*, 21 October 2019, Guo-Fu Qin, QZ19103; on fallen wood of *Pyrus* sp., 21 October 2019, Guo-Fu Qin, QZ19087; on stump of *Pyrus* sp., 21 October 2019, Guo-Fu Qin, QZ19100, QZ19101; Sichuan Province, Meishan, Emei Mts., on dead wood of *Castanopsis sempervirens*, 19 October 2002, Yu-Cheng Dai, QZ02072; on dead angiosperm standing tree, 31 October 2019, Guo-Fu Qin, QZ19106; Xizang Autonomous Region, Medog County, Nyingchi, on rotten wood of *Abies*, 25 October 2023, Yu-Cheng Dai, Dai26847.

*Remarks*: *Armillaria algida*, *A. bruneocystidia*, *A. pungentisquamosa*, *A. tibetica*, and *A. violacea* have an overlapping distribution area, However, *A. bruneocystidia*, *A. tibetica*, and *A. violacea* have white fibrillose scales, *A. pungentisquamosa* has a slender pale brown basidiomata and spiky dark scales. *Armillaria algida* and *A. violacea* may occur at the same time in the same location, but they have different scales and different spore shapes (ellipsoid vs elongate).

**Figure 6.**
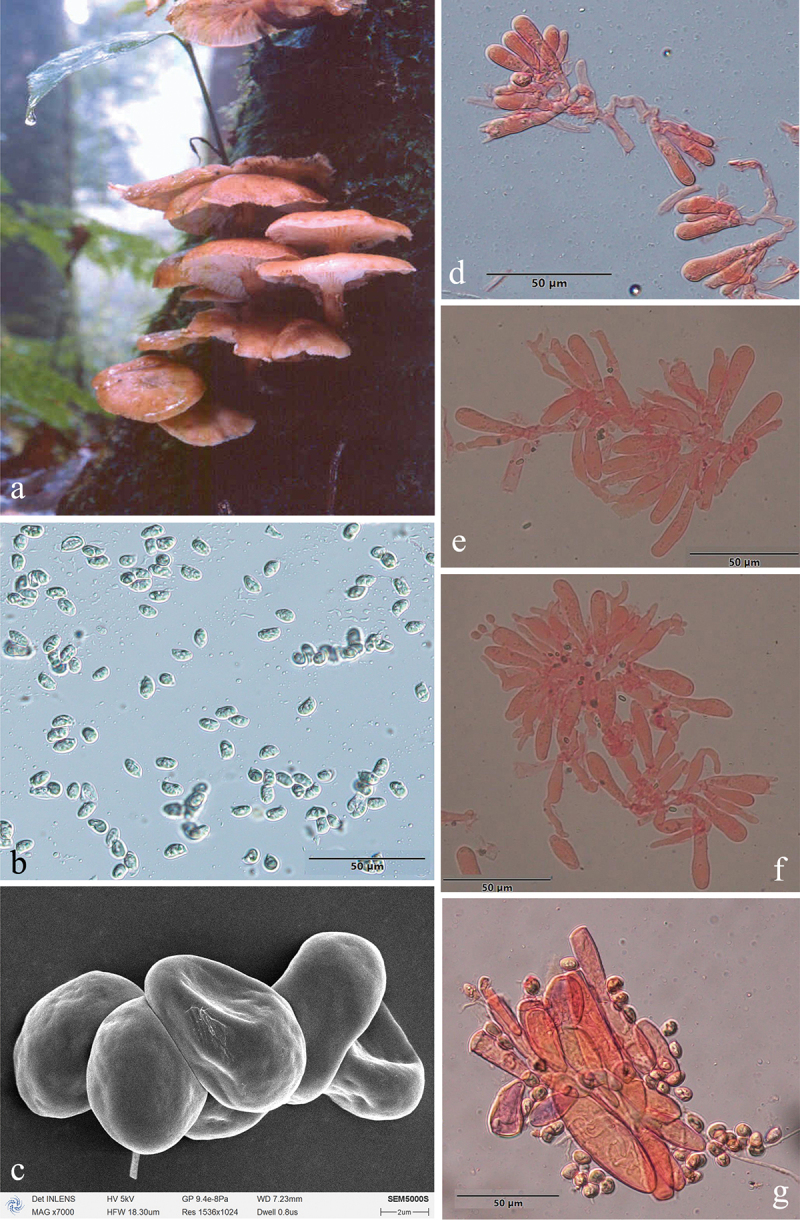
*Armillaria amygdalispora*. (a) Basidiomata (QZ00126). (b) Basidiospores. (c) Basidiospores under SEM. (d) Basidia and basidioles. (e) Cheilocystidia. (f) Basidia, basidioles, and cheilocystidia. (g) Hyphae of pileal squamules (QZ00125).

***Armillaria amygdalispora*** H.C. Wang, G.F. Qin, J. Zhao & Y.C. Dai, sp. nov. Figure 6

*MycoBank*: MB 831858.

Chinese Biological Species (CBS) L.

Chinese Phylogenetic Species (CPS) *Armillaria amygdalispora.*

*Etymology*: *amygdalispora* (Lat.): refers to the almond-shaped basidiospores.

*Holotype*: China, Guizhou Province, Suiyang County, Kuankuoshui Nature Reserve, 28°02’ N, 107°01’ E, elev. 1,550 m, 17 September 2000, J. Zhao & S.M. Tian (IPF QZ00125).

*Descriptions*: Basidiomata small to medium-sized, collybioid, imbricate-caespitose on dead trees, concrescent at the base, sometimes solitary. Pilei 2.6–6.9 cm in diam., 0.2–0.9 cm thick, at first hemispherical-convex to convex, then plano-convex to applanate, sometimes more or less depressed; dry, brownish orange (7C3–7C6), light brown (7D4–7D7), brown (7E4–7E8). Margin entire, usually slightly incurved, with striations. Pileal scales brown (6E3–6E6–7E3–7E8), dark brown (6F3–6F6–7F3–7F8), distributed over entire surface but denser and suberect towards the centre, more dispersed and flattened towards the margin.

Lamellae adnate with a decurrent tooth, white to nearly white when young, then pale red (7A3), greyish red (7B3–7B5), brownish orange (7C3–7C6), light brown (7D4–7D8) when mature, subdistant, lamellulae present, margin smooth.

Stipe central, 2.5–6 cm long, 0.4–1 cm in diam. at the apex, clavate or bulbous, orange-white (6A2) above the annulus, the middle brown (7E6), the base dark brown (7F7), with white fibrillose veil remnants, resembling snake-skin markings.

Annulus cortinate, thin and delicate, usually ephemeral, white, or nearly white.

Basidia clavate, 8–35 × 5.5–8 μm, upper portion slightly constricted or constriction inconspicuous, with a clamp connection at the base, four-sterigmate, usually thin- to slightly thick-walled, yellowish green to brownish yellow. The pigments of basidia disappear gradually in KOH, sometimes brownish yellow crassobasidia (≤1.5 μm thick) are formed and crassobasidia with septa when lamellae develop abnormally. *Sterigmata* 3–5 [4 ± SD1] μm.

Basidiospores white in mass, almond-shaped, ellipsoid, lacking a suprahilar depression, [120/4/4] (7.2)7.8–10.2(11.0) × (4.2)4.8–6.2(6.3) μm, [Q = (1.29)1.30–2.08(2.21), *Q*_*m*_ = 1.70 ± SD0.19]; thin- to thick-walled (≤1.2 μm), nearly hyaline, to brownish yellow, smooth, cyanophilous, inamyloid, with a prominent apiculus.

Cheilocystidia 20–40 × 4.5–9.5 μm, polymorphic, clavate, fusiform, cylindrical, elliptical, ovoid, with a clamp connection at the base, usually with papilla or irregular branched papilla at the apex; usually thin- to slightly thick-walled (≤0.2 μm), nearly hyaline, usually compactly forming a sterile lamella edge but easily broken and disappearing.

Pleurocystidia absent.

Pileal squamules are composed of parallel, cylindrical, slightly thick- to thick-walled (≤2.5 μm) hyphae, without clamps at septa, grey to brownish yellow, 20–94 × 4.5–25 μm.

*Specimens examined*: China, Guizhou Province, Suiyang County, Kuankuoshui Nature Reserve, on stump of *Cyclobalanopsis multinevis*, 17 September 2000, Jun Zhao, Shu-Min Tian, QZ00125, QZ00126; Hunan Province, Tianzishan County, Zhangjiajie Forest Park, on ground, 16 September 1999, M. Harkonen, MH_K71; Sichuan Province, Qingchengshan Mts., Dujiangyan, 20 October 2002, Yu-Cheng Dai, QZ08202.

*Remarks*: *Armillaria amygdalispora* is a rare species, found only in the primeval forest of Guizhou, Sichuan, Hubei, and Hunan Provinces. It can be separated from other Chinese members of the “Gallica superclade” by its brownish orange to light brown basidiomata, branched cheilocystidia plus almond-shaped spores (*Armillaria tibetica* with branched cheilocystidia but does not have almond-shaped spores).

**Figure 7.**
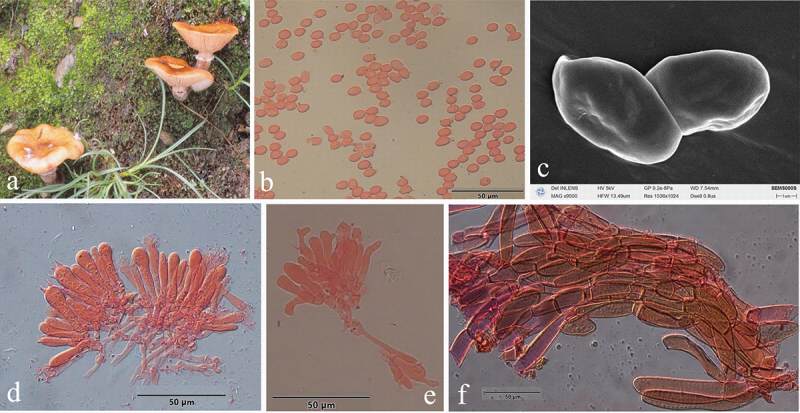
*Armillaria bruneocystidia*. (a) Basidiomata (QZ00123). (b) Basidiospores. (c) Basidiospores under SEM. (d) Basidia and basidioles. (e) Cheilocystidia. (f) Hyphae of pileal squamules (QZ19078).

***Armillaria bruneocystidia*** H.C. Wang, G.F. Qin, J. Zhao & Y.C. Dai, sp. nov. Figure 7

*MycoBank*: MB 831857.

Chinese Biological Species (CBS) H.

Chinese Phylogenetic Species (CPS) *Armillaria bruneocystidia.*

*Etymology*: *bruneocystidia* (Lat.): refers to the brown colour of the cystidia.

*Holotype*: China, Sichuan Province, Emei Mountain, Zhanglaoping, 29°34’ N, 103°21’ E, elev. 1,700 m, 16 October 2019, G.F. Qin (IPF QZ19078).

*Descriptions*: Basidiomata small to large-sized, solitary or caespitose or loosely grouped on fallen trunks and woody debris. Pilei 1.8–15.4 cm in diam., 0.5–2.0 cm thick, at first hemispherical-convex to convex, with or without small pointed umbo, then plano-convex, applanate, depressed; usually dry; orange-white (6A2), light brown (5D4), light brown (6D5–6D6) at the margin, light brown (6D4), yellowish brown (5F4), brown (6E5–6E6) at the centre when young; greyish orange (6B5), brownish orange (5C4), light brown (6D4–6D5), brown (6E6), reddish brown (7E5–8E4) at the margin, greyish orange (6B4), yellowish brown (5E4), light brown (6D7), brown (6E4–6E6), reddish brown (7E5), dark brown (6F4–6F6–7F5) at the centre when mature. *Pileal scales* hairy, brownish grey (6E2–6E5), yellowish brown (5E4), brown (6E5), dark brown (6F4–6F6–7F5), distributed over entire surface of cap but more concentrated and suberect towards the centre, more dispersed and flattened towards the margin. Margin entire, usually incurved, usually striate.

Lamellae adnate with decurrent tooth or sinuate, usually with striped ridges downwards to annulus, white, yellowish white (4A2), orange-white (5A2–6A2), pale orange (6A3) when young, then pale orange (6A3), greyish orange (5B3), brownish orange (6C4), light brown (6D4–7D4–7D6) when mature, subdistant, lamellulae present, margin smooth.

Stipe central, 2.5–12 cm long, 0.4–1.2 cm in diam. at the apex, cylindrical or clavate; white, orange-white (5A2–6A2), greyish orange (6B3), brownish orange (6C3–6C4), light brown (6D4–6D5), reddish brown (8D5), brown (6E4), dark brown (6F4) above the annulus; the middle yellowish white (4A2), greyish orange (5B3), brownish orange (6C3–6C4), light brown (6D5), reddish brown (8D5), brown (6E4–6E5), dark brown (6F4), the base pale yellow (4A3), brownish orange (5C3), light brown (6D3–6D4–6D5), reddish brown (8D5), dark brown (6F4–6F5–6F6–7F4); usually covered with nearly white, brownish grey (6E2) fibrillose veil remnants, sometimes resembling zigzag markings or a white membrane, occasionally the stipe base with light yellow (4A4) fine fibers.

Annulus arachnoid or cortinate, single, thin, usually ephemeral, white or nearly white, sometimes outer margin brown (6E2) or dark brown (7F3–7F8).

Basidia clavate, 14–50 × 3–16 μm, upper portion constricted or constriction inconspicuous, with a clamp connection at the base, four-sterigmate, thin- to slightly thick-walled, yellowish green to brownish yellow. The pigments of the basidia disappear gradually in KOH, sometimes brownish yellow crassobasidia (≤1.3 μm) form when the lamellae develop abnormally. *Sterigmata* 2–6 μm.

Basidiospores ivory white in mass, ellipsoid, lacking a suprahilar depression, [235/12/10] (6.0)7.0–9.0(10.0) × (4.0)4.9–6.0(7.0) μm, [Q = (1.00)1.17–1.80(2.00), *Q*_*m*_ = 1.50 ± SD0.14]; thin- to thick-walled (≤1 μm), nearly hyaline to brownish yellow, smooth, cyanophilous, inamyloid, with a prominent apiculus.

Cheilocystidia 14.0–50.0 × 3.0–16.0 μm, polymorphic, viz., fusiform, elliptical, cylindrical, ovoid, clavate, with a clamp connection at the base, usually with papilla or irregular slender papilla at the apex; thin- to slightly thick-walled (≤0.4 μm), occasionally local thick-walled (0.9 μm); nearly colorless to brownish yellow, usually forming a compact sterile lamella edge but it is easily broken and disappears.

Pleurocystidia absent.

Pileal squamules composed of more or less parallel, cylindrical, slightly thick-walled (≤0.9 μm) hyphae, simple septate, grey to brownish yellow, 14.0–170.0 × 6.0–38.0 μm.

*Specimens examined*: China, Sichuan Province, Emei Mts., on stump of *Cunninghamia lanceolata*, 16 October 2019, Guo-Fu Qin, QZ19074; on dead standing angiosperms tree, 16 October 2019, Guo-Fu Qin, QZ19075, QZ19078; Yunnan Province, Naxi County, Yulong Snow Mts., on rotten wood of *Picea likiangensis*, 11 June 1999, K. Korhonen, Yu-Cheng Dai, QZ99011; 20 June 2000, Guo-Fu Qin, Jun Zhao, QZ00005; on fallen trunk of *Picea likiangensis*, 20 June 2000, Guo-Fu Qin, Jun Zhao, QZ00003; on stump of *Picea likiangensis*, 20 June 2000, Guo-Fu Qin, Jun Zhao, QZ00004, QZ00006; on fallen angiosperm trunk, 20 June 2000, Guo-Fu Qin, Jun Zhao, QZ00007; on base of angiosperm tree, 20 June 2000, Guo-Fu Qin, Jun Zhao, QZ00008; on fallen trunk of *Abies ferreana*, 20 June 2000, Guo-Fu Qin, Jun Zhao, QZ00009; on rotten wood debris, 20 June 2000, Guo-Fu Qin, Jun Zhao, QZ00010, QZ00011, QZ00012, QZ00013, QZ00014, QZ00015; Shangri-La County, Tianshengqiao, on rotten angiosperm wood debris, 17 September 2000, Jun Zhao, Shu-Min Tian, QZ00123, QZ00124.

*Remarks*: *Armillaria bruneocystidia* was collected from Yunnan and Sichuan Provinces only, and it occurs in the same areas as *A. mellea* s. str., but the latter differs from the former by golden yellow basidiomata with a persistent membranous annulus, and its basidia have simple septa. *Armillaria bruneocystidia* and *A. tibetica* share similar morphology, grow on the same hosts, and have an overlapping distribution, but *A. tibetica* has broader spores, *Q*_*m*_ = 1.45, and its fruiting peak is in October, whereas *A. bruneocystidia Q*_*m*_ = 1.50 and its fruiting peak is in June (Figure 11).

**Figure 8.**
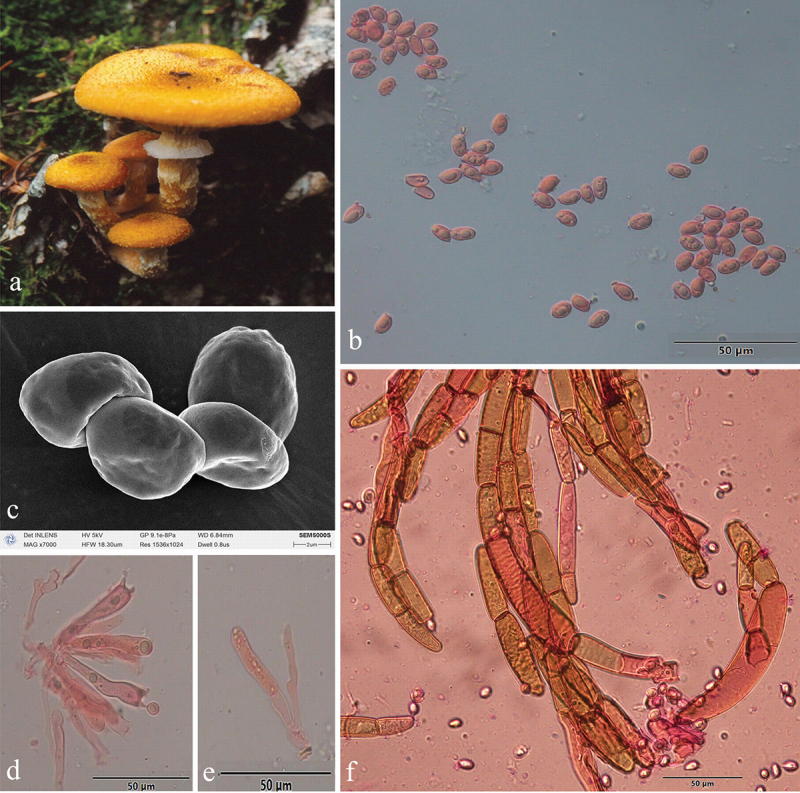
*Armillaria luteopileata*. (a) Basidiomata (QZ96014). (b) Basidiospores. (c) Basidiospores under SEM. (d) Basidia and basidioles. (e) Cheilocystidia. (f) Hyphae of pileal squamules (QZ21023).

***Armillaria luteopileata*** G.F. Qin, J. Zhao, W.M. Qin & K. Korhonen, sp. nov. Figure 8

*MycoBank*: MB 831855.

Chinese Biological Species (CBS) C.

Chinese Phylogenetic Species (CPS) *Armillaria luteopileata*.

*Etymology*: *luteopileata* (Lat.): refers to the yellow pileal surface.

*Holotype*: China, Jilin Province, Changbai Mts., Yuehualin, coniferous forest, 42°03’ N, 128°03’ E, elev. 1,850 m, 8 September 2021, G.F. Qin (IPF QZ21023).

*Descriptions*: Basidiomata small to medium-sized, solitary to clustered, caespitose or loosely grouped on fallen trunks and branches, generally golden yellow with brown scales. Pilei 1.7–7.5 cm in diam., 0.5–1.5 cm thick, at first hemispherical-convex to convex, then plano-convex to applanate, sometimes slightly depressed; usually dry; brownish yellow (5C7), light brown (5D6–6D5), brown (6E4–6E6) at the margin, greyish yellow (4B6), yellowish brown (5E6–5E7), brown (6E5) at the centre when young; light yellow (4A5) or reddish yellow (4A6), greyish orange (5B4–5B6), brownish orange (5C6), brownish yellow (5C7), brownish orange (6C5), golden brown (5D6–5D7) or light brown (6D5–6D6), yellowish brown (5E6), brown (6E4) at the margin, light yellow (4A4), reddish yellow (4A6), light orange (5A4), greyish orange (5B5), golden brown (5D7–5E7), yellowish brown (5E4–5E7–5E8), light brown (6D5), brown (6E6), dark brown (6F4–6F6) at the centre when mature. Pileal scales fibrillose, yellowish brown (5E5), brown (6E5–6E6), dark brown (6F4–6F5), distributed over entire pileal surface but more concentrated and suberect towards the centre, more dispersed and flattened towards the margin. Margin entire, sometimes slightly incurved, usually striate, sometimes with white velar remnants.

Lamellae sinuate or decurrent, sometimes with striped ridges down to annulus, white to yellowish white (4A2), orange-white (5A2), yellowish grey (4A3) when young, then pale orange (5A3) to orange-grey (6B3) when mature, subdistant, lamellulae present, margin smooth.

Stipe central, 3–10 cm long, 0.3–2 cm diam. at the apex, cylindrical or clavata, white (4A1), yellowish white (4A2), pale orange (5A3), orange-grey (5B3–6B3), greyish orange (6B4), brownish orange (7C5) above the annulus; the middle greyish orange (5B3–5B4), brownish orange (5C4–5C5), light brown (6D4), brown (6E4); the base greyish orange (5B4–5B5), brownish orange (5C5–6C3), light brown (5D7–6D4–6D5), yellowish brown (5E4–6E4), brown (6E4–6E5–6E6) to dark brown (6F6); densely covered with white, pale yellow (3A3), light yellow (3A4–4A4) flocculose veil remnants, reminiscent of snakeskin markings.

Rhizomorphs thick, black, beneath the bark of trunks and roots, branching dichotomously in nature.

Annulus membranous or cortinate, persistent or ephemeral, usually a membranous annulus if the outer veil leaves remnants on the margin of the pileus, inner veil white, outer margin pale yellow (2A3–3A3–4A3), light yellow (3A4–3A5–4A4), reddish yellow (4A6–4A7), occasionally with yellowish brown (5E4) scales; sometimes a cortinate annulus when the veils are broken at the junction with stipe.

Basidia clavate, 10.0–45.0 × 6.0–12.0 μm, upper portion constriction inconspicuous or occasionally constricted, with a clamp connection at the base, four-sterigmate, thin- to slightly thick-walled, yellowish green to brownish yellow. The pigments of the basidia dissolve gradually in KOH, sometimes brownish yellow crassobasidia (≤1.5 μm thick) basidia are formed when the lamellae develop abnormally. Sterigmata 1.5–6 μm (4.13 ± SD0.57).

Basidiospores ivory white in mass, ellipsoid, lacking a suprahilar depression, [314/20/11] (4.0)7.0–9.8(10.5) × (4.0)4.5–6.0(7.5) μm, [Q = (1.00)1.27–1.89(2.00), *Q*_*m*_ = 1.59 ± SD0.15]; thin- to slightly thick-walled (≤1 μm), hyaline to brownish yellow, smooth, cyanophilous, inamyloid, with a prominent apiculus.

Cheilocystidia 8–32 × 4.5–13 μm, polymorphic, viz., fusiform, elliptical, cylindrical, ovoid, with a clamp connection at the base, sometimes with papilla or irregular slender papilla at the apex; hyaline, thin- to slightly thick-walled (≤0.4 μm), forming a compact sterile lamella-edge but it is easily broken and disappearing.

Pleurocystidia absent.

Pileal squamules composed of more or less parallel, cylindrical, slightly thick- to thick-walled (≤1 μm) hyphae, simple septate, grey to brownish yellow, 12.0–122.0 × 4.0–32.0 μm.

*Specimens examined*: China, Heilongjiang Province, Dailing Area, Liangshui Forestry Farm, on root of living *Betula platyphylla*, 6 September 1999, Jun Zhao, Shu-Min Tian, QZ99109; on fallen trunk of *Betula platyphylla*, 6 September 1999, Jun Zhao, Shu-Min Tian, QZ99110; on living tree of *Picea koraiensis*, 6 September 1999, Jun Zhao, Shu-Min Tian, QZ99111; on rotten gymnosperm wood, 7 September 1999, Jun Zhao, Shu-Min Tian, QZ99114, QZ99115; Jilin Province, Antu County, Changbaishan Nature Reserve, on fallen trunk of *Abies nephrolepis*, 28 August 1996, Guo-Fu Qin, QZ96018; 2 September 1996, Guo-Fu Qin, QZ96036; 3 September 1996, Guo-Fu Qin, QZ96038; 8 September 2021, Guo-Fu Qin, QZ21009; on fallen trunk of *Betula ermamii*, 28 August 1996, Guo-Fu Qin, QZ96014; on rotten trunk of *Betula ermanii*, 8 September 2021, Guo-Fu Qin, QZ21024; on rotten wood debris of *Betula ermanii*, 8 September 2021, Guo-Fu Qin, QZ21023; on fallen trunk of *Betula platyphylla*, 16 September 1997, Guo-Fu Qin, Jun Zhao, QZ97052; on fallen trunk of *Tilia amurensis*, 16 September 1997, Guo-Fu Qin, Jun Zhao, QZ97053; on fallen trunk of *Ulmus pumila*, 6 September 2019, Wen-Min Qin, Yu-Lian Wei, QZ19004; on rotten angiosperm wood, 30 August 1996, Guo-Fu Qin, QZ96027; 31 August 1996, Guo-Fu Qin, QZ96029; on fallen angiosperm trunk, 14 September 1997, Guo-Fu Qin, Jun Zhao, QZ97043.

*Notes*: *Armillaria luteopileata* sometimes occurs in the same forest as *A. ostoyae*, but can be easily distinguished by the colour of the fruit bodies (golden yellow vs. dark brown), the thickness of annulus (thin vs. thick) and the scales (white fibrillose vs. brown spinous). It also occurs in the same area as *A. sinensis* but can be differentiated because the latter has a tall and straight stipe, bulbous stipe-base, and monopodial rhizomorphs.

**Figure 9.**
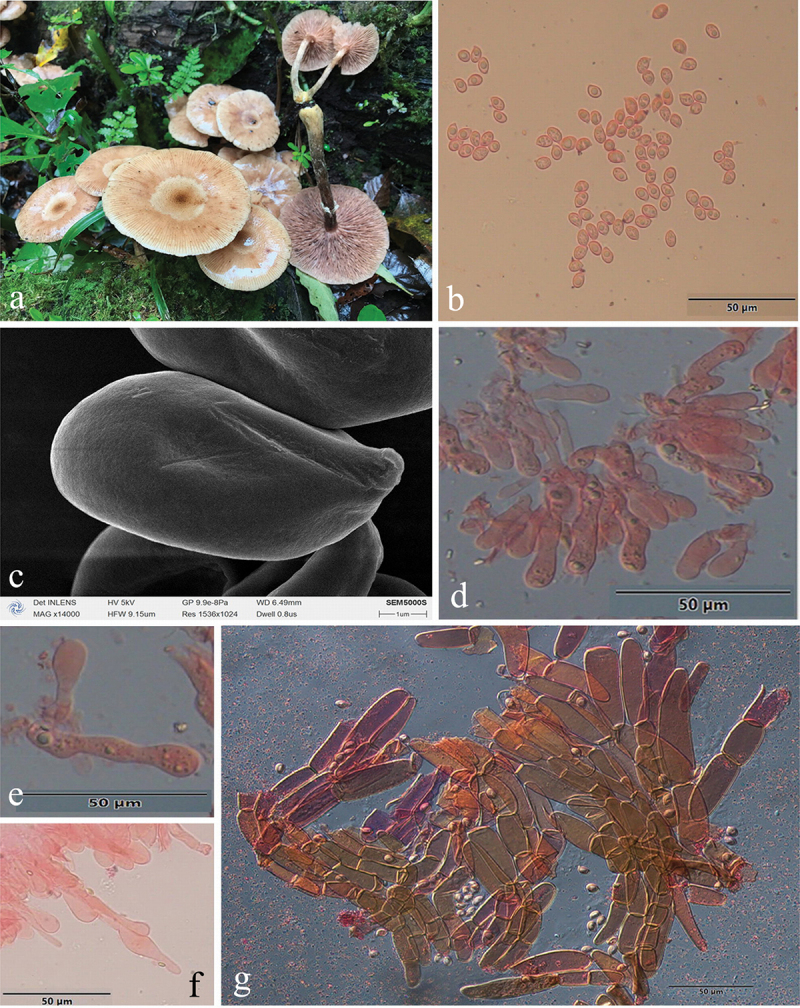
*Armillaria pungentisquamosa*. (a) Basidiomata (QZ19062). (b) Basidiospores. (c) Basidiospores under SEM. (d) Basidia and basidioles. (e) A clamp connection at the base of basidioles. (f) Cheilocystidia. (g) Hyphae of pileal squamules (QZ19062).

***Armillaria pungentisquamosa*** G.F. Qin, W.M. Qin, J. Zhao & K. Korhonen, sp. nov. Figure 9

*MycoBank*: MB 836021.

Chinese Biological Species (CBS) P.

Chinese Phylogenetic Species (CPS) *Armillaria pungentisquamosa.*

*Etymology*: *pungentisquamosa* (Lat.): refers to the scales being suberect and sharply pointed.

*Holotype*: China, Hubei Province, Hefeng County, Mulinzi Nature Reserve, Heiwanya, 30°02’ N, 110°12’ E, elev. 1,300 m, 12 October 2019, G.F. Qin (IPF QZ19062).

*Descriptions*: Basidiomata small to medium-sized, solitary, fasciculate to caespitose or loosely grouped on fallen trunks, standing stumps, and at the base of living trees. Pilei 2.0–10.0 cm in diam., 0.3–1.5 cm thick, at first convex with small pointed umbo, then plano-convex to applanate to depressed, usually with mammilla; usually dry; pale orange (5A3), light orange (5A4), greyish orange (5B4–6B3), brownish orange (5C3–5C5), yellowish brown (5E5–5E6) at the margin, brownish orange (5C5), light brown (5D4–5D5), yellowish brown (5E5–5F5–5F6), brown (6E6) at the centre when young; greyish orange (5B3–5B4–5B5), brownish orange (5C5), light brown (5D5–6D5–6D6), brown (6E5) at the margin, brownish orange (5C4–6C4), light brown (5D4), yellowish brown (5E4–5E5–5E6, 5F5–5F6), brown (6E5), dark brown (6F5–6F6) at the centre when mature. Pileal squamules spiky, with stiff erect hairs at the centre, light brown (5D5) to brown (6E5) at the margin, light brown (5D5–5E5), brown (6E5–6E6), dark brown (6F5–6F6) at the centre, distributed over entire surface of cap but more concentrated and suberect towards the centre, more dispersed and flattened towards the margin. Margin entire, incurved or straight, and striate.

Lamellae sinuate, sometimes subdecurrent or adnate with a decurrent tooth, with striped ridges downwards to annulus, at first orange-white (5A2–6A2), pale orange (5A3), greyish orange (6B3), light orange (5A4), brownish orange (6C2–6C3), then yellowish brown (5D5–5E5), brownish orange (6C4–6C5), light brown (6D5–6D6), brown (6E5–6E6), dark brown (6F5–6F6), subdistant, lamellulae present, margin smooth.

Stipe central, 2–11 cm long, 0.3–1.2 cm in diam. at apex, cylindrical when occurring in fasciculate groups, broader at base when occurring singly; greyish yellow (4B4), greyish orange (5B4) above annulus, the middle portion brownish orange (5C4–5C5), the base yellowish brown (5D5–5E4) when young; greyish orange (5B4–6B5), brownish orange (5C4–5C6–6C5), light brown (6D5–6D6), yellowish brown (5E5), brown (6E6) above annulus; the middle portion brownish orange (5C5), light brown (6D5–6D6), yellowish brown (5E5), brown (6E5–6E6), dark brown (6F5); the base light brown (6D5), brown (6E5), yellowish brown (5F5), dark brown (6F5–6F6) when mature; longitudinally striate, covered with white to greenish yellow fibrillose veil remnants, sometimes resembling zigzag markings; usually densely covered with greyish yellow (1B5–2C4–2C5), greyish green (29B5–30B4) floccules at base.

Annulus submembranous, sometimes arachnoid, thin, ephemeral; white or nearly white.

Rhizomorphs black, thick, cylindrical, monopodial branching in nature.

Basidia clavate 18.0–41.0 × 6.0–8.5 μm, upper portion constricted or constriction inconspicuous, with a clamp connection at the base, four-sterigmate, usually thin-walled, pale yellow to brownish yellow. The pigments of the basidia dissolve in KOH, sometimes brownish yellow crassobasidia (≤1 μm) are formed. Sterigmata 2–6.2 (4.63 ± SD0.89) μm.

Basidiospores ivory white in mass, ellipsoid, lacking a suprahilar depression, [300/9/9] (6.0)6.5–8.8(9.6) × (4.0)4.5–6.0(6.4) μm, [Q = (1.16)1.22–1.79(2.05), *Q*_*m*_ = 1.47 ± SD0.14]; thin- to thick-walled (≤1.0 μm), nearly colourless to pale yellow, smooth, cyanophilous, inamyloid, with a prominent apiculus.

Cheilocystidia 10–80 × 4–11 μm, polymorphic, viz., clavate, fusiform, cylindrical, with a clamp connection at the base, usually with irregular papilla or branched papilla at the apex; nearly colourless to yellowish brown, usually thin- to slightly thick-walled (≤0.5 μm), usually forming a compact sterile lamella edge that is easily broken and disappears.

Pleurocystidia absent.

Pileal squamules composed of more or less parallel, cylindrical, thin- to slightly thick-walled (≤1 μm) hyphae, clampless, brownish yellow, 15–98 × 5–29 μm.

*Specimens examined*: China, Hubei Province, Hefeng County, Mulinzi Nature Reserve, Changwan, on fallen angiosperm trunk, 13 October 2019, Guo-Fu Qin, QZ19069, QZ19070, QZ19071; Mulinzi Nature Reserve, Heiwanya, on dead stump of *Betula albosinensis*, 12 October 2019, Guo-Fu Qin, QZ19061; on fallen trunk of *Fagus* sp., 12 October 2019, Guo-Fu Qin, QZ19066; on fallen trunk of *Sycopsis sinensis*, 12 October 2019, Guo-Fu Qin, QZ19062; on fallen angiosperm trunk, 12 October 2019, Guo-Fu Qin, QZ19063; on rotten angiosperm root, 12 October 2019, Guo-Fu Qin, QZ19068; Wufeng County, Houhe Nature Reserve, on dead stump of *Cerasus pseudocerasus*, 10 October 2019, Guo-Fu Qin, QZ19055; on rotten fallen trunk of *Salix babylonica*, 10 October 2019, Guo-Fu Qin, QZ19060; on rotten angiosperm wood, 24 September 2004, Hai-Sheng Yuan, Yu-Lian Wei, QZ04047; 10 October 2019, Guo-Fu Qin, QZ19053; on rotten angiosperm stump, 10 October 2019, Guo-Fu Qin, QZ19058; on rotten angiosperm wood debris, 10 October 2019, Guo-Fu Qin, QZ19059; Sichuan Province, Meishan, Emei Mts., on fallen angiosperm trunk, 15 October 2019, Guo-Fu Qin, QZ19073; Xizang Autonomous Region, Motuo County, Nyingchi, on rotten wood of *Abies*, 25 October 2023, Yu-Cheng Dai, Dai26842, Dai26843.

*Remarks*: *Armillaria xiaocaobaensis* published by Peng and Zhao in 2020 was clustered in a clade with *A. pungentisquamosa* on the *tef*1α gene tree, but it is not possible to determine whether they are the same species. On the one hand, the *tef*1α gene tree worldwide revealed that the *tef*1α sequence is powerless to distinguish closely related nascent species. The mechanism of balancing selection that occurred in the *tef*1α alleles could lead to situations where several species share the same evolutionary lineage, as was the case in *A. cepistipes* and *A. sinensis* (to be published). The IEL analysis in the study has demonstrated that two or more species of *Armillaria* shared a *tef*1α lineage and one species formed multiple *tef*1α lineages in the *tef*1α single-gene tree (Figure S6), and only 55% of the Chinese isolates of *Armillaria* could be differentiated as species by *tef*1α sequences (Table S1). Therefore, it is invalid procedurally to recognise the species of *Armillaria xiaocaobaensis* by only *tef*1α gene without a reproductive isolation criterion. On the other hand, *A. xiaocaobaensis* does not possess rhizomorphs and clamp connections, whereas *A. pungentisquamosa* has monopodial branching rhizomorphs in nature and a clamp connection at the base of the basidia and the basidioles.

*Armillaria pungentisquamosa* may be collected simultaneously with *A. mellea*, but can be easily distinguished from that species by the absence of a thick membranous annulus, meanwhile, the former had a pale brown pileus with dark spiky scales and later a pale honey colour and nearly denuded pileus. It may occur in the distribution range of *A. algida*, *A. bruneocystidia*, *A. tibetica*, and *A. violacea*, but usually *A. pungentisquamosa* fruits earlier than the other species, and can be differentiated by its pale brown basidiomata with dark spiky scales.

**Figure 10.**
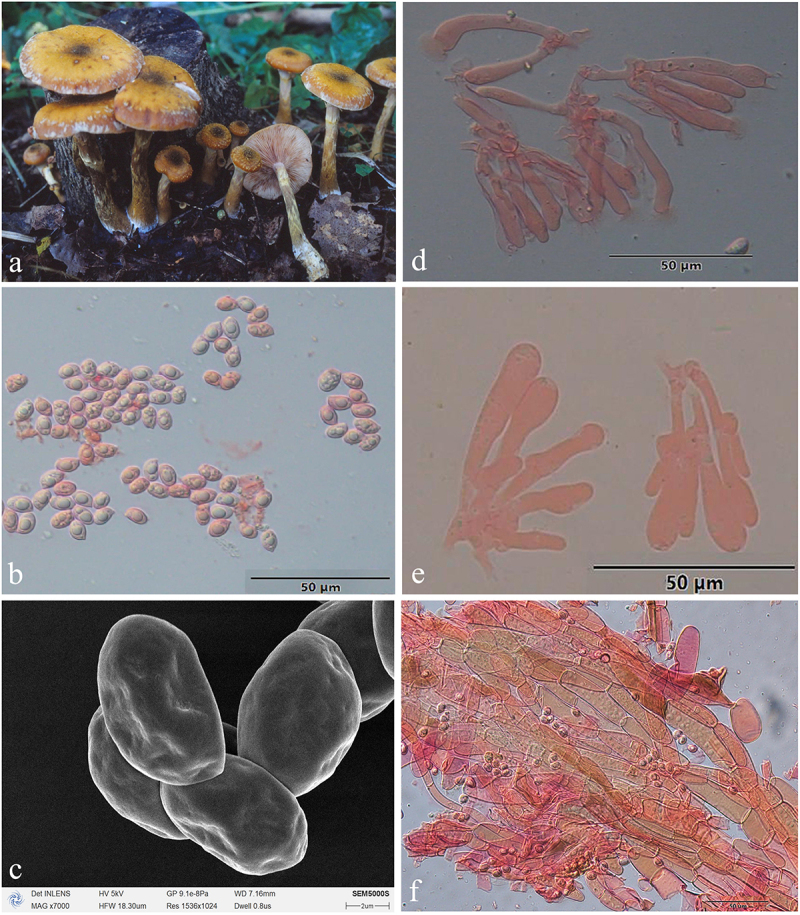
*Armillaria sinensis*. (a) Basidiomata (QZ96059). (b) Basidiospores. (c) Basidiospores under SEM. (d) Basidia and basidioles. (e) Cheilocystidia. (f) Hyphae of pileal squamules (QZ21016).

***Armillaria sinensis*** G.F. Qin, J. Zhao, H.C. Wang, Y. Yuan & Y.C. Dai, sp. nov. Figure 10

*MycoBank*: MB 831856.

Chinese Biological Species (CBS) F.

Chinese Phylogenetic Species (CPS) *Armillaria sinensis.*

*Etymology*: *Sinensis* (Lat.): refers to the species having a distribution over China.

*Holotype*: China, Jilin Province, Changbai Mts., Yuehualin, 42°03’ N, 128°03’ E, elev. 1,850 m, 8 September 2021, G.F. Qin (IPF QZ21016).

*Descriptions*: Basidiomata small to medium-sized, solitary to caespitose, or loosely grouped on rotten woody debris. Pilei 3–15 cm in diam., 0.4–2.5 cm thick, at first hemispherical or campanulate, then plano-convex to applanate, usually depressed; usually dry; light yellow (4A4), yellow (3B7), yellowish orange (4B7–4B8), olive yellow (3C7), greyish yellow (4C6), dark yellow (4C8), brownish orange (5C5–6C5), olive brown (4D8), light brown (5D7–5D8), brown (6E4–6E6) at the margin, yellowish orange (4B7–4B8), brownish yellow (5C7), olive brown (4D4–4E3–4E8), yellowish brown (5F5–5F6), dark brown (6F4–6F5–6F6) at the centre when young; pale yellow (4A3), light yellow (4A4), pale orange (5A3), greyish yellow (3C5–4B4–4B6), greyish orange (5B4–5B5), brownish orange (5C5–5C7–6C6), brownish yellow (5C7–5C8), light brown (5D5–6D5–5D7) at the margin, pale yellow (4A3), pale orange (5A3), greyish yellow (4B4–4B6), greyish orange (5B3–5B4), brownish orange (5C5), olive (3E4), light brown (5D4), yellowish brown (5E5), olive brown (4F5), yellowish brown (5F4–5F5–5F7–5F8), brown (6E4–6E5), dark brown (6F4–6F5), sometimes with ocella at the centre when mature. *Pileal squamules* fibrillose, brownish orange (5C6), olive (3F5), olive brown (4F6), yellowish brown (5F5–5F6), brown (6E6), dark brown (6F4–6F8), distributed over entire surface of cap but more concentrated and suberect towards the centre, more dispersed and flattened towards margin. Margin entire, usually incurved, and striate.

Lamellae decurrent, with striped ridges downwards to annulus, at first white (4A1), yellowish white (3A2–4A2), orange-white (5A2–6A2), pale yellow (4A3), pale orange (5A3–6A3), light yellow (4A4), light orange (5A4), greyish orange (5B3–5B4), brownish orange (6C6), light brown (5D4–5D5), brownish grey (5E2), subdistant, lamellulae present, margin smooth.

Stipe central, 4–11 cm long, 0.3–2 cm in diam. at the apex, clavate to bulbous at base, sometimes abruptly bulbous, occasionally cylindrical, longitudinally striate, white (4A1), yellowish white (4A2), orange-white (6A2), pale orange (5A3–6A3), greyish yellow (3B6), greyish orange (5B3–6B3), brownish orange (5C5–6C6), light brown (5D4–5D5–5D6) above annulus; the middle light yellow (4A4), greyish yellow (3B4–4B4–4B5), greyish orange (5B3–5B5–6B3), brownish orange (6C4), olive brown (4D5), light brown (5D4–5D5–6D4–6D5–6D6), yellowish brown (5E4–5E6–5E8), brown (6E4–6E5); the base greyish yellow (4B4–4B5–4C5), greyish orange (5B4), brownish orange (5C4), olive brown (4D5), light brown (5D4–6D5), brown (5E5–5E6–5E7–5E8–6E4–6E8), dark brown (5F4–5F6–6F6–7F6); densely covered with greenish yellow (1A7–1A8), light yellow (3A4–3A5), yellow (3A6), greyish yellow (4B6), grey (6B1), brown (6E6) flocculose veil remnants, resembling zigzag markings; sometimes the base densely covered with white or greyish green (1D7) mycelium.

Annulus submembranous, single, thick, sometimes double, thin, persistent; rarely cortinate, ephemeral; inner veil yellowish white (1A2–3A2), outer margin pale yellow (3A6–3A7–4A3), light yellow (1A5–3A3–3A4–4A4), greenish yellow (1A7–1A8), vivid yellow (3A8), greyish yellow (3B4–4B4–4B6), grey (6B1), brown (6E6), dark brown (6F5).

Rhizomorphs black, thick, monopodial branching in nature.

Basidia clavate, 10.0–60.0 × 4.0–12.5 μm, upper portion constricted or constriction inconspicuous, with a clamp connection at the base, four-sterigmate, rarely one or two-sterigmate, usually thin- to slightly thick-walled, yellowish green to brownish yellow. The pigments of basidia dissolve in KOH, sometimes brownish yellow crassobasidia (≤2 μm) are formed when lamellae develop abnormally. Sterigmata 3–7 μm (5 ± SD1.06).

Basidiospores ivory white in mass, ellipsoid, lacking a suprahilar depression, [332/20/14] (6.0)7.0–9.5(41.0) × (4.0)4.5–6.0(9.0) μm, [Q = (1.14)1.20–1.87(4.56), *Q*_*m*_ = 1.55 ± SD0.23]; thin- to thick-walled (≤1.1 μm), nearly colourless, brownish yellow, smooth, cyanophilous, inamyloid, with a prominent apiculus.

Cheilocystidia 9–53 × 4–13.5 μm, polymorphic, viz., fusiform, elliptical, ovoid, cylindrical, clavate, with a clamp connection at the base, usually with irregular papilla at the apex; nearly hyaline, thin-walled to slightly thick-walled (≤0.2 μm), usually forming a compact sterile lamella-edge that is easily broken and disappears.

Pleurocystidia absent.

Pileal squamules composed of more or less parallel, cylindrical, slightly thick- to thick-walled (≤1.1 μm) hyphae, clampless, grey to brownish yellow, 16–92 × 4.5–17 μm.

*Specimens examined*: China, Heilongjiang Province, Dailing Area, Yongcui Forestry Farm, on stump of *Pinus koraiensis*, 3 September 1999, Jun Zhao, Shu-Min Tian, QZ99105; on stump of *Ulmus*, 3 September 1999, Jun Zhao, Shu-Min Tian, QZ99107; Jilin Province, Antu County, Changbaishan Nature Reserve, on base of *Acer*, 11 September 1997, Guo-Fu Qin, Jun Zhao, QZ97033; on stump of *Betula platyphylla*, 9 September 1997, Guo-Fu Qin, Jun Zhao, QZ97018; on fallen trunk of *Tilia*, 9 September 1997, Guo-Fu Qin, Jun Zhao, QZ97014; on stump of *Tilia amurensis*, 14 September 1997, Guo-Fu Qin, Jun Zhao, QZ97045; on angiosperm wood debris, 30 August 1996, Guo-Fu Qin, QZ96026; on fallen angiosperm trunk and wood debris, 9 September 1997, Guo-Fu Qin, Jun Zhao, QZ97017; Liaoning Province, Huanren County, Laotuding Mts., on living tree of *Acer*, 11 September 1996, Guo-Fu Qin, Jun Zhao, Shu-Min Tian, QZ96058; on base of *Fraxinus mandshurica*, 11 September 1996, Guo-Fu Qin, Jun Zhao, Shu-Min Tian, QZ96053; on stump of *Fraxinus rhynchophylla*, 11 September 1996, Guo-Fu Qin, Jun Zhao, Shu-Min Tian, QZ96059; on base of *Pinus koraiensis*, 11 September 1996, Guo-Fu Qin, Jun Zhao, Shu-Min Tian, QZ96056; on stump of *Pinus koraiensis*, 11 September 1996, Guo-Fu Qin, Jun Zhao, Shu-Min Tian, QZ96052; on rotten wood of *Quercus mongolica*, 10 September 1996, Guo-Fu Qin, Jun Zhao, Shu-Min Tian, QZ96051; on stump of *Quercus mongolica*, 11 September 1996, Guo-Fu Qin, Jun Zhao, Shu-Min Tian, QZ96060; on rotten angiosperm wood, 11 September 1996, Guo-Fu Qin, Jun Zhao, Shu-Min Tian, QZ96054; Qingyuan County, Houtoushan, host unkown, 11 September 2019, Guo-Fu Qin, QZ19020; Shaanxi Province, Mei County, Honghegu Forest Farm, on rotten wood of *Tilia*, 18 September 2005, Guo-Fu Qin, Jun Zhao, Han-Chen Wang, QZ05030; on rotten angiosperm wood, 23 September 2005, Jun Zhao, Han-Chen Wang, QZ05047.

*Remarks*: *Armillaria sinensis* can be differentiated from *A. singula* by the morphological features of the basidiomata, as the first species has large golden yellow or light brown caps, fibrillose scales (composed of filamentous hyphae, 16–92 × 4.5–17 μm), yellow veil remnants on stipe, clavate to bulbous stipe base, a rarely cortinate ring, and the latter has small yellowish brown caps, fibrillose scales (hyphae short and wide, 32–65 × 10–20 μm), clavate stipe base, and a cortinate ring (Cha et al. 1994). The results of IGS1 sequence analysis further support that they are different species (Coetzee et al. 2015).

**Figure 11.**
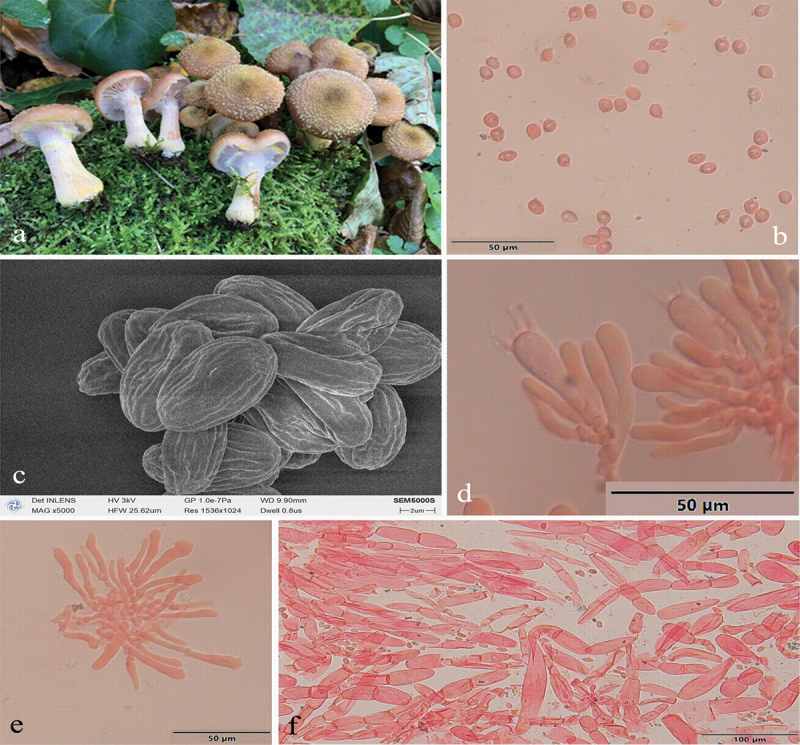
*Armillaria cepistipes*. (a) Basidiomata (QZ19042). (b) Basidiospores. (c) Basidiospores under SEM. (d) Basidia and basidioles. (e) Cheilocystidia. (f) Hyphae of pileal squamules (QZ99134).

Guo et al. (2016) named CBS F as *Armillaria cepistipes* based only on the high similarity *tef*1α gene sequences between CBS F and *A. cepistipes*, regardless of the fact that they are two distinct biological species. Here, we reject such a proposal for the following reasons. Firstly, the intersterility is confirmed with 61 paired combinations of mating tests (eight European strains of *A. cepistipes* and at least eight haploid isolates of *A. sinensis*); among them, 59 pairs showed an obvious antagonistic black line and two pairs did not (Table 3). Secondly, *Armillaria cepistipes* and *A. sinensis* were two distinct phylogenetic species on the Bayesian concatenated multigene species tree. The two species were located in the same *tef*1α lineage is caused by the fact a balancing selection mechanism occurred in the *tef*1α gene (to be published). Thirdly, they have different macromorphological characteristics, i.e. *A. sinensis* has a golden yellow to light brown cap, double or single annulus, hairy scales (Figure 10), whereas the cap of *A. cepistipes* is dark brown (Termorshuizen and Arnolds 1987), ochraceous to ochraceous brown (Antonín et al. 2009) or yellowish brown to red brown to dark brown (Ota et al. 2009), single and cortinate annulus, fibrillose scales (Figure 11). Fourthly, they possessed distinct basidiospore morphology, the spores of *A. sinensis* is ellipsoid, larger [(7.0)8.0–10.0(11.0) × (4.0)5.0–6.0(6.5) μm, Q = (1.33) 1.40–(1.52)–1.67(1.73), *Q*_*m*_ ≥1.55], surface completely smooth under both the light and electron microscope (Figure 10); whereas that of *A. cepistipes* is pruniform (Romagnesi and Marxmüller 1983) or broadly ellipsoid to subglobose, smaller [7.0–9.0(10) × 4.5–6.5(7.0) μm, Q = (1.7–(1.56)–1.8] (<8.4 × 5–6 μm, *Q*_*m*_ < 1.5) (Romagnesi and Marxmüller 1983), surface smooth under the light microscope but with small longitudinal ridges under the electron microscope (Figure 11). To date, *A. cepistipes* is the only species with basidiospore of small longitudinal ridges in the “Gallica cluster”.

*Armillaria sinensis* is distributed over most of China, from the cold temperate to the subtropical zone. *A. sinensis* seems to be a psychrophilic species because it has the highest latitude distribution (50.5° N) and the latest fruiting times nearing the snow season in northeastern China, whereas in southern China it is found at higher elevations. This is similar to the distribution pattern of *A. cepistipes* in Europe (Guillaumin et al. 1993; Keča and Solheim 2011). In Japan, however, *A. cepistipes* is apparently distributed over the warm temperate to the temperate zone (Hasegawa et al. 2011).

**Figure 12.**
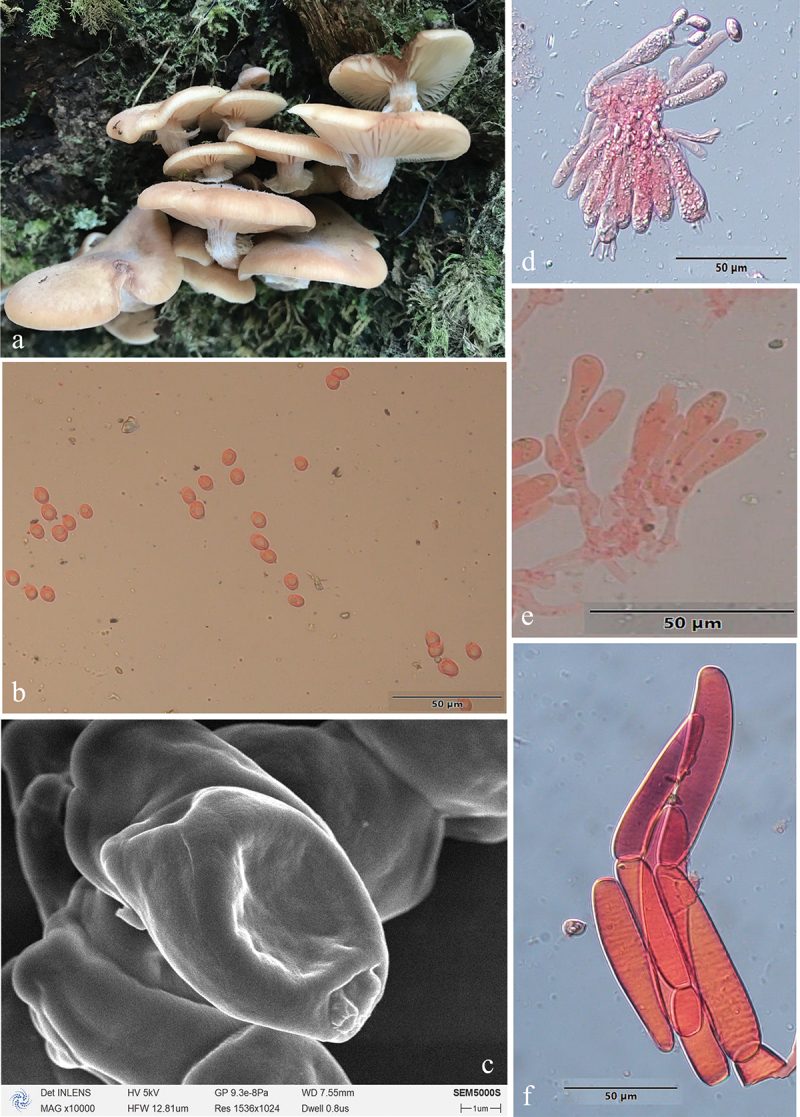
*Armillaria tibetica*. (a) Basidiomata (QZ19097). (b) Basidiospores. (c) Basidiospores under SEM. (d) Basidia and basidiospores. (e) Cheilocystidia. (f) Hyphae of pileal squamules (QZ19097).

***Armillaria tibetica*** G.F. Qin, H.C. Wang, J. Zhao, Y. Yuan & Y.C. Dai, sp. nov. Figure 12

*MycoBank*: MB 831861.

Chinese Biological Species (CBS) J.

*Etymology*: *tibetica* (Lat.): refers to the species being distributed mainly in the Xizang's Plateau.

Chinese Phylogenetic Species (CPS) *Armillaria tibetica*.

*Holotype*: China, Hubei Province, Wufeng County, Houhe Nature Reserve, Yangzixi, 30°08” N, 110°57” E, elev. 1,700 m, 21 October 2019, G.F. Qin (IPF QZ19097).

*Descriptions*: Basidiomata small to large-sized, solitary or loosely grouped or caespitose at the base of living trees, fallen trunks, and rotten wood. Pilei 2.5–13 cm in diam., 0.4–2.7 cm thick, at first hemispherical-convex to convex, sometimes with mammilla, then plano-convex, applanate, depressed or undulate, sometimes with umbo; usually dry, sometimes hygrophanous; greyish orange (6B4), brownish orange (7C6), light brown (7D6), brown (7E6) at the margin, light brown (6D5–7D5–7D6), greyish brown (5E3), dark brown (7F6) at the centre when young; light orange (5A4), greyish orange (6B6), greyish red (7B5), brownish orange (7C5–7C6), light brown (6D6–7D5–7D6), brown (6E8–7E6–7E8), reddish brown (8D5–8D6–8E5–8E6), violet-brown (10E6) at the margin, pale yellow (4A3), pale orange (5A3), light orange (5A4–6A4), greyish orange (6B4), brownish orange (7C5–7C6), light brown (7D5–7D7), reddish brown (8E5–8E6), dark brown (7F6–8F5), violet-brown (10E6) at the centre when mature. Pileal scales fine-velvety, abundant, white or nearly white, well-distributed over entire surface of cap, generally not concentrated towards the centre, disappearing when mature or after rain. Margin entire, usually slightly incurved, strongly striate, sometimes with white velar remnants.

Lamellae adnate with decurrent tooth or sinuate, striped ridges downwards to annulus, white, yellowish white (4A2), orange-white (5A2–6A2–6A3) when young, orange-white (4A3), pale orange (6A3), greyish orange (6B4), brownish orange (7C6), light brown (7D5), brown (6E6–7E6) when mature, subdistant, lamellulae present, margin smooth.

Stipe central, 2.5–9.8 cm long, 0.3–2.5 cm in diam. at the apex, cylindrical, clavate or broadened at base, longitudinally striate, orange-white (5A2–6A2), pale orange (6A3), light orange (6A4), greyish orange (5B4–6B3), greyish red (7B4), brownish orange (6C5–7C4–7C5), light brown (7D5–7D6), brown (7E7), reddish brown (8D5–8E6) above the annulus; the middle portion orange-white (6B2), pale orange (6A3), light orange (6A4), greyish orange (6B4), brownish orange (6C3, 7C5), light brown (6D5–7D5–7D6), reddish brown (8D5–8E6), violet-brown (10F4); the base greyish orange (6B3–6B4), brownish orange (6C4–7C5), light brown (6D4–6D5–7D4–7D5–7D6), brown (7E7), dark brown (6F5, 8F6), reddish brown (8E4–8E5–8E6), violet-brown (10F4); covered with white silky fibrillose velar remnants, sometimes resembling zigzag markings, soon disappearing when touched.

Annulus arachnoid, thin, ephemeral, white, or nearly white.

Basidia clavate, 25–48 × 7–10 μm, upper portion constricted or constriction inconspicuous, with a clamp connection at the base, four-sterigmate, usually thin- to slightly thick-walled, yellowish green to brownish yellow. The pigments of the basidia disappear gradually in KOH, sometimes brownish yellow crassobasidia (≤1.5 μm thick) are formed when the lamellae develop abnormally. Sterigmate 2.0–6.0 μm.

Basidiospores ivory white in mass, ellipsoid, lacking a suprahilar depression, [657/22/20] (6.8)7.5–9.5(11.5) × (4.2)5.0–7.0(7.8) μm, [Q = (1.04)1.16–1.80(2.08), *Q*_*m*_ = 1.45 ± SD0.16]; thin- to thick-walled (≤1 μm), hyaline, greenish blue to brownish yellow, smooth, cyanophilous, inamyloid, with a prominent apiculus.

Cheilocystidia 10.5–49 × 4–17 μm, polymorphic, viz., clavate, fusiform, cylindrical, elliptical, ovoid, with a clamp connection at the base, with papilla or irregular branched papilla at the apex; thin- to slightly thick-walled (≤0.5 μm), nearly hyaline, greenish blue to brownish yellow, forming a compact sterile lamella-edge that is easily broken and disappears.

Pleurocystidia absent.

Pileal squamules composed of parallel, cylindrical, slightly thick- to thick-walled (≤1.5 μm) hyphae, simple septate, greenish blue, grey to brownish yellow, 14–110 × 5–22 μm.

*Specimens examined*: China, Hubei Province, Wufeng County, Houhe Nature Reserve, Yangzixi, on ground of mixed forest, 16 October 2019, Guo-Fu Qin, QZ19096; on living trunks of *Cerasus pseudocerasus*, 16 October 2019, Guo-Fu Qin, QZ19097; Shaanxi Province, Mei County, Taibai Mts., on living tree of *Betula albo-sinensis*, 7 August 2004, Yu-Cheng Dai, QZ04017, QZ04018; Shanxi Province, Jiaocheng County, Pangquangou Nature Reserve, on rotten wood of *Picea*, 23 September 2006, Han-Chen Wang, QZ06003; Sichuan Province, Emei Mts., Jieyindian, on base of living *Acer* sp. 16 October 2019, Guo-Fu Qin, QZ19076; Leidongping, on dead trunk of *Betula* sp., 16 October 2019, Guo-Fu Qin, QZ19077; Xinjiang Autonomous Region, Xinyuan County, Nalati Nature Reserve, on stump of *Betula pensula*, 23 September 2002, Jun Zhao, QZ02044, QZ02045, QZ02046, QZ02047, QZ02048; on stump of *Picea schrenkiana*, 23 September 2002, Jun Zhao, QZ02049, QZ02050, QZ02051; Xizang Autonomous Region, Bayi County, Nyingchi, on rotten wood of *Picea*, 30 July 2004, Yu-Cheng Dai, QZ04001, QZ04002; Gongbujiangda County, Nyingchi, on fallen trunk of *Abies*, 31 July 2004, Yu-Cheng Dai, QZ04008; on stump of *Abies*, 30 July 2004, Chang-Jun Yu, Han-Chen Wang, QZ04032; Yunnan Province, Naxi County, Yulong Snow Mts., on rotten wood of *Betula*, 14 September 2000, Jun Zhao, Shu-Min Tian, QZ00101, QZ00102, QZ00104; on rotten wood of *Picea likiangensis*, 14 September 2000, Jun Zhao, Shu-Min Tian, QZ00103, QZ00108; on rotten angiosperm wood, 14 September 2000, Jun Zhao, Shu-Min Tian, QZ00105, QZ00106, QZ00107, QZ00110, QZ00112; Shangri-La County, Shuodu Lake, on rotten wood of *Abies ferreana*, 17 September 2000, Jun Zhao, Shu-Min Tian, QZ00115, QZ00116, QZ00117, QZ00118, QZ00119, QZ00121; Xilianpen Mts. Pass, on rotten wood of *Quercus pannosa*, 17 September 2000, Jun Zhao, Shu-Min Tian, QZ00122.

*Remarks*: The CBS J had once been named *Armillaria qinii* by H.C. Wang in his doctoral thesis (Wang 2007). We submitted a large number of coding sequences to GenBank under the *A. qinii* in the past few years. However, the biological species is officially published by Qin, the be-named person himself, so it would not be appropriate to continue using *A. qinii*. In the present work, we re-specify the holotype QZ19097 and abandon Wang’s holotype QZ02044.

*Armillaria tibetica* is the most common *Armillaria* species in western China and is distributed over nine provinces; it is especially dominant in the Qinghai-Xizang Plateau. It also occurs in the same locations as *A. sinensis* but can be differentiated by morphological and microscopic characteristics. *A. sinensis* has golden yellow to light brown basidiomata, a double ring, yellow flocculose scales and veil remnants, and smaller and longer spores 8.0–10.0 × 5.0–6.0 μm, *Q*_*m*_ = 1.56.

It also occurs in the realm of *Armillaria borealis* but can be easily distinguished by morphological characteristics. *Armillaria borealis* has a reddish to purple brown pileus, brown to dark brown spiky scales, thick membranous persistent annulus, usually with merodont scales and tomentose veil remnants on the stipe.

**Figure 13.**
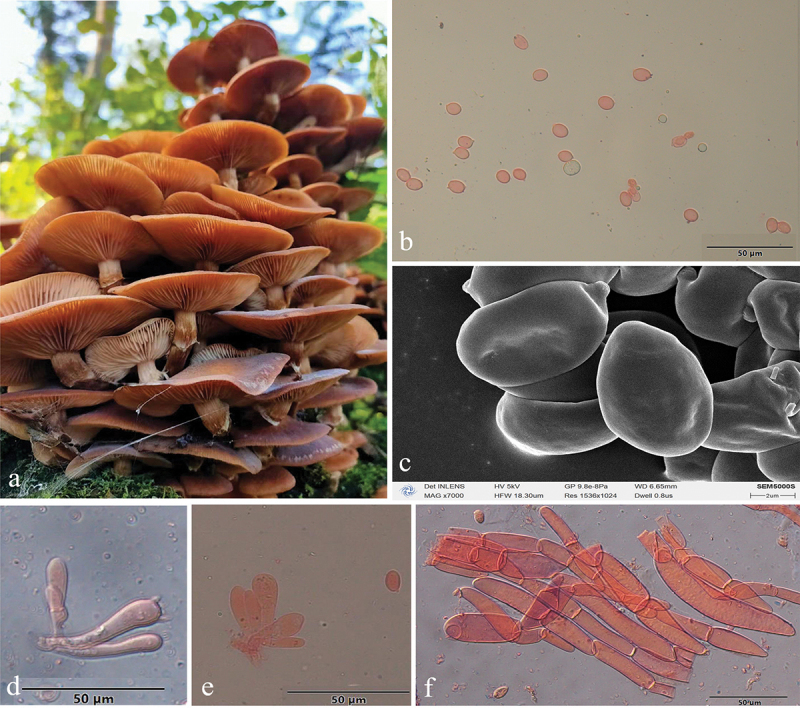
*Armillaria violacea*. (a) Basidiomata (QZ19109). (b) Basidiospores. (c) Basidiospores under SEM. (d) Basidioles and a basidium. (e) Cheilocystidia. (f) Hyphae of pileal squamules (QZ19109).

***Armillaria violacea*** H.C. Wang, W.M. Qin, G.F. Qin, J. Zhao & Y.C. Dai, sp. nov. Figure 13

*MycoBank*: MB 831859.

Chinese Biological Species (CBS) N.

Chinese Phylogenetic Species (CPS) *Armillaria violacea*.

*Etymology*: *violacea* (Lat.): refers to the purple colour of the basidomata.

*Holotype*: China, Sichuan Province, Meishan, Emei Mountain, 29°34’ N, 103°21’ E, elev. 1,800 m, 29 October 2019, G.F. Qin (IPF QZ19109).

*Descriptions*: Basidiomata small to large-sized, solitary or caespitose, concrescent at the base. Pilei 1.7–10 cm in diam., at first hemispherical or convex, usually with mammillae, then plano-convex to applanate or slightly depressed, sometimes umbonate; usually dry; greyish orange (6B3), brownish orange (6C6–7C6), light brown (7D6), reddish brown (8D5) at the margin, brownish orange (7C6), greyish brown (6D3–6E3), light brown (6D6), dark brown (6F6, 8E6) at the centre when young; greyish orange (5B4–5B5), brownish orange (6C4), greyish brown (6D3), light brown (7D6), brown (7E4), greyish ruby (12C4), brown (7E7) at the margin, greyish orange (5B4–5B5), greyish rose (12B3), brownish orange (6C6), greyish red (8C4), greyish brown (6E3), brown (7E7), dark brown (7F4), greyish magenta (14D5–14D8), dark purple (14E5–14E8) at the centre when mature. Pileal scales fine-velvety or flocculose, abundant, white or nearly white, usually distributed over entire surface but more concentrated towards the centre, disappearing when mature or after rain. Margin straight and entire, usually incurved when young, striate when mature.

Lamellae adnate or adnate with decurrent tooth, with striped ridges downwards to annulus, white or reddish white (7A2), pale orange (6A3) when young, pale red (7A3), greyish red (7B3–7B6), brownish orange (6C5, 7C3–7C6), light brown (7D4–7D6–7D7), dull red (9C3) when mature, subdistant, lamellulae present, margin smooth.

Stipe central, 3.5–12 cm long, 0.3–1.5 cm in diam. at the apex, cylindrical or broadened at base, sometimes slightly bulbous; white to orange-white (6A2) above the annulus, the middle portion greyish orange (6B3) to brownish orange (6C3), base light brown (6D4) when young, greyish yellow (4B4), greyish orange (6B3–6B4), light brown (5D4), greyish red (7B3) above the annulus, middle portion greyish yellow (4B4), pale orange (6A3), brownish orange (6C4), light brown (6D4–6D5), brownish grey (7E2), dull red (9C3), base light brown (6D4–7D4), reddish brown (9D3), dark brown (6F4–6F5–6F6), covered with white or nearly white fibrillose veil remnants, sometimes resembling snakeskin markings, soon disappearing when touched.

Annulus cortinate, single, thin and delicate, ephemeral, white or nearly white.

Basidia clavate, 26–50 × 7–10 μm, four-sterigmate, usually upper portion constricted or constriction inconspicuous, with a clamp connection at the base, thin- to slightly thick-walled, yellowish green to brownish yellow. The pigments disappear gradually in KOH, sometimes brownish yellow crassobasidia (≤1.5 μm) are formed, and crassobasidia with septa are formed when lamellae develop abnormally.

Basidiospores ivory white in mass, elongate, lacking a suprahilar depression, [210/7/7] (7.0)7.5–9.8(10.0) × (4.3)4.7–6.0(6.0) μm, [Q = (1.29)1.38–2.00(2.08), *Q*_*m*_ = 1.64 ± SD0.14]; thin- to slightly thick-walled (≤0.8 μm), nearly hyaline, greenish blue to brownish yellow, smooth, cyanophilous, inamyloid, with a prominent apiculus.

Cheilocystidia 12–44 × 6–11 μm, polymorphic, viz., clavate, fusiform, cylindrical, elliptical, ovoid, with a clamp connection at the base, sometimes with papilla or irregular branched papilla at the apex; slightly thick- to thick-walled (≤1.0 μm), nearly hyaline, usually forming a compact sterile lamella edge but easily broken and disappearing.

Pleurocystidia absent.

Pileal squamules composed of cylindrical, slightly thick- to thick-walled (≤1.2 μm) hyphae, sometimes thicken in the form of waves, simple septate, pale brown to brown, 15–145 × 4.5–20 μm.

*Specimens examined*: China, Hubei Province, Wufeng County, Houhe Nature Reserve, on fallen trunk of *Cunninghamia lanceolata*, 21 October 2019, Guo-Fu Qin, QZ19098, QZ19099; on living tree or fallen angiosperm trunk, 31 October to 5 November 2019, Guo-Fu Qin, QZ19122, QZ19123, QZ19132; Shaanxi Province, Mei County, Taibai Mts., on living tree of *Betula*, 17 September 2005, Guo-Fu Qin, Jun Zhao, Han-Chen Wang, QZ05014; on dead trunk of *Magnolia liliflora*, 17 September 2005, Guo-Fu Qin, Jun Zhao, Han-Chen Wang, QZ05015; on living angiosperm root, 17 September 2005, Guo-Fu Qin, Jun Zhao, Han-Chen Wang, QZ05017; Sichuan Province, Meishan city, Emei Mountain, on rotten wood of *Castanopsis platyacantha*, 19 October 2002, Yu-Cheng Dai, QZ02065, QZ02066, QZ02067, QZ02068, QZ02069, QZ02070; on rotten angiosperm stump, 29 October 2019, Guo-Fu Qin, QZ19107, QZ19108, QZ19109, QZ19110.

*Remarks*: *Armillaria violacea* is probably one of the most psychrophilic of the *Armillaria* species in western China; all the basidiomata occurred in late October and above an elevation of 2,000 m. It is also a member of the “Gallica cluster” (Korhonen 1995) or “Gallica superclade” (Klopfenstein et al. 2017) and is sympatric with *A. algida*, *A. borealis*, *A. pungentisquamosa*, *A. sinensis*, and *A. tibetica*, but can be distinguished from all these species by its distinctive purple brown basidiomata, cortinate annulus, white silky fibrillose scales and veil remnants, and elongate spores (*Q*_*m*_ = 1.64).

## 4. Discussion

### 4.1. *The concepts of the biological species, phylogenetic species, and taxonomic species of* Armillaria *have been unified*

We have identified 16 Chinese biological species (CBS) of *Armillaria* on the basis of reproductive isolation, and recognised 15 Chinese phylogenetic species (CPS) in the light of a concatenated six-gene phylogenetic analysis. With the exception of homothallic CBS G and heterothallic *A. mellea*, all the CBS and CPS were identical and possessed the same species boundaries. According to the CBS, CPS, and the results of macro and micromorphological investigation, eight new species of *Armillaria* in China were described. Thus, an integration concepts of the biological species, phylogenetic species, and morphological species in the identification of *Armillaria* in China has been achieved. Among these three approaches, in theory, the criterion of biological species (BS) plays a key role, because biological species constitutes the basis of genetic differentiation across the domains of life. All cellular and non-cellular life forms, be it a virus that does not rely on sexual reproduction or a bacterium that cannot be cultivated, can be identified as biological species through a genomic approach based on homologous recombination (Meyer et al. 2016; Bobay and Ochman 2017, 2018). In other words, reproductive isolation guarantees the independence of phylogenetic species (Hudson and Coyne 2002). In practice, however, our results demonstrated that the methods of BS and PS are equally important and both made up for the other’s shortcomings. On one hand, because there are high levels of interfertility between *A. algida* and *A. luteopileata*, the BS method cannot determine whether they are two species or two geographically separated populations within a species. The GCPSR clearly revealed that they are two distinct species, because both of them formed their own phylogenetic species on the six-gene species tree and IEL on the single-gene tree. Therefore, the intersterility threshold between nascent species with gene flow cannot be determined without GCPSR. Furthermore, with regard to the identification of a certain isolate, GCPSR avoided the subjective mistake of judgement in the mating test. Haploid QZ96027 has been identified as *A. gallica* for more than 20 years, however, it falls into the phylogenetic species, *A. luteopileata*, both on the six-gene species tree and on IEL. For this reason, we conducted the mating test repeatedly and finally confirmed that this isolate is *A. luteopileata*. On the other hand, the biological species becomes a standard for determining whether a lineage is independent and speciated. Without a mating test reference, any statistically supported clade on a single gene tree can be regarded as a species regardless of the evolution mechanism of the gene. Based on these results, it is recommended that the results of the mating test and GCPSR must be simultaneously submitted when publishing a new heterothallic species in the future.

### 4.2. *The establishment of GCPSR plays an important role in the taxonomy of* Armillaria

Samples from dry specimens, infected trees, or rhizomorphs can be identified, for example, those diploid isolates of *Armillaria* from Bhutan can be easily identified (Coetzee et al. 2005). Meanwhile, classical morphological taxonomy can be connected with modern biological and phylogenetic species taxonomy. With the GCPSR tool, those age-old types in the museums can be sequenced and identified as modern species. Finally, Sequence-Based Classification and Identification (SBCI) is more and more widely used in taxonomy, ecology, and evolutionary biology of fungi (Hibbett et al. 2016; De Beer et al. 2016); the sequences recovered from environmental samples were proposed as types for fungi (Hawkworth et al. 2016). The GCPSR approach provides a set of community standards for identifying *Armillaria* sequences from environments.

### 4.3. *The GCPSR tool could be employed to recognize* Armillaria *worldwide*

To date, 16 biological species of *Armillaria* are found in China, where the species diversity is much higher than in other continents or regions in the world. This phenomenon is consistent with other wood-decaying fungi (Wu et al. 2022a, 2022b; Yuan et al. 2023; Zhao et al. 2024). In terms of geographical distribution, the highest species diversity is observed in the temperate zone such as Hubei and Sichuan Prov., followed by the cold temperate and alpine zone such as Northern China and Xizang, etc., and the subtropical and tropical zones such as Gaungdong, Fujian and Hainan Provinces have less *Armillaria* species. This coincided with the global distribution patterns of Agaricomycetes (Varga et al. 2019). Many Chinese species, especially those belonging to the “Gallica cluster”, possessed a shorter evolutionary history, even partial compatible mating reaction and partial reproductive isolation were observed in three ancient species (*A. algida*, *A. bruneocystidia*, and *A. luteopileata*). Therefore, the genetic distance between most species in China is much smaller than that between species in other continents. Since the GCPSR approach can distinguished Chinese species of *Armillaria*, it may also discriminate the species of *Armillaria* in other continents or regions.

## Supplementary Material

Table_S1_Clean.docx
