# Supplementary material for: Phylogeny and species diversity of Armillaria in China based on morphological, mating test, and GCPSR criteria
Source: Mycology. 2024 Nov 13;16(2):777–811. doi: 10.1080/21501203.2024.2404121 (PMC12096668; doi:10.1080/21501203.2024.2404121)
Supplement: Table_S1_Clean.docx [file TMYC_A_2404121_SM7496.docx]

**Table S1.** Specimen or isolate information of *Armillaria* and *Desarmillaria* and GenBank accession number used in this study.

| **Species** | **Specimen no.** | **Date** | **CBS** | **Locality** | **Host and habitat** | **Collector(s)** | ***Actin*** | ***H3h*** | ***Hisps*** | **LSU rDNA** | ***Rpb*1** | ***Tef*1*α*** |
| --- | --- | --- | --- | --- | --- | --- | --- | --- | --- | --- | --- | --- |
| *A. algida* | QZ02072_1 | 2002/10/19 | O | 29º33' N, 103º21' E. Emei Mountain, Sichuan Prov. | *Castanopsis sempervirens.* Dead wood. Elevation 1400m. | Y.C. Dai | MN463993 | PP444018 | PP443882 | MN170668 | PP443145 | MN463858 |
| *A. algida* | W16032_21 | 2016/10/28 | O | 31º00' N, 111º10' E. Xiaobaoping, Yichang, Hubei Prov. | *Quercus* sp. Rotten wood. Elevation 1200m. | J.J. Deng | MN463995 | PP444043 | MT673902 | MN170670 | MT674049 | MN463860 |
| *A. algida* | QZ19080_1 | 2019/10/21 | O | 30º04' N, 110º32' E. Yangzixi, Houhe Nature Reserve, Wufeng, Hubei Prov. | Caespitose on rotten fallen wood. Elevation 1100m. | G.F. Qin | MT647022 | PP444019 | MT673880 | MT644742 | MT674031 | MT674170 |
| *A. algida* | QZ19081_1 | 2019/10/21 | O | 30º04' N, 110º32' E. Yangzixi, Houhe Nature Reserve, Wufeng, Hubei Prov. | Caespitose on rotten wood. Elevation 1100m. | G.F. Qin | MT647023 | PP444020 | MT673881 | MT644743 | MT674032 | MT674171 |
| *A. algida* | QZ19085_6 | 2019/10/21 | O | 30º04' N, 110º32' E. Yangzixi, Houhe Nature Reserve, Wufeng, Hubei Prov. | Caespitose on fallen *Acer* sp. Elevation 1650m. | G.F. Qin | MT647024 | PP444021 | MT673882 | MT644744 | MT674033 | MT674172 |
| *A. algida* | QZ19087_3 | 2019/10/21 | O | 30º04' N, 110º32' E. Yangzixi, Houhe Nature Reserve, Wufeng, Hubei Prov. | Solitary or caespitose on stump of *Pyrus* sp. Elevation 1700m. | G.F. Qin | MT647025 | PP444022 | MT673883 | MT644745 | PP443146 | MT674173 |
| *A. algida* | QZ19088_2 | 2019/10/21 | O | 30º04' N, 110º32' E. Yangzixi, Houhe Nature Reserve, Wufeng, Hubei Prov. | Solitary or caespitose on fallen wood of *Pyrus* sp. Elevation 1700m. | G.F. Qin | MT647026 | PP444023 | MT673884 | MT644746 | MT674034 | MT674174 |
| *A. algida* | QZ19091_2 | 2019/10/21 | O | 30º04' N, 110º32' E. Yangzixi, Houhe Nature Reserve, Wufeng, Hubei Prov. | Caespitose on fallen angiosperm wood. Elevation 1700m. | G.F. Qin | MT647027 | PP444024 | MT673885 | MT644747 | MT674035 | MT674175 |
| *A. algida* | QZ19093_7 | 2019/10/21 | O | 30º04' N, 110º32' E. Yangzixi, Houhe Nature Reserve, Wufeng, Hubei Prov. | Caespitose on rotten angiosperm stump. Elevation 1700m. | G.F. Qin | MT647029 | PP444026 | MT673887 | MT644749 | MT674037 | MT674177 |
| *A. algida* | QZ19095_7 | 2019/10/21 | O | 30º04' N, 110º32' E. Yangzixi, Houhe Nature Reserve, Wufeng, Hubei Prov. | Caespitose on angiosperm stump. Elevation 1700m. | G.F. Qin | MT647030 | PP444027 | MT673888 | MT644750 | MT674038 | MT674178 |
| *A. algida* | QZ19100_2 | 2019/10/21 | O | 30º04' N, 110º32' E. Yangzixi, Houhe Nature Reserve, Wufeng, Hubei Prov. | Caespitose on stump of *Pyrus* sp. Elevation 1700m. | G.F. Qin | MT647031 | PP444028 | MT673889 | MT644751 | MT674039 | MT674179 |
| *A. algida* | QZ19101_5 | 2019/10/21 | O | 30º04' N, 110º32' E. Yangzixi, Houhe Nature Reserve, Wufeng, Hubei Prov. | Caespitose on stump of *Pyrus* sp. Elevation 1700m. | G.F. Qin | MT647032 | PP444029 | MT673890 | MT644752 | PP443147 | MT674180 |
| *A. algida* | QZ19102 | 2019/10/21 | O | 30º04' N, 110º32' E. Yangzixi, Houhe Nature Reserve, Wufeng, Hubei Prov. | Caespitose on angiosperm stump. Elevation 1700m. | G.F. Qin | MT647033 | PP444030 | MT673891 | MT644753 | MT674040 | MT674181 |
| *A. algida* | QZ19103_4 | 2019/10/21 | O | 30º04' N, 110º32' E. Yangzixi, Houhe Nature Reserve, Wufeng, Hubei Prov. | Caespitose on stump of *Morus alba*. Elevation 1700m. | G.F. Qin | MT647034 | PP444031 | MT673892 | MT644754 | MT674041 | MT674182 |
| *A. algida* | QZ19106_3 | 2019/10/29 | O | 29 º.58'N. 103º.37'E. Zhanglaoping, Emei Mountain, Sichuan Prov. | Solitary or caespitose on standing dead angiosperm. Elevation 1700m. | G.F. Qin | MT647035 | PP444032 | MT673893 | MT644755 | MT674042 | MT674183 |
| *A. algida* | Dai26847_1 | 2023/10/25 | O | 29º.71'N.95º.58'E. Medog Nyingchi, Xizang Autonomous Region | *Abies* sp. | Y.C. Dai | PP480530 | PP444017 | PP443710 | PP356102 | PP443144 | PP443422 |
| *A. algida* | Dai26847_2 | 2023/10/25 | O | 29º.71'N.95º.58'E. Medog Nyingchi, Xizang Autonomous Region | *Abies* sp. | Y.C. Dai | PP480531 |  | PP443711 | ^PP356103^ |  | PP443423 |
| *A. amygdalispora* | QZ00125_3 | 2000/9/17 | L | 28º02' N, 107º01' E. Kuankuoshui, Suiyang, Guizhou Prov. | Imbricate and caespitose on dead stump of *Cyclobalanopsis multinevis*. Elevation 1550m. | J. Zhao & S.M. Tian | MN463966 | PP444047 |  | MN170655 |  | MN463829 |
| *A. amygdalispora* | QZ00126_5 | 2000/9/17 | L | 28º02' N, 107º01' E. Kuankuoshui, Suiyang, Guizhou Prov. | *Cyclobalanopsis multinevis*. Solitary to imbricate and caespitose on dead stump. Elevation 1550m. | J. Zhao & S.M. Tian | MN463967 | PP444048 |  | MN170656 |  | MN463830 |
| *A. amygdalispora* | QZ08202 | 2002/10/20 |  | 30º57' N, 103º29' E. Qingcheng Mountain, Sichuan Prov. | Unknown | Y.C. Dai | MN463968 |  |  | MN170626 |  | MN463831 |
| *A. amygdalispora* | W17003 | 2017/9/25 | L | 31º06' N, 111º04' E. Liziping, Yichang, Hubei Prov. | *Quercus* sp. Dead stump. Elevation 969m. | Y.Y. Wang & CH. Zeng | MN463969 | PP444049 | MT673903 | MN170688 | MT674050 | MN463832 |
| *A. amygdalispora* | W17016 | 2017/9/28 | L | 29º40' N, 109º30' E. Laifeng, Hubei Prov. | *Quercus* sp. Dead stump. Elevation 712m. | Y.Y. Wang, X. Xie & C. Mu | MN463970 | PP444050 | MT673904 | MN170690 | PP443155 | MN463833 |
| *A. amygdalispora* | W17017 | 2017/9/28 | L | 29º22' N, 109º18' E. Lushui, Laifeng, Enshi, Hubei Prov. | *Quercus* sp. Dead stump. Elevation 712m. | Y.Y. Wang, X. Xie & C. Mu | MN463971 | PP444051 | MT673905 | MN170691 | MT674051 | MN463834 |
| *A. amygdalispora* | W17027 | 2017/9/30 | L | 30º12' N, 110º41' E. Wufeng, Yichang, Hubei Prov. | *Quercus* sp. Dead stump. Elevation 880m. | C.H. Wang | MN463972 | PP444052 | MT673906 | MN170692 | MT674052 | MN463835 |
| *A. amygdalispora* | W17039 | 2017/9/30 | L | 30º14' N, 109º00' E. Yuanbao, Lichuan, Hubei Prov. | *Quercus* sp. Dead stump. Elevation 1389m. | Y.Y. Wang & J. Yang | MT647045 | PP444053 | MT673907 | MT644765 | MT674053 | MN463836 |
| *A. borealis* | QZ01019 | 2001/9/3 | M | 31º30' N, 110º20' E. Jiuhuping, Shennongjia Mountain, Hubei Prov. | *Fagus* sp. Caespitose on dead trunk. Elevation 1600m. | J. Zhao | MT647047 |  | MT673911 | MT644767 | MT674057 | MT674194 |
| *A. borealis* | QZ04016_3 | 2004/8/6 | M | 34º00' N, 107º05' E. Xiabansi, Taibai Mountain, Shaanxi Prov. | Rotten wood of *Betula albo-sinensis*. Elevation 2850m. | Y.C. Dai | PP442702 |  | PP443885 |  | PP443168 | PP443439 |
| *A. borealis* | QZ05011_4 | 2005/9/17 | M | 34º07' N, 107º53' E. Taibai Mountain, Shaanxi Prov. | *Betula albo-sinensis*. Rotten fallen wood. Elevation 1800m. | G.F. Qin, J. Zhao & H.C. Wang | MN463976 |  | MT673913 | MN170659 | MT674059 | MN463840 |
| *A. borealis* | QZ05020_1 | 2005/9/18 | M | 34º06' N, 107º44' E. Honghegu, Taibai Mountain, Shaanxi Prov. | Rotten fallen trunk of *Betula albo-sinensis*. Elevation 2500m. | G.F. Qin, J. Zhao & H.C. Wang | MN463977 | PP444071 | MT673914 | MN170683 | MT674060 | MN463841 |
| *A. borealis* | QZ05021_1 | 2005/9/18 | M | 34º06' N, 107º44' E. Honghegu, Taibai Mountain, Shaanxi Prov. | *Betula albo-sinensis*. Rotten fallen trunk. Elevation 2500m. | G.F. Qin, J. Zhao & H.C. Wang | MN463978 | PP444072 | MT673915 | MN170682 | MT674061 | MN463842 |
| *A. borealis* | QZ06001 | 2006/9/18 | M | 35º25' N, 111º58' E. Lishan Nature Reserve, Qinshui, Shanxi Prov. | Mixed forest. Rotten wood debris. Elevation 2500m. | H.S. Yuan & H.C. Wang | MN463979 |  |  | MN170660 |  | MN463843 |
| *A. borealis* | QZ06002 | 2006/9/18 | M | 35º25' N, 111º58' E. Lishan Nature Reserve, Qinshui, Shanxi Prov. | *Betula* sp. Underground on living root. Elevation 2500m. | H.S. Yuan & H.C. Wang | MN463980 |  |  | MN170661 |  | MN463844 |
| *A. borealis* | Dai18954 | 2018/8/30 |  | 34º07' N, 107º53' E. Taibai Mountain, Shaanxi Prov. | Stump of *Betula* sp. | Y.C. Dai | MN463982 |  |  |  |  | MN463846 |
| *A. borealis* | Dai18958 | 2018/8/30 |  | 34º07' N, 107º53' E. Taibai Mountain, Shaanxi Prov. | Fallen trunk of *Betula* sp. | Y.C. Dai | MN463983 |  |  |  |  | MN463847 |
| *A. borealis* | 21SNJ18_2 | 2021/9/28 | M | 31º28' N, 109º58' E. Jiudahu Muyu, Shennongjia, Hubei Prov. | Unknown | J.W. Liu | PP442688 | PP444055 | PP443898 | PP356107 | PP443156 | PP443425 |
| *A. borealis* | 21SNJ19_3 | 2021/9/28 | M | 31º28' N, 109º58' E. Jiudahu Muyu, Shennongjia, Hubei Prov. | Unknown | J.W. Liu | PP442689 | PP444056 | PP443899 | PP356108 | PP443157 | PP443426 |
| *A. bruneocystidia* | QZ99011 | 1999/6/11 | H | 27º06' N, 100º12' E. Yulong Snow Mountain, Yunnan Prov. | *Picea likiangensis*. Rotten wood. Elevation 3500m. | K. Korhonen & Y.C. Dai | MN463932 | PP444083 | MT673928 | MN170628 | MT674075 | MN463794 |
| *A. bruneocystidia* | QZ99012_1 | 1999/6/11 | H | 27º06' N, 100º12' E. Yulong Snow Mountain, Yunnan Prov. | *Picea likiangensis*. Rotten wood. Elevation 3500m. | K. Korhonen & Y.C. Dai | MN463933 | PP444084 | PP443717 | MN170629 | MT674076 | MN463795 |
| *A. bruneocystidia* | QZ99028_2 | 1999/6/18 | H | 27º06' N, 100º12' E. Yulong Snow Mountain, Yunnan Prov. | *Picea likiangensis*. Rotten wood. Elevation 3500m. | K. Korhonen & Y.C. Dai | PP480536 | PP444085 | MT673929 | PP356124 | PP443173 | PP443445 |
| *A. bruneocystidia* | QZ00003 | 2000/6/20 | H | 27º05' N, 100º15' E. Dasuodao, Yulong Snow Mountain, Yunnan Prov. | *Picea likiangensis*. Rotten fallen trunk. Elevation 3356m. | G.F. Qin & J. Zhao |  |  |  |  |  |  |
| *A. bruneocystidia* | QZ00004 | 2000/6/20 | H | 27º05' N, 100º15' E. Dasuodao, Yulong Snow Mountain, Yunnan Prov. | *Picea likiangensis*. Dead stump. Elevation 3356m. | G.F. Qin & J. Zhao |  |  |  |  |  |  |
| *A. bruneocystidia* | QZ00005 | 2000/6/20 | H | 27º05' N, 100º15' E. Dasuodao, Yulong Snow Mountain, Yunnan Prov. | *Picea likiangensis*. Rotten wood debris. Elevation 3356m. | G.F. Qin & J. Zhao |  |  |  |  |  |  |
| *A. bruneocystidia* | QZ00006 | 2000/6/20 | H | 27º05' N, 100º15' E. Dasuodao, Yulong Snow Mountain, Yunnan Prov. | *Picea likiangensis*. Dead stump. Elevation 3356m. | G.F. Qin & J. Zhao | MN463935 |  | MT673917 | MN170630 | MT674063 | MN463797 |
| *A. bruneocystidia* | QZ00007_1 | 2000/6/20 | H | 27º05' N, 100º15' E. Dasuodao, Yulong Snow Mountain, Yunnan Prov. | Rotten angiosperm trunk. Elevation 4000m. | G.F. Qin & J. Zhao | MT647049 | PP444075 | PP444013 | MT644769 | PP443171 | PP443442 |
| *A. bruneocystidia* | QZ00008 | 2000/6/20 | H | 27º06' N, 100º13' E. Yunshanping, Yulong Snow Mountain, Yunnan Prov. | Base of dying angiosperms. Elevation 3240m. | G.F. Qin & J. Zhao | MN463936 |  | MT673918 | MN170631 | MT674064 | MN463798 |
| *A. bruneocystidia* | QZ00009_5 | 2000/6/20 | H | 27º06' N, 100º13' E. Yunshanping, Yulong Snow Mountain, Yunnan Prov. | *Abies ferreana*. Rotten fallen trunk. Elevation 3240m. | G.F. Qin & J. Zhao | MT647050 | PP444076 | MT673919 | MT644770 | MT674065 | MT674196 |
| *A. bruneocystidia* | QZ00010 | 2000/6/20 | H | 27º06' N, 100º13' E. Yunshanping, Yulong Snow Mountain, Yunnan Prov. | Mixed forest. Rotten wood debris. Elevation 3240m. | G.F. Qin & J. Zhao |  |  |  |  |  |  |
| *A. bruneocystidia* | QZ00011_7 | 2000/6/20 | H | 27º06' N, 100º13' E. Yunshanping, Yulong Snow Mountain, Yunnan Prov. | Mixed forest. Rotten wood debris. Elevation 3240m. | G.F. Qin & J. Zhao | MN463937 | PP444077 | MT673920 | MN170632 | MT674067 | MN463799/PP443443/PP443444 |
| *A. bruneocystidia* | QZ00012 | 2000/6/20 | H | 27º06' N, 100º13' E. Yunshanping, Yulong Snow Mountain, Yunnan Prov. | Mixed forest. Rotten wood. Elevation 3240m. | G.F. Qin & J. Zhao | MT647052 |  | PP443715 | MT644772 | MT674068 | MT674198 |
| *A. bruneocystidia* | QZ00013 | 2000/6/20 | H | 27º06' N, 100º13' E. Yunshanping, Yulong Snow Mountain, Yunnan Prov. | Mixed forest. Rotten wood. Elevation 3240m. | G.F. Qin & J. Zhao |  |  |  |  |  |  |
| *A. bruneocystidia* | QZ00014 | 2000/6/20 | H | 27º06' N, 100º13' E. Yunshanping, Yulong Snow Mountain, Yunnan Prov. | Mixed forest. Rotten wood. Elevation 3240m. | G.F. Qin & J. Zhao | MT647054 |  | MT673921 | MT644774 | MT674069 | MT674200 |
| *A. bruneocystidia* | QZ00015 | 2000/6/20 | H | 27º06' N, 100º13' E. Yunshanping, Yulong Snow Mountain, Yunnan Prov. | Mixed forest. Rotten wood. Elevation 3240m. | G.F. Qin & J. Zhao | MT647055 |  | MT673922 | MT644775 | MT674070 | MT674201 |
| *A. bruneocystidia* | QZ00019_2 | 2000/6/21 | H | 27º09' N, 100º15' E. Maoniuiping Yulong Snow Mountain, Yunnan Prov. | *Rhododendron* sp. Rotten root. Elevation 3500m. | G.F. Qin & J. Zhao | MT647056 | PP444078 | MT673923 | MT644776 | MT674071 | MT674202 |
| *A. bruneocystidia* | QZ00123 | 2000/9/17 | H | 27º80' N, 99º85' E. Tianshengqiao, Shangrila, Yunnan Prov. | Rotten angiosperm wood debris. Elevation 3300m. | J. Zhao & S.M. Tian | MN463938 | PP444079 | MT673924 | MN170633 | MT674072 | MN463800 |
| *A. bruneocystidia* | QZ00124 | 2000/9/17 | H | 27º80' N, 99º85' E. Tianshengqiao, Shangrila, Yunnan Prov. | Rotten angiosperm wood debris . Elevation 3300m. | J. Zhao & S.M. Tian |  |  |  |  |  |  |
| *A*. *bruneocystidia* | QZ19074 | 2019/10/16 | H | 29º33' N, 103º21' E. Chudian, Emei Mountain, Sichuan Prov. | Stump of *Cunninghamia lanceolata*. Elevation 1800m. | G.F. Qin | MT647057 | PP444080 | MT673925 | MT644777 |  | MT674203 |
| *A*. *bruneocystidia* | QZ19075_1 | 2019/10/16 | H | 29º33' N, 103º21' E. Above Chudian, Emei Mountain, Sichuan Prov. | Dead angiosperm trunk. Elevation 1850m. | G.F. Qin | MT647058 | PP444081 | MT673926 | MT644778 | MT674073 | MT674204 |
| *A*. *bruneocystidia* | QZ19078_1 | 2019/10/16 | H | 29º34' N, 103º21' E. Zhanglaoping, Emei Mountain, Sichuan Prov. | Dead angiosperm trunk. Elevation 1700m. | G.F. Qin | MT647059 | PP444082 | MT673927 | MT644779 | MT674074 | MT674205 |
| *A. bruneocystidia* | 21LJ5_2 | 2021/9/17 | H | 27º08' N, 100º13' E. Yunshanping, Yulong Snow Mountain, Yunnan Prov. | Host unkown. Elevation 3256m. | J.W. Liu | PP480534 | PP444074 | PP444012 | PP356123 | PP443170 | PP443440 |
| *A. bruneocystidia* | 21LJ10 | 2021/9/17 | H | 27º08' N, 100º13' E. Yunshanping, Yulong Snow Mountain, Yunnan Prov. | Host Unkown. Elevation 3229m. | J.W. Liu | PP480533 | PP444073 | PP443714 | PP356122 | PP443169 | PP443441 |
| *A. cepistipes* | QZ99024 | 1999/01/16 |  | 60º10' N, 24º56' E. Kaisaniemi Botanic Garden, Helsinki, Finland | *Tilia* sp. | G.F. Qin |  |  |  |  |  |  |
| *A. cepistipes* | QZ99134 | 1999/01/16 |  | 60º10' N, 24º56' E. Kaisaniemi Botanic Garden, Helsinki, Finland | *Tilia* sp. | G.F. Qin |  |  |  |  |  |  |
| *A. cepistipes* | QZ19041 | 2019/10 |  | 53º10' N, 27º48' E. Belarus | *Picea* sp. | Y.C. Dai |  |  |  |  |  |  |
| *A. cepistipes* | QZ19042 | 2019/10 |  | 53º10' N, 27º48' E. Belarus | *Populus* sp. | Y.C. Dai |  |  |  |  |  |  |
| *A. gallica* | QZ96004_27 | 1996/8/19 | B | 43º06' N, 128º01' E. Jingou, Huangnihe, Jilin Prov. | *Pinus koraiensis*. Solitary on living roots or caespitose on dead stump. Elevation 500m. | G.F. Qin | MN463878 | PP444156 | MT673940 | MN170581 | MT674087 | MN463737 |
| *A. gallica* | QZ96012_35 | 1996/8/27 | B | 42º03' N, 128º01' E. Baihe, Changbai Mountain, Jilin Prov. | *Prunus salicina*. Base of dying tree. Elevation 700m. | G.F. Qin | MN463880 | PP444158 | PP443737 | MN170583 | PP443237 | MN463739 |
| *A. gallica* | QZ96032_31 | 1996/8/31 | B | 42º03' N, 128º01' E. Erdao, Changbai Mountain, Jilin Prov. | *Quercus mongolica*. Trunk of living tree, 1m above ground. Elevation 700m. | G.F. Qin | MN463883 | PP444162 | MT673943 | MN170586 | PP443241 | MN463743 |
| *A. gallica* | QZ96040_17 | 1996/9/3 | B | 42º03' N, 128º01' E. Erdao, Changbai Mountain, Jilin Prov. | Rotten angiosperm wood debris. Elevation 700m. | G.F. Qin | PP442750 | PP444164 | PP443741 | PP356192 | PP443244 | PP443501 |
| *A. gallica* | QZ02102 | 2002/8/10 | B | 44º90' N, 130º50' E. Muling, Heilongjiang Prov. | Unknown. Elevation 1000m. | G.T. Yang & L.F. Sun | PP442737 | PP444136 | PP443723 | PP356177 | PP443223 | PP443486 |
| *A. gallica* | QZ02122 | 2002/8/13 | B | 45º30' N, 130º30' E. Linkou, Heilongjiang Prov. | Unknown. Elevation 800m. | G.T. Yang & L.F. Sun | PP442740 | PP444139 | PP443726 | PP356180 | PP443226 | PP443489 |
| *A. gallica* | QZ02126 | 2002/8/14 | B | 44º90' N, 130º50' E. Chaihe, Heilongjiang Prov. | *Larix* sp. Elevation 800m. | G.T. Yang & L.F. Sun | PP442741 | PP444140 | PP443727 | PP356181 | PP443227 | PP443490 |
| *A. gallica* | QZ02147 | 2002/8/15 | B | 44º06' N, 129º03' E. Dahailin, Heilongjiang Prov. | Mixed forest. Rotten wood debris. Elevation 1000m. | G.T. Yang & L.F. Sun |  | PP444141 | PP443728 | PP356182 | PP443228 | PP443491 |
| *A. gallica* | QZ02163 | 2002/8/22 | B | Heihe, Heilongjiang Prov. | *Quercus mongolica.* Elevation 1000m. | G.T. Yang & L.F. Sun | PP442742 | PP444142 | PP443729 | PP356183 | PP443229 | PP443492 |
| *A. gallica* | QZ03000_4 | 2003/8/12 | B | 40º05' N, 124º00' E. Fengcheng, Liaoning Prov. | Unknown | G.F. Qin & J. Zhao | PP442744 | PP444144 | PP443730 | PP356185 | PP443231 | PP443494 |
| *A. gallica* | QZ19001_4 | 2019/9/6 | B | 42º35' N, 128º08' E. Changbai Mountain, Jilin Prov. | Rotten wood debris. Elevation 1000m. | W.M. Qin & Y.L. Wei | MT647062 | PP444147 | MT673933 | MT644782 | MT674081 | MT674208 |
| *A. gallica* | QZ19014_3 | 2019/9/7 | B | 41º57' N, 123º40' E. Qipan Mountain, Shenyang, Liaoning Prov. | Solitary at base of living *Cerasus pseudocerasus.* | G.F. Qin | MT647065 | PP444149 | MT673936 | MT644785 | MT674083 | MT674211 |
| *A. gallica* | QZ19036_1 | 2019/9/28 | B | 31º06' N, 111º13' E. Wuduhe, Yiling district, Yichang, Hubei Prov. | Solitary or caespitose on rotten angiosperm branches, from cultivated beds of *Gastrodia elata*. | Y.C. Dai | MT647067 | PP444151 | MT673938 | MT644787 | MT674085 | MT674213 |
| *A. gallica* | QZ20021_1 | 2020/9/21 | B | 30º08' N, 110º57' E. Houhe, Wufeng, Hubei Prov. | Angiosperms. Elevation 1700m. | G.F. Qin | PP442746 | PP444153 | PP443734 | PP356187 | PP443234 | PP443496 |
| *A. gallica* | QZ20029_1 | 2020/9/25 | B | 30º08' N, 110º57' E. Houhe, Wufeng county, Hubei Prov. | Angiosperms. Elevation 1700m. | G.F. Qin | PP442747 | PP444154 | PP443735 | PP356188 | PP443235 | PP443497 |
| *A. korhonenii* | 22DWS1_4 | 2022/5/24 | Q | 22º55' N, 103º41' E. Daweishan Nature Reserve, Pingbian, Yunnan Prov. | Rotten angiosperm wood. Elevation 1800m. | J.W. Liu | PP442797 | PP444211 | PP443782/  PP353637 | PP356199 | PP443404 | PP443687 |
| *A. korhonenii* | 22DWS2_2 | 2022/5/24 | Q | 22º55' N, 103º41' E. Daweishan Nature Reserve, Pingbian, Yunnan Prov. | Rotten angiosperm wood. Elevation 1800m. | J.W. Liu | PP442798 | PP444212 | PP353638 | PP356200 | PP443406 | PP443688 |
| *A. korhonenii* | 22DWS3_1 | 2022/5/24 | Q | 22º55' N, 103º41' E. Daweishan Nature Reserve, Pingbian, Yunnan Prov. | Rotten angiosperm wood. Elevation 1800m. | J.W. Liu | PP442799 | PP444213 | PP353639 | PP356201 | PP443407 | PP443689 |
| *A. korhonenii* | 22DWS4_1 | 2022/5/24 | Q | 22º55' N, 103º41' E. Daweishan Nature Reserve, Pingbian, Yunnan Prov. | Rotten angiosperm wood. Elevation 1800m. | J.W. Liu | PP442800 | PP444214 | PP353640 | PP356202 | PP443408 | PP443690 |
| *A. korhonenii* | 22DWS5_4 | 2022/5/24 | Q | 22º55' N, 103º41' E. Daweishan Nature Reserve, Pingbian, Yunnan Prov. | Rotten angiosperm wood. Elevation 1800m. | J.W. Liu | PP442801 | PP444215 | PP353641 | PP356203 | PP443409 | PP443542 |
| *A. korhonenii* | 22DWS6_1 | 2022/5/24 | Q | 22º55' N, 103º41' E. Daweishan Nature Reserve, Pingbian, Yunnan Prov. | Rotten angiosperm wood. Elevation 1800m. | J.W. Liu | PP442802 | PP444216 | PP353642 | PP356204 | PP443410 | PP443543 |
| *A. korhonenii* | 22DWS7_3 | 2022/5/24 | Q | 22º55' N, 103º41' E. Daweishan Nature Reserve, Pingbian, Yunnan Prov. | Rotten angiosperm wood. Elevation 1800m. | J.W. Liu | PP442803 | PP444217 | PP353643 | PP356205 | PP443411 | PP443691 |
| *A. korhonenii* | 22DWS8_2 | 2022/5/24 | Q | 22º55' N, 103º41' E. Daweishan Nature Reserve, Pingbian, Yunnan Prov. | Rotten angiosperm wood. Elevation 1800m. | J.W. Liu | PP442804 | PP444218 | PP353644 | PP356206 | PP443240 | PP443692 |
| *A. korhonenii* | Dai25210 | 2023/6/28 | Q | 23º942' N, 101º501' E. Ailaoshan Nature Reserve, Gasa, Xinping, Yunnan Prov. | Solitary or caespitose on rotten wood of angiosperm. Elevation 2455m. | Y.C. Dai | PP442805 | PP444219 | PP353645 | PP356207 | PP443412 | PP443693 |
| *A. korhonenii* | Dai25211 | 2023/6/28 | Q | 23º942' N, 101º501' E. Ailaoshan Nature Reserve, Gasa, Xinping, Yunnan Prov. | Caespitose on fallen angiosperm trunk. Elevation 2455m. | Y.C. Dai | PP442806 |  | PP353646 | PP356208 | PP443413 | PP443565 |
| *A. korhonenii* | Dai25212 | 2023/6/28 | Q | 23º942' N, 101º501' E. Ailaoshan Nature Reserve, Gasa, Xinping, Yunnan Prov. | Fallen angiosperm trunk. Elevation 2455m. | Y.C. Dai | PP442807 |  | PP353647 | PP356209 | PP443414 | PP443540 |
| *A. korhonenii* | Dai25213 | 2023/6/28 | Q | 23º942' N, 101º501' E. Ailaoshan Nature Reserve, Gasa, Xinping, Yunnan Prov. | Fallen angiosperm trunk. Elevation 2455m. | Y.C. Dai | PP442808 |  | PP353648 | PP356210 | PP443251 | PP443694 |
| *A. luteopileata* | QZ92018_2 | 1992/8/25 | B | 42º03' N, 128º01' E. Baihe, Changbai Mountain, Jilin Prov. | Unknown | W. He | PP480545 | PP444227 | PP443948 | PP356216 | PP443104 | PP443554 |
| *A. luteopileata* | QZ93022_11 | 1993/8/21 | C | 42º03' N, 128º01' E. Baihe, Changbai Mountain, Jilin Prov. | *Ulmus* sp. Rotten fallen wood. Elevation 740m. | W. He & B. Wang | PP480546 | PP444228 | PP443949 | PP356217 | PP443105 | PP443555 |
| *A. luteopileata* | QZ96014_16 | 1996/8/28 | C | 42º00' N, 128º01' E. Yuehualin, Changbai Mountain, Jilin Prov. | *Betula ermanii*. Caespitose on dead fallen trunk. Elevation 1500m. | G.F. Qin | MN463885 | PP444229 | PP443950 | PP356218 | PP443106 | MN463745 |
| *A. luteopileata* | QZ96018 | 1996/8/28 | C | 42º04'N, 128º03' E. Dixiasenlin, Changbai Mountain, Jilin Prov. | *Abies nephrolepis*. Solitary on fallen trunk. Elevation1500m | G.F. Qin | MN463886 | PP444230 | PP443951 | MN170588 | PP443107 | MN463746 |
| *A. luteopileata* | QZ96027 | 1996/8/30 | C | 42º03' N, 128º01' E. Baihe, Changbai Mountain, Jilin Prov. | Rotten angiosperm wood debris. Elevation 700m. | G.F. Qin | MN463881 | PP444231 | MT673945 | MN170584 | MT674091 | MN463740 |
| *A. luteopileata* | QZ96029 | 1996/8/31 | C | 42º03' N, 128º01' E. Baihe, Changbai Mountain, Jilin Prov. | Rotten angiosperm wood debris. Elevation 700m. | G.F. Qin | MT647071 | PP444232 | MT673946 | MT644790 | MT674092 | MT674216 |
| *A. luteopileata* | QZ96036 | 1996/9/2 | C | 42º04'N, 128º03' E. Dark conifers, Changbai Mountain, Jilin Prov. | *Abies nephrolepis*. solitary on rotten trunk. Elevation 1600m. | G.F. Qin |  | PP444233 | PP443952 | PP356219 | PP443108 | PP443556 |
| *A. luteopileata* | QZ96038_1 | 1996/9/3 | C | 42º01' N, 128º01' E. Dixiasnelin, Changbai Mountain, Jilin Prov. | *Abies nephrolepis*. Solitary on rotten trunk. Elevation 1600m. | G.F. Qin | MT647072 | PP444234 | MT673947 | MT644791 | MT674093 | MT674217 |
| *A. luteopileata* | QZ97043_11 | 1997/9/14 | C | 42º20' N, 128º01' E. Huangsongpu, Changbai Mountain, Jilin Prov. | Broad-leaved forest, rotten fallen trunk. Elevation 1000m. | G.F. Qin & J. Zhao | MN463887 | PP444235 | PP443953 | MN170589 | PP443109 | MN463747 |
| *A. luteopileata* | QZ97047_6 | 1997/9/14 | C | 42º12' N, 128º09' E. Huangsongpu, Changbai Mountain, Jilin Prov. | Fallen wood of conifers. Elevation 1000m. | G.F. Qin & J. Zhao | PP480547 | PP444236 | PP443954 | PP356220 | PP443110 | PP443557 |
| *A. luteopileata* | QZ97052_6 | 1997/9/16 | C | 42º12' N, 128º09' E. Huangsongpu, Changbai Mountain, Jilin Prov. | *Betula platyphylla*. Solitary or caespitose on rotten fallen trunk. Elevation 1000m. | G.F. Qin & J. Zhao | PP480548 | PP444237 | PP443955 | PP356221 | PP443111 | PP443558 |
| *A. luteopileata* | QZ97053 | 1997/9/16 | C | 42º12' N, 128º09' E. Huangsongpu, Changbai Mountain, Jilin Prov. | *Tilia amurensis*. Rotten fallen trunk. Elevation 1000m. | G.F. Qin & J. Zhao | MN463888 | PP444238 | PP443956 | MN170590 | PP443112 | MN463748 |
| *A. luteopileata* | QZ99109 | 1999/9/6 | C | 47º02' N, 128º09' E. Liangshui, Dailing, Heilongjiang Prov. | *Betula platyphylla*. Root of living tree, Elevation 400m. | J. Zhao & S.M. Tian |  |  |  |  |  |  |
| *A. luteopileata* | QZ99110 | 1999/9/6 | C | 47º02' N, 128º09' E. Liangshui, Dailing, Heilongjiang Prov. | *Betula platyphylla*. Rotten fallen trunk. Elevation 400m. | J. Zhao & S.M. Tian |  |  |  |  |  |  |
| *A. luteopileata* | QZ99111 | 1999/9/6 | C | 47º02' N, 128º09' E. Liangshui, Dailing, Heilongjiang Prov. | *Picea koraiensis*. Trunk of living tree. Elevation 400m. | J. Zhao & S.M. Tian |  |  |  |  |  |  |
| *A. luteopileata* | QZ99114_2 | 1999/9/7 | C | 47º02' N, 128º09' E. Liangshui, Dailing, Heilongjiang Prov. | *Abies & Picea* conifer forest. Rotten wood debris. Elevation 400m | J. Zhao & S.M. Tian | PP480549 | PP444239 | PP443957 | PP356222 | PP443113 | PP443559 |
| *A. luteopileata* | QZ99115_4 | 1999/9/7 | C | 47º02' N, 128º09' E. Liangshui, Dailing, Heilongjiang Prov. | Rotten angiosperms. Elevation 400m. | J. Zhao & S.M. Tian | MN463889 | PP444240 | PP443958 | MN170591 | PP443114 | MN463749 |
| *A. luteopileata* | QZ03008_1 | 2003/8/24 | C | 42º12' N, 128º09' E. Huangsongpu, Changbai Mountain, Jilin Prov. | Rotten angiosperm wood debris s. Elevation 1000m. | G.F. Qin & J. Zhao | MN463890 | PP444220 | PP443938 | MN170592 | PP443098 | MN463750 |
| *A. luteopileata* | QZ19004_1 | 2019/9/6 | C | 42º03' N, 128º01' E. Baihe, Changbai Mountain, Jilin Prov. | *Ulmus pumila*. | W.M. Qin & Y. L. Wei | MT647069 | PP444222 | MT673944 | MT644788 | MT674090 | MT674214 |
| *A. luteopileata* | QZ21009_1 | 2021/9/8 | C | 42º05'N, 128º04' E. Dixiasenlin, Changbai Mountain, Jilin Prov. | *Abies nephrolepis*. Solitary on fallen trunk. Elevation1500m | G.F. Qin | PP480541 | PP444223 | PP443944 | PP356212 | PP443100 | PP443550 |
| *A. luteopileata* | QZ21023 | 2021/9/8 | C | 42º03'N, 128º03' E. Yuehualin conifers, Changbai Mountain, Jilin Prov. | *Betula ermanii.* Rotten wood debris. Elevation1850m. | G.F. Qin | PP480543 | PP444225 | PP443946 | PP356214 | PP443102 | PP443552 |
| *A. luteopileata* | QZ21024_1 | 2021/9/8 | C | 42º03'N, 128º03' E. Yuehualin, Changbai Mountain, Jilin Prov. | *Betula ermanii.* Rotten fallen trunk. ltitude1850m. | G.F. Qin | PP480544 |  |  | PP356215 | PP443103 | PP443553 |
| *A. mellea* | QZ00111_3 | 2000/9/14 | K | 25º07' N, 100º02' E. Yunshanping, Yulong Snow Mountain, Yunnan Prov. | Mixed forest. Rotten angiosperm wood. Elevation 3240m. | J. Zhao & S.M. Tian | MN463962 | PP444263 | MT673949 | MN170652 | MT674094 | MN463825 |
| *A. mellea* | QZ19052_2 | 2019/10/10 | K | 30º08' N,110º57' E. Yangzixi, Houhe Nature Reserve, Wufeng, Hubei Prov. | Caespitose on base of living angiosperm tree. | G.F. Qin | MT647073 | PP444264 | MT673950 | MT644792 | MT674095 | MT674218 |
| *A. mellea* | QZ19105_2 | 2019/10/21 | K | 30º08' N,110º57' E. Yangzixi, Houhe Nature Reserve, Wufeng, Hubei Prov. | Caespitose on base of dead stump of *Pyrus* sp. Elevation 1700m. | G.F. Qin | MT647075 | PP444266 | MT673948 | MT644794 | MT674097 | MT674219 |
| *A. mellea* | QZ20057_1 | 2020/10/13 | K | 30º08' N,110º57' E. Yangzixi, Houhe Nature Reserve, Wufeng, Hubei Prov. | Caespitose on base of dead angiosperm stump. Elevation 1700m. | G.F. Qin | PP442681 | PP444267 | PP443977 | PP356244 | PP443273 | PP443676 |
| *A. mellea* | 21LJ11_2 | 2020/10/13 | K | 27º08' N, 100º13' E. Yunshanping, Yulong Snow Mountain, Yunnan Prov. | Unkown. Elevation 3330m. | J.W. Liu | PP442663 | PP444256 | PP443970 | PP356229 | PP443259 | PP443703 |
| *A. mellea* | 22KM1_2 | Jun-22 | K | Kunming, Yunnan Prov. | Unkown. | J.W. Liu | PP442677 | PP444260 | PP443974 | PP356233 | PP443263 | PP443675 |
| *A. mellea* | QZ23005 | 2023/5/21 | K | 22º55' N, 103º41' E. Daweishan Nature Reserve, Pingbian, Yunnan Prov. | Caespitose on fallen angiosperm trunk. Elevation 2500m. | G.F. Qin | PP442684 | PP444270 | PP443980 | PP356247 | PP443276 | PP443705 |
| *A. mellea* spp. *nipponica* | QZ98001_53 | 1998/4/23 | G | 27º17' N, 112º41' E. Hengshan, Hunan Prov. | *Cyclobalanopsis* sp. | G.F. Qin & | PP442661 | PP444253 | PP443967 |  | PP443256 | PP443699 |
|  |  |  |  |  |  | C.R. Liu |  |  |  |  |  |  |
| *A. mellea* spp. *nipponica* | QZ99043_9 | 1999/6/18 | G | 25º01' N, 102º40' E. Kunming, Yunnan prov. | *Keteleeria* sp. | K. Korhonen & Y.C. Dai | MN463918 | PP444254 | PP443968 | PP356227 | PP443257 | PP443701 |
| *A. mellea* spp. *nipponica* | QZ00020 | 2000/6/21 | G | 25º07' N, 100º02' E. Maoniuping, Yulong Snow Mountain, Yunnan Prov. | *Pinus densata*. Base of living tree. Elevation 2500m | G.F. Qin & J. Zhao | MN463921 | PP444249 | PP443964 | MN170613 | PP443253 | MN463785 |
| *A. mellea* spp. *nipponica* | QZ01010 | 2001/8/30 | G | 31º05' N, 110º04' E. Xujiazhuang, Shennongjia Mountain, Hubei Prov. | *Ailanthus altissima* Swingleh. | J. Zhao | PP442675 | PP444250 | PP443965 | PP356224 | PP443254 | PP443700 |
| *A. mellea* spp. *nipponica* | QZ19037 | Sep-20 | G | Taiwan Prov. | Unknown | S.H. Wu & Z.Z. Chen | MT647076 | PP444251 | MT673952 | MT644795 | MT674098 | MT674221 |
| *A. mellea* spp. *nipponica* | QZ83003 | Aug-83 | G | Nagasaki, Japan | *Chamaecyparis* sp. | J. Rishbeth | PP442660 | PP444252 | PP443966 | PP356225 | PP443255 |  |
| *A. ostoyae* | QZ96044_10 | 1996/9/26 | D | 43º06' N, 128º00' E. Huangnihe, Dunhua, Jilin Prov. | Unknown | G.F. Qin | PP442719 | PP444118 | PP443924 | PP356282 | PP443311 | PP443469 |
| *A. ostoyae* | QZ97048_14 | 1997/9/15 | D | 42º03' N, 128º01' E. Erdao, Changbai Mountain, Jilin Prov. | Scattered on angiosperm wood debris. Elevation 700m. | G.F. Qin & J. Zhao | PP442723 | PP444123 | PP443929 | PP356284 | PP443316 | PP443472 |
| *A. ostoyae* | QZ97054_20 | 1997/9/16 | D | 42º2' N, 128º1' E. Huangsongpu, Changbai Mountain, Jilin Prov. | Rotten angiosperm wood debris. Elevation 1000m. | G.F. Qin & J. Zhao | PP442725 | PP444125 | PP443931 | PP356286 | PP443318 | PP443474 |
| *A. ostoyae* | QZ97058C_15 | 1997/10/10 | D | 43º33' N, 128º01' E. Huangnihe, Dunhua, Jilin Prov. | Unknown | J. Zhao | PP442727 | PP444128 | PP443932 | PP356288 | PP443319 | PP443475 |
| *A. ostoyae* | QZ02171 | 2002/8/24 | D | 50º02' N, 127º05' E. Heihe, Heilongjiang Prov. | Rotten angiosperm wood debris. Elevation 200m. | G.T. Yang & L.F. Sun | PP442703 | PP444095 | PP443911 | PP356267 | PP443294 | PP443454 |
| *A. ostoyae* | QZ02176 | 2002/8/28 | D | 49º02' N, 125º02' E. Nenjiang, Heilongjiang Prov. | Unknown. Elevation 400m. | G T. Yang & L.F. Sun | PP442706 | PP444098 | PP443914 | PP356270 | PP443297 | PP443457 |
| *A. ostoyae* | QZ02180 | 2002/8/28 | D | 49º02' N, 125º02' E. Nenjiang, Heilongjiang Prov. | *Quercus mongolica*. Elevation 400m. | G.T. Yang & L.F. Sun | PP442707 | PP444099 | PP444007 | PP356271 | PP443298 | PP443458 |
| *A. ostoyae* | QZ19035_1 | 2019/9/25 | D | 41º57' N, 123º39' E. Puhe, Shenyang, Liaoning Prov. | Unknown | G.F. Qin | MT647079 | PP444103 | MT673957 | MT644799 | PP443299 | MT674224 |
| *A. ostoyae* | QZ21001D_5 | 2021/9/7 | D | 42º26' N, 128º10' E. Baihe, Changbai Mountain, Jilin Prov. | Unknown | G.F. Qin | PP442708 | PP444104 | PP443915 | PP356272 | PP443300 | PP443459 |
| *A. ostoyae* | QZ21015_1 | 2021/9/8 | D | 42º05' N, 128º04' E. Dixiasenlin, Changbai Mountain, Jilin Prov. | *Abies nephrolepis.* | G.F. Qin | PP442710 | PP444106 | PP443917 | PP356274 | PP443302 | PP443461 |
| *A. ostoyae* | QZ21035_2 | 2021/9/9 | D | 42º13' N, 128º07' E. Huangsongpu, Changbai Mountain, Jilin Prov. | *Abies nephrolepis.* | G.F. Qin | PP442711 | PP444107 | PP443918 | PP356275 | PP443303 | PP443462 |
| *A. ostoyae* | QZ21042_4 | 2021/9/9 | D | 42º16' N, 128º09' E. Antu, Changbai Mountain, Jilin Prov. | *Phellodendron amurense.* | G.F. Qin | PP442712 | PP444108 | PP443919 | PP356276 | PP443304 | PP443463 |
| *A. ostoyae* | QZ21044_2 | 2021/9/25 | D | 41º18' N, 124º10' E. Xiaoshi, Benxi, Liaoning Prov. | Unknown | G.F. Qin | PP442714 | PP444110 | PP443921 | PP356278 | PP443306 | PP443465 |
| *A. ostoyae* | QZ23027_1 | 2023/09/03. | D | 47º58' N, 121º12' E. Taerqi, Yakeshi, Inner Mongolia Autonomous Region | *Betula platyphylla.* Dead stump. Elevation 1000m | G.F. Qin | PP442716 | PP444112 | PP444008 | PP356280 | PP443308 | PP443467 |
| *A. pungentisquamosa* | QZ04047 | 2004/9/24 | P | 30º04' N, 110º32' E. Houhe Nature Reserve, Wufeng, Hubei Prov. | Mixed forest. Rotten  angiosperm wood. Elevation 1600m. | Y.L. Wei & H.S. Yuan | MN463996 | PP444285 |  | MN170671 | PP443325 | MN463861 |
| *A. pungentisquamosa* | QZ19053_1 | 2019/10/10 | P | 30º04' N, 110º32' E. Yangzixi, Houhe Nature Reserve, Wufeng, Hubei Prov. | Mixed forest, Rotten wood debris. Elevation 1700m. | G.F. Qin | MT647086 | PP444287 | MT673969 | MT644806 | PP443327 | MT674230 |
| *A. pungentisquamosa* | QZ19055_3 | 2019/10/10 | P | 30º04' N, 110º32' E. Yangzixi, Houhe Nature Reserve, Wufeng, Hubei Prov. | Caespitose on dead stump of *Cerasus pseudocerasus*. Elevation 1700m. | G.F. Qin | MT647088 | PP444288 | MT673971 | MT644808 | MT674111 | MT674232 |
| *A. pungentisquamosa* | QZ19056_4 | 2019/10/10 | P | 30º04' N, 110º32' E. Yangzixi, Houhe Nature Reserve, Wufeng, Hubei Prov. | Dead angiosperm stump. Elevation 1700m. | G.F. Qin | MT647089 | PP444289 | MT673972 | MT644809 | MT674112 | MT674233 |
| *A. pungentisquamosa* | QZ19058 | 2019/10/10 | P | 30º04' N, 110º32' E. Yangzixi, Houhe Nature Reserve, Wufeng, Hubei Prov. | Caespitose on rotten angiosperm stump. Elevation 1700m. | G.F. Qin | MT647090 |  | MT673973 | MT644810 | MT674113 | MT674234 |
| *A. pungentisquamosa* | QZ19059 | 2019/10/10 | P | 30º04' N, 110º32' E. Yangzixi, Houhe Nature Reserve, Wufeng, Hubei Prov. | Rotten wood debris. Elevation 1700m. | G.F. Qin | MT647091 |  |  | MT644811 |  | MT674235 |
| *A. pungentisquamosa* | QZ19060_1 | 2019/10/10 | P | 30º04' N, 110º32' E. Yangzixi, Houhe Nature Reserve, Wufeng, Hubei Prov. | Caespitose on rotten trunk of *Salix babylonica*. Elevation 1100m. | G.F. Qin | MT647092 | PP444290 | MT673974 | MT644812 | MT674114 | MT674236 |
| *A. pungentisquamosa* | QZ19061 | 2019/10/12 | P | 30º02' N, 110º12' E. Heiwanya, Mulinzi Nature Reserve, Hefeng, Hubei Prov. | Caespitose on dead stump of *Betula albosinensis*. Elevation 1200m. | G.F. Qin | MT647093 |  | MT673975 | MT644813 |  | MT674237 |
| *A. pungentisquamosa* | QZ19062_2 | 2019/10/12 | P | 30º02' N, 110º12' E. Heiwanya, Mulinzi Nature Reserve, Hefeng, Hubei Prov. | Caespitose on fallen trunk of *Sycopsis sinensis*. Elevation 1300m. | G.F. Qin | MT647094 | PP444291 | MT673976 | MT644814 | MT674115 | MT674238 |
| *A. pungentisquamosa* | QZ19063 | 2019/10/12 | P | 30º02' N, 110º12' E. Heiwanya, Mulinzi Nature Reserve, Hefeng, Hubei Prov. | Solitary on rotten stump of *Betula albosinensis*. Elevation 1300m. | G.F. Qin | MT647095 |  | MT673977 | MT644815 | MT674116 | MT674239 |
| *A. pungentisquamosa* | QZ19065_1 | 2019/10/12 | P | 30º02' N, 110º12' E. Heiwanya, Mulinzi Nature Reserve, Hefeng, Hubei Prov. | Caespitose on fallen angiosperm trunk. Elevation 1500m. | G.F. Qin | MT647097 | PP444292 | MT673978 | MT644817 | MT674117 | MT674241 |
| *A. pungentisquamosa* | QZ19066 | 2019/10/12 | P | 30º02' N, 110º12' E. Heiwanya, Mulinzi Nature Reserve, Hefeng, Hubei Prov. | Caespitose on fallen trunk of *Fagus* sp. Elevation 1600m. | G.F. Qin | MT647098 |  | MT673979 | MT644818 |  | MT674242 |
| *A. pungentisquamosa* | QZ19068_2 | 2019/10/12 | P | 30º02' N, 110º12' E. Heiwanya, Mulinzi Nature Reserve, Hefeng, Hubei Prov. | Solitary or caespitose on rotten root of angiosperm. Elevation 1700m. | G.F. Qin | MT647100 | PP444293 | PP443787 | MT644820 | PP443328 | MT674244 |
| *A. pungentisquamosa* | QZ19069 | 2019/10/13 | P | 30º03' N, 110º13' E. Changwan, Mulinzi Nature Reserve, Hefeng, Hubei Prov. | Caespitose on fallen angiosperm trunk. Elevation 1600m. | G.F. Qin | MT647101 |  | MT673981 | MT644821 | MT674119 | MT674245 |
| *A. pungentisquamosa* | QZ19070_3 | 2019/10/13 | P | 30º03' N, 110º13' E. Changwan, Mulinzi Nature Reserve, Hefeng, Hubei Prov. | Solitary on fallen angiosperm trunk. Elevation 1600m. | G.F. Qin | MT647102 | PP444294 | MT673982 | MT644822 | MT674120 | MT674246 |
| *A. pungentisquamosa* | QZ19071_4 | 2019/10/13 | P | 30º03' N, 110º13' E. Changwan, Mulinzi Nature Reserve, Hefeng, Hubei Prov. | Caespitose on fallen angiosperm trunk. Elevation 1600m. | G.F. Qin | MT647103 | PP444295 | PP443788 | MT644823 | MT674121 | MT674247 |
| *A. pungentisquamosa* | QZ19073_3 | 2019/10/15 | P | 29º34' N, 103º21' E. Zhanglaoping, Emei Mountain, Meishan, Sichuan Prov. | Solitary to caespitose on fallen angiosperm trunk. Elevation 1600m. | G.F. Qin | MT647104 | PP444296 | MT673983 | MT644824 | MT674122 | MT674248 |
| *A. pungentisquamosa* | QZ20033_5 | 2020/10/10 | P | 30º04' N, 110º32' E. Yangzixi, Houhe Nature Reserve, Wufeng, Hubei Prov. | Rotten angiosperm wood. Elevation 1700m. | G.F. Qin | PP442648 | PP444297 | PP443789 | PP356294 | PP443329 | PP443577 |
| *A. pungentisquamosa* | QZ20041_5 | 2020/10/10 | P | 30º04' N, 110º32' E. Yangzixi, Houhe Nature Reserve, Wufeng, Hubei Prov. | Rotten angiosperm wood. Elevation 1700m. | G.F. Qin | PP442649 | PP444298 | PP443790 | PP356295 | PP443330 | PP443578 |
| *A. pungentisquamosa* | Dai26842_1 | 2023/10/25 | P | 29º71'N, 95º58'E. Medog Nyingchi, Xizang Autonomous Region | *Abies* sp. | Y.C. Dai | PP442645 | PP444283 | PP443784 | PP356291 | PP443323 | PP443575 |
| *A. pungentisquamosa* | Dai26843_1 | 2023/10/25 | P | 29º71'N, 95º58'E. Medog Nyingchi, Xizang Autonomous Region | *Abies* sp. | Y.C. Dai | PP442646 | PP444284 | PP443785 | PP356292 | PP443324 | PP443576 |
| *A. sinapina* | QZ96008_11 | 1996/8/24 | A | 42º10' N, 128º10' E. Front gate, Changbai Mountain, Jilin Prov. | *Salix*. Dead stump. Elevation 1100m. | G.F. Qin | MN463872 | PP444307 | MT673998 | MN170575 | PP443340 | MN463730 |
| *A. sinapina* | QZ96016_10 | 1996/8/29 | A | 42º03' N, 128º03' E. Yuehualindai, Changbai Mountain, Jilin Prov. | *Betula ermanii*. Base of decline tree. Elevation 1700m. | G.F. Qin | MN463873 | PP444308 | MT673999 | MT644838 | MT674136 | MN463731 |
| *A. sinapina* | QZ96017_10 | 1996/8/28 | A | 42º01' N, 128º01' E. Dixiasenlin, Changbai Mountain, Jilin Prov. | Fallen trunk of *Abies* sp. Elevation 1500m. | G.F. Qin | PP442830 | PP444309 | PP443799 | PP356372 | PP443341 | PP443587 |
| *A. sinapina* | QZ96025_1 | 1996/8/30 | A | 42º03' N, 128º03' E. Yuehualindai, Changbai Mountain, Jilin Prov. | *Larix olgensis*. Caespitose at base of dying tree. Elevation 1750m | G.F. Qin | MN463877 | PP444310 | MT674000 | MN170579 | MT674137 | MN463735 |
| *A. sinapina* | QZ96041_12 | 1996/9/3 | A | 42º30' N, 128º01' E. Baihe, Changbai Mountain, Jilin Prov. | Angiospermous forest. Rotten wood debris. Elevation 700m | G.F. Qin | MT647112 | PP444311 | MT674001 | MT644839 | MT674138 | MT674257 |
| *A. sinapina* | QZ96042_27 | 1996/9/8 | A | 47º58' N, 121º12' E. Taerqi, Yakeshi, Inner Mongolia Autonomous Region | *Betula platyphylla.* Rotten wood debris. Elevation 1000m. | G.F. Qin | MN463874 | PP444312 | MT674002 | MN170576 | MT674139 | MN463732 |
| *A. sinapina* | QZ99116_7 | 1999/9/7 | A | 47º01' N, 128º08' E. Liangshui forestry farm, Dailing, Heilongjiang Prov. | *Abies* & *Betula*. Rotten wood debris. Elevation 400m | J. Zhao & S.M. Tian | PP442831 | PP444313 | PP443800 | MN170580 | PP443342 | MN463736 |
| *A. sinapina* | QZ23016_1 | 2023/8/22 | A | 47º58' N, 121º12' E. Taerqi, Yakeshi, Inner Mongolia Autonomous Region | *Betula platyphylla.* Rotten wood debris. Elevation 1000m. | G.F. Qin | PP442821 | PP444299 | PP443791 | PP356364 | PP443333 | PP443579 |
| *A. sinapina* | QZ23020_1 | 2023/8/22 | A | 47º58' N, 121º12' E. Taerqi, Yakeshi, Inner Mongolia Autonomous Region | *Betula platyphylla.* Rotten wood debris. Elevation 1000m. | G.F. Qin | PP442823 | PP444301 | PP443793 | PP356366 | PP443334 | PP443581 |
| *A. sinapina* | QZ23022_1 | 2023/8/28 | A | 47º58' N, 121º12' E. Taerqi, Yakeshi, Inner Mongolia Autonomous Region | *Betula platyphylla.* Rotten wood debris. Elevation 1000m. | G.F. Qin | PP442826 | PP444303 | PP443795 | PP356368 | PP443336 | PP443583 |
| *A. sinapina* | QZ23028_1 | 2023/9/3 | A | 47º58' N, 121º12' E. Taerqi, Yakeshi, Inner Mongolia Autonomous Region | *Betula platyphylla.* Dead stump. Elevation 1000m. | G.F. Qin | PP442828 | PP444305 | PP443797 | PP356370 | PP443338 | PP443585 |
| *A. sinensis* | QZ96026 | 1996/8/30 | F | 42º03' N, 128º01' E. Baihe, Changbai Mountain, Jilin Prov. | Rotten angiosperm wood debris. Elevation 700m. | G.F. Qin |  |  |  |  |  |  |
| *A. sinensis* | QZ96051 | 1996/9/10 | F | 41º19' N, 124º53' E. Laotuding, Huanren, Liaoning Prov. | *Quercus mongolica*. Rotten wood. Elevation 600m. | G.F. Qin, J. Zhao & S.M. Tian |  |  |  |  |  |  |
| *A. sinensis* | QZ96052 | 1996/9/11 | F | 41º19' N, 124º53' E. Laotuding, Huanren, Liaoning Prov. | *Pinus koraiensis*. Dead stump. Elevation 600m. | G.F. Qin, J. Zhao & S.M. Tian |  |  |  |  |  |  |
| *A. sinensis* | QZ96053 | 1996/9/11 | F | 41º19' N, 124º53' E. Laotuding, Huanren, Liaoning Prov. | *Fraxinus mandshurica*. Solitary at base of living tree. Elevation 600m. | G.F. Qin, J. Zhao & S.M. Tian |  |  |  |  |  |  |
| *A. sinensis* | QZ96054 | 1996/9/11 | F | 41º19' N, 124º53' E. Laotuding, Huanren, Liaoning Prov. | Caespitose on rotten wood. Elevation 700m. | G.F. Qin, J. Zhao & S.M. Tian |  |  |  |  |  |  |
| *A. sinensis* | QZ96055_14 | 1996/9/11 | F | 41º19' N, 124º53' E. Laotuding, Huanren, Liaoning Prov. | Caespitose on rotten wood. Elevation 800m. | G.F. Qin, J. Zhao & S.M. Tian | MN463902 | PP444329 | PP443813 | MN170678 | PP443121 | MN463763 |
| *A. sinensis* | QZ96056 | 1996/9/11 | F | 41º19' N, 124º53' E. Laotuding, Huanren, Liaoning Prov. | *Pinus koraiensis*. Solitary on base and root of dying tree. Elevation 900m. | G.F. Qin, J. Zhao & S.M. Tian |  |  |  |  |  |  |
| *A. sinensis* | QZ96058 | 1996/9/11 | F | 41º19' N, 124º53' E. Laotuding, Huanren, Liaoning Prov. | *Acer* sp. Dying tree. Elevation 1200m. | G.F. Qin, J. Zhao & S.M. Tian |  |  |  |  |  |  |
| *A. sinensis* | QZ96059_53 | 1996/9/11 | F | 41º19' N, 124º53' E. Laotuding, Huanren, Liaoning Prov. | *Fraxinus rhynchophylla*. Solitary to caespitose on dead stump. Elevation 700m. | G.F. Qin, J. Zhao & S.M. Tian | MN463903 | PP444331 | PP443815 | MN170603 | PP443127 | MN463765 |
| *A. sinensis* | QZ96060_21 | 1996/9/11 | F | 41º19' N, 124º53' E. Laotuding, Huanren, Liaoning Prov. | *Quercus mongolica*. Dead stump. Elevation 800m. | G.F. Qin, J. Zhao & S.M. Tian | PP442847 | PP444332 | PP443816 | MN170694 | MT674146 | MN463766 |
| *A. sinensis* | QZ97014 | 1997/9/9 | F | 42º12' N, 128º09' E. Huangsongpu, Changbai Mountain, Jilin Prov. | *Tilia* sp. Solitary on fallen trunk and dead stump. Elevation 1000m. | G.F. Qin & J. Zhao |  |  |  |  |  |  |
| *A. sinensis* | QZ97017 | 1997/9/9 | F | 42º12' N, 128º09' E. Huangsongpu, Changbai Mountain, Jilin Prov. | Angiospermous forest. Solitary or in large group on fallen trunk and wood debris. Elevation 1000m. | G.F. Qin & J. Zhao |  |  |  |  |  |  |
| *A. sinensis* | QZ97018 | 1997/9/9 | F | 42º12' N, 128º09' E. Huangsongpu, Changbai Mountain, Jilin Prov. | *Betula platyphylla*. Rotten stump. Elevation 1000m. | G.F. Qin & J. Zhao |  |  |  |  |  |  |
| *A. sinensis* | QZ97033_1 | 1997/9/11 | F | 42º 03' N, 128º01'E. Dianzhan houshan, Changbai Mountain, Jilin Prov. | *Acer* sp. At base of dead tree. Elevation 700m. | G.F. Qin & J. Zhao | MN463904 | PP444333 | MT674007 | MN170604 | MT674150 | MN463769 |
| *A. sinensis* | QZ97045_10 | 1997/9/14 | F | 42º12' N, 128º09' E. Huangsongpu, Changbai Mountain, Jilin Prov. | *Tilia amurensis*. Rotten stump. Elevation 1000m. | G.F. Qin & J. Zhao | MN463905 | PP444334 | PP443817 | MN170679 | PP443128 | MN463770 |
| *A. sinensis* | QZ99103_6 | 1999/9/3 | F | 47º01' N, 128º08' E. Yongcui, Dailing, Heilongjiang Prov. | *Larix* *gmelini*. Dead stump. Elevation 250m. | J. Zhao & S.M. Tian | MN463907 | PP444335 | MT674009 | MN170610 | MT674152 | MN463774 |
| *A. sinensis* | QZ99105 | 1999/9/3 | F | 47º01' N, 128º08' E. Yongcui, Dailing, Heilongjiang Prov. | *Pinus koraiensis*. Rotten stump. Elevation 250m. | J. Zhao & S.M. Tian | MN463908 |  | MT674010 | MT644845 | MT674153 | MN463775 |
| *A. sinensis* | QZ99107_9 | 1999/9/3 | F | 47º01' N, 128º08' E. Yongcui, Dailing, Heilongjiang Prov. | *Ulmus* sp. Solitary to caespitose on rotten stump. Elevation 250m. | J. Zhao & S.M. Tian | MN463909 | PP444336 | MT674011 | MN170606 | MT674154 | MN463776 |
| *A. sinensis* | QZ02160 | 2002/8/18 | F | 46º14' N, 133º05' E. Dongfanghong, Heilongjiang Prov. | *Betula* sp*.* Rotten wood debris. Elevation 100m. | G.T. Yang & L.F. Sun | PP442839 | PP444320 | PP443808 | MN170696 | PP443116 | PP443418 |
| *A. sinensis* | QZ05030_3 | 2005/9/18 | F | 34º00' N, 107º05' E. Honghegu, Taibai Mountain, Shaanxi Prov. | *Tillia* sp. Rotten fallen wood. Elevation 800m | G.F. Qin, J. Zhao & H.C. Wang | MN463915 | PP444321 | MT674003 | MN170608 | MT674140 | MN463781 |
| *A. sinensis* | QZ05047_8 | 2005/9/23 | F | 34º00' N, 107º05' E. Honghegu, Taibai Mountain, Shaanxi Prov. | Rotten angiosperm woods. Elevation 1200m. | J. Zhao & H.C. Wang | MN463916 | PP444323 | MT674004 | MN170609 | MT674141 | MN463782 |
| *A. sinensis* | W17021 | 2017/9/30 | F | 30º01' N, 110º03' E. Yanzi, Hefeng, Hubei Prov. | *Quercus* sp. Dead stump. Elevation 1542m. | Y. Wang, X. Xie, C. Mu | MN463917 |  | MT674012 | MN170686 | PP443129 | MN463783 |
| *A. sinensis* | QZ19020 | 2019/9/11 | F | 41º59' N, 124º30' E. Houtoushan, Qingyuan, Liaoning Prov. | Unkown | G.F. Qin | MT647113 | PP444324 | MT674005 | MT644841 | MT674142 | MT674258 |
| *A. sinensis* | QZ20003_1 | 2020/9/17 | F | 41º18' N, 124º10' E. Xiaoshi, Benxi, Liaoning Prov. | Unkown | G.F. Qin | PP442842 | PP444325 | PP443809 | PP356374 | PP443117 | PP443597 |
| *A. sinensis* | QZ20006_1 | 2020/9/22 | F | 41º37' N, 125º25' E. Gang Mountain, Qingyuan, Liaoning Prov. | *Larix olgensis.* Base of living tree. Elevation 1000m. | G.F. Qin | PP442843 | PP444326 | PP443810 | PP356375 | PP443118 | PP443598 |
| *A. sinensis* | QZ21016_1 | 2021/9/8 | F | 42º03' N, 128º03' E. Yuehualin, Changbai Mountain, Jilin Prov. | *Betula ermanii.* Elevation 1850m. | G.F. Qin | PP442844 | PP444327 | PP443811 | PP356376 | PP443119 | PP443419 |
| *A. sinensis* | QZ21049_3 | 2021/9/30 | F | 41º51' N, 123º24' E. Shenyang, Liaoning Prov. | *Ulmus* sp. Base of dead planted tree. Elevation 300m. | G.F. Qin | PP442845 | PP444328 | PP443812 | PP356377 | PP443120 | PP443599 |
| *A. tibetica* | QZ00101 | 2000/9/14 | J | 27º06' N, 100º12' E. Yulong Snow Mountain, Yunnan Prov. | *Betula* sp. Rotten wood. Elevation 3500m. | J. Zhao & S.M. Tian |  |  |  |  |  |  |
| *A. tibetica* | QZ00102 | 2000/9/14 | J | 27º05' N, 100º15' E. Dasuodao, Yulong Snow Mountain, Yunnan Prov. | *Betula* sp. Rotten wood. Elevation 3500m. | J. Zhao & S.M. Tian |  |  |  |  |  |  |
| *A. tibetica* | QZ00103 | 2000/9/14 | J | 27º05' N, 100º15' E. Dasuodao, Yulong Snow Mountain, Yunnan Prov. | *Picea likiangensis*. Rotten fallen wood. Elevation 3500m. | J. Zhao & S.M. Tian |  |  |  |  |  |  |
| *A. tibetica* | QZ00104 | 2000/9/14 | J | 27º05' N, 100º15' E. Dasuodao, Yulong Snow Mountain, Yunnan Prov. | *Betula* sp. Rotten wood. Elevation 3356m. | J. Zhao & S.M. Tian |  |  |  |  |  |  |
| *A. tibetica* | QZ00105 | 2000/9/14 | J | 27º05' N, 100º15' E. Dasuodao, Yulong Snow Mountain, Yunnan Prov. | Angiospermous forest. Rotten wood debris. Elevation 3356m. | J. Zhao & S.M. Tian |  |  |  |  |  |  |
| *A. tibetica* | QZ00106 | 2000/9/14 | J | 27º05' N, 100º15' E. Dasuodao, Yulong Snow Mountain, Yunnan Prov. | Angiospermous forest. Rotten wood debris. Elevation 3356m. | J. Zhao & S.M. Tian |  |  |  |  |  |  |
| *A. tibetica* | QZ00107 | 2000/9/14 | J | 27º05' N, 100º15' E. Dasuodao, Yulong Snow Mountain, Yunnan Prov. | Angiospermous forest. Rotten wood debris. Elevation 3356m. | J. Zhao & S.M. Tian |  |  |  |  |  |  |
| *A. tibetica* | QZ00108 | 2000/9/14 | J | 27º05' N, 100º15' E. Dasuodao, Yulong Snow Mountain, Yunnan Prov. | *Picea likiangensis*. Rotten wood. Elevation 3356m. | J. Zhao & S.M. Tian |  |  |  |  |  |  |
| *A. tibetica* | QZ00110 | 2000/9/14 | J | 27º06' N, 100º12' E. Yunshanping, Yulong Snow Mountain, Yunnan Prov. | Angiospermous forest. Rotten wood. Elevation 3240m. | J. Zhao & S.M. Tian |  |  |  |  |  |  |
| *A. tibetica* | QZ00112 | 2000/9/14 | J | 27º06' N, 100º12' E. Yunshanping, Yulong Snow Mountain, Yunnan Prov. | Angiospermous forest. Rotten wood. Elevation 3240m. | J. Zhao & S.M. Tian |  |  |  |  |  |  |
| *A. tibetica* | QZ00115 | 2000/9/17 | J | 27º48' N, 99º54' E. Shuodu lake, Shangrila, Yunnan Prov. | *Abies ferreana*. Solitary on rotten wood. Elevation 3700m. | J. Zhao & S.M. Tian |  |  |  |  |  |  |
| *A. tibetica* | QZ00116 | 2000/9/17 | J | 27º48' N, 99º54' E. Shuodu lake, Shangrila, Yunnan Prov. | Rotten wood debris. Elevation 3700m. | J. Zhao & S.M. Tian |  |  |  |  |  |  |
| *A. tibetica* | QZ00117 | 2000/9/17 | J | 27º48' N, 99º54' E. Shuodu lake, Shangrila, Yunnan Prov. | *Abies ferreana*. Solitary on rotten wood. Elevation 3700m | J. Zhao & S.M. Tian |  |  |  |  |  |  |
| *A. tibetica* | QZ00118 | 2000/9/17 | J | 27º48' N, 99º54' E. Shuodu lake, Shangrila, Yunnan Prov. | *Abies ferreana*. Rotten wood. Elevation 3700m | J. Zhao & S.M. Tian |  |  |  |  |  |  |
| *A. tibetica* | QZ00119 | 2000/9/17 | J | 27º48' N, 99º54' E. Shuodu lake, Shangrila, Yunnan Prov. | *Abies ferreana*. Solitary to caespitose on dying tree. Elevation 3700m. | J. Zhao & S.M. Tian |  |  |  |  |  |  |
| *A. tibetica* | QZ00121 | 2000/9/17 | J | 27º48' N, 99º54' E. Shuodu lake, Shangrila, Yunnan Prov. | *Abies ferreana*. Solitary to caespitose on rotten wood. Elevation 3700m. | J. Zhao & S.M. Tian |  |  |  |  |  |  |
| *A. tibetica* | QZ00122 | 2000/9/17 | J | 27º43' N, 99º58' E. Xilianpen yakou, Shangrila, Yunnan Prov. | *Quercus pannosa*. Solitary to caespitose on dying tree. Elevation 3500m. | J. Zhao & S.M. Tian |  |  |  |  |  |  |
| *A. tibetica* | QZ02028_1 | 2002/9/20 | J | 44º50' N, 81º02' E. Guozigou, Xinyuan, Xinjiang Autonomous Region | *Picea schrenkiana.* Elevation 2000m. | J. Zhao | PP480582 | PP444370 | PP443849 | PP356327 | PP443131 | PP443638 |
| *A. tibetica* | QZ02029_1 | 2002/9/20 | J | 44º50' N, 81º02' E. Guozigou, Xinyuan, Xinjiang Autonomous Region | *Picea schrenkiana.* Elevation 2000m. | J. Zhao | PP480583 | PP444371 | PP443850 | PP356328 | PP443132 | PP443639 |
| *A. tibetica* | QZ02030_5 | 2002/9/20 | J | 44º50' N, 81º02' E. Guozigou, Xinyuan, Xinjiang Autonomous Region | *Picea schrenkiana.* Elevation 2000m. | J. Zhao | PP480584 | PP444372 | PP443851 | PP356329 | PP443133 | PP443640 |
| *A. tibetica* | QZ02044_6 | 2002/9/23 | J | 43º17' N, 84º01' E. Nalati Yangshugou, Xinyuan County, Xinjiang Autonomous Region | Cutting-blank of *Betula pendula*. Solitary to caespitose on dead stump. Elevation 1350m. | J. Zhao | MN463947 | PP444373 | MT673989 | MN170641 | MT674128 | MN463809 |
| *A. tibetica* | QZ02045 | 2002/9/23 | J | 43º17' N, 84º01' E. Nalati, Xinyuan, Xinjiang Autonomous Region | *Betula pensula*. Dead stump. Elevation 1350m. | J. Zhao |  |  |  |  |  |  |
| *A. tibetica* | QZ02046 | 2002/9/23 | J | 43º17' N, 84º01' E. Nalati, Xinyuan, Xinjiang Autonomous Region | *Betula pensula*. Dead stump. Elevation 1350m. | J. Zhao |  |  |  |  |  |  |
| *A. tibetica* | QZ02047_2 | 2002/9/23 | J | 43º03'N, 84º01'E. Nalati, Xinyuan, Xinjiang Autonomous Region | *Betula pensula*. Dead stump. Elevation 1350m. | J. Zhao | MN463948 | PP444374 | PP443852 | MN170684 | PP443134 | MN463810 |
| *A. tibetica* | QZ02048 | 2002/9/23 | J | 43º17' N, 84º01' E. Nalati, Xinyuan County, Xinjiang Autonomous Region | *Betula pensula*. Dead stump. Elevation 1350m. | J. Zhao |  |  |  |  |  |  |
| *A. tibetica* | QZ02049 | 2002/9/23 | J | 43º17' N, 84º01' E. Nalati, Xinyuan, Xinjiang Autonomous Region | *Picea schrenkiana*. Dead stump. Elevation 1350m. | J. Zhao | MT647105 |  |  | MT644829/PP356330 | MT674129 | MT674249 |
| *A. tibetica* | QZ02050 | 2002/9/23 | J | 43º17' N, 84º01' E. Nalati, Xinyuan, Xinjiang Autonomous Region | *Picea schrenkiana*. Dead wood. Elevation 1350m. | J. Zhao |  |  |  |  |  |  |
| *A. tibetica* | QZ02051_5 | 2002/9/23 | J | 43º17' N, 84º01' E. Nalati, Xinyuan, Xinjiang Autonomous Region | *Picea schrenkiana*. Rotten wood. Elevation 1350m. | J. Zhao | MN463949 | PP444375 | MT673990 | MN170685 | PP443135 | MN463811/MT674250 |
|  |  |  |  |  |  |  |  |  |  |  |  |  |
| *A. tibetica* | QZ04001 | 2004/7/30 | J | 29º38' N, 94º22' E. Bayi, Nyingchi, Xizang Autonomous Region | *Picea* sp. Rotten wood. Elevation 3000m. | Y.C. Dai |  |  |  |  |  |  |
| *A. tibetica* | QZ04002 | 2004/7/30 | J | 29º38' N, 94º22' E. Bayi, Nyingchi, Xizang Autonomous Region | *Picea* sp. Rotten wood. Elevation 3000m. | Y.C. Dai |  |  |  |  |  |  |
| *A. tibetica* | QZ04008 | 2004/7/31 | J | 29º59' N, 93º52' E. Cuogao, Gongbujiangda, Nyingchi, Xizang Autonomous Region | *Abies* sp. Rotten trunk. Elevation 3000m. | Y.C. Dai |  |  |  |  |  |  |
| *A. tibetica* | QZ04017 | 2004/8/7 | J | 34º07' N, 107º53' E. Taibai Mountain, Mei, Shaanxi Prov. | *Betula albo-sinensis*. Trunk of living tree. Elevation 2800m. | Y.C. Dai |  |  |  |  |  |  |
| *A. tibetica* | QZ04018 | 2004/8/7 | J | 34º07' N, 107º53' E. Taibai Mountain, Mei, Shaanxi Prov. | *Betula albo-sinensis*. Base of dying tree. Elevation 2800m. | Y.C. Dai |  |  |  |  |  |  |
| *A. tibetica* | QZ04032 | 2004/7/30 | J | 29º59' N, 93º52' E. Cuogao, Gongbujiangda, Nyingchi, Xizang Autonomous Region | *Abies* sp. Dead stump. Elevation 3800m. | C.J. Yu & H.C. Wang |  |  |  |  |  |  |
| *A. tibetica* | QZ06003 | 2006/9/23 | J | 37º52' N, 111º28' E. Pangquangou, Hengjian, Taiyuan, Shanxi Prov. | *Picea* sp. Rotten wood. Elevation 1300m. | H.C. Wang |  |  |  |  |  |  |
| *A. tibetica* | QZ19076 | 2019/10/16 | J | 29º32' N, 103º19' E. Jieyindian, Emei Mountain, Sichuan Prov. | Solitary or caespitose at base of living *Acer* sp. Elevation 2400m. | G.F. Qin |  |  |  |  |  |  |
| *A. tibetica* | QZ19077 | 2019/10/16 | J | 29º32' N, 103º19' E. Leidongping, Emei Mountain, Sichuan Prov. | Solitary on dead trunk of *Betula* sp. Elevation 2350m | G.F. Qin |  |  |  |  |  |  |
| *A. tibetica* | QZ19096 | 2019/10/21 | J | 30º08'N. 110º 57' E. Yangzixi, Houhe Nature Reserve, Wufeng, Hubei Prov. | Ground of mixed forest. Elevation 1700m. | G.F. Qin |  |  |  |  |  |  |
| *A. tibetica* | QZ19097 | 2019/10/21 | J | 30º08'N. 110º 57' E. Yangzixi, Houhe Nature Reserve, Wufeng, Hubei Prov. | Living trees of *Cerasus pseudocerasus*. Elevation 1700m. | G.F. Qin |  |  |  |  |  |  |
| *A. tibetica* | NLT1_2 | 2021/9/7 | J | 43º18' N, 84º32' E. Gongnaisi, Hejing, Xinjiang Autonomous Region | Elevation 2612m. | J.W. Liu | PP480575 | PP444363 | PP443842 | PP356320 | PP443130 | PP443631 |
| *A. tibetica* | NLT2_2 | 2021/9/8 | J | 43º19' N, 84º17' E. Xinyuan, Xinjiang Autonomous Region | Elevation 1741m. | J.W. Liu | PP480576 | PP444364 | PP443843 | PP356321 | PP443136 | PP443632 |
| *A. tibetica* | NLT6A_4 | 2021/9/13 | J | 43º18' N, 84º17' E. Nalati, Xinyuan, Xinjiang Autonomous Region | Elevation 1850m. | J.W. Liu | PP480577 | PP444365 | PP443844 | PP356322 | PP443137 | PP443633 |
| *A. tibetica* | NLT6B_3 | 2021/9/13 | J | 43º18' N, 84º17' E. Nalati, Xinyuan, Xinjiang Autonomous Region | Elevation 1850m. | J.W. Liu | PP480578 | PP444366 | PP443845 | PP356323 | PP443138 | PP443634 |
| *A. tibetica* | NLT6C_5 | 2021/9/13 | J | 43º18' N, 84º17' E. Nalati, Xinyuan, Xinjiang Autonomous Region | Elevation 1850m. | J.W. Liu | PP480579 | PP444367 | PP443846 | PP356324 | PP443142 | PP443635 |
| *A. tibetica* | NLT6D_4 | 2021/9/13 | J | 43º18' N, 84º17' E. Nalati, Xinyuan, Xinjiang Autonomous Region | Elevation 1850m. | J.W. Liu | PP480580 | PP444368 | PP443847 | PP356325 | PP443143 | PP443636 |
| *A. tibetica* | QZ21046_3 | 2021/9/24 | J | 42º55' N, 84º47' E. Kesang Forest Park, Tex, Xinjiang Autonomous Region | *Picea* sp. Dead stump. Elevation 1800m. | B.K. Cui | PP480597 | PP444398 | PP443869 | PP356348 | PP443139 | PP443654 |
| *A. tibetica* | QZ21047_1 | 2021/9/24 | J | 42º55' N, 84º47' E. Kesang Forest Park, Tex, Xinjiang Autonomous Region | *Picea* sp. Dead stump. Elevation 1800m. | B.K. Cui | PP480598 |  | PP443870 | PP356349 | PP443140 | PP443655 |
| *A. tibetica* | QZ21048_1 | 2021/9/24 | J | 42º55' N, 84º47' E. Kesang Forest Park, Tex, Xinjiang Autonomous Region | *Picea* sp. Dead stump. Elevation 1800m. | B.K. Cui | PP480599 | PP444399 | PP443871 | PP356350 | PP443141 | PP443656 |
| *A. violacea* | QZ02065 | 2002/10/19 | N | 29º34' N, 103º21' E. Emei Mountain, Sichuan Prov. | *Castanopsis platyacantha*. Rotten wood. Elevation 1400m. | Y.C. Dai |  |  |  |  |  |  |
| *A. violacea* | QZ02066 | 2002/10/19 | N | 29º34' N, 103º21' E. Emei Mountain, Sichuan Prov. | *Castanopsis platyacantha*. Rotten wood. Elevation 1400m. | Y.C. Dai |  |  |  |  |  |  |
| *A. violacea* | QZ02067 ^_2^ | 2002/10/19 | N | 29º34' N, 103º21' E. Emei Mountain, Sichuan Prov. | *Castanopsis platyacantha*. Rotten wood. Elevation 1400m. | Y.C. Dai | MN463985 | PP444410 | MT674015 | MN170663 | MT674159 | MN463849 |
| *A. violacea* | QZ02068 | 2002/10/19 | N | 29º34' N, 103º21' E. Emei Mountain, Sichuan Prov. | *Castanopsis platyacantha*. Rotten wood. Elevation 1400m. | Y.C. Dai | MT647115 | PP444411/  PP444412 | MT674016 | MT644846 | MT674160 | MN463850 |
| *A. violacea* | QZ02069_8 | 2002/10/19 | N | 29º34' N, 103º21' E. Emei Mountain, Sichuan Prov. | *Castanopsis platyacantha*. Rotten wood. Elevation 1400m. | Y.C. Dai | MT647116 | PP444413 | MT674017 | MT644847 | MT674161 | MT674259 |
| *A. violacea* | QZ02070 | 2002/10/19 | N | 29º34' N, 103º21' E. Emei Mountain, Sichuan Prov. | *Castanopsis platyacantha*. Rotten wood. Elevation 1400m. | Y.C. Dai | MT647117 |  | MT674018 | MT644848 | MT674162 | MT647117 |
| *A. violacea* | QZ05014 | 2005/9/17 | N | 34º00' N, 107º50' E. Shiwaitaoyuan, Taibai Mountain, Shannxi Prov. | *Betula* sp. Living tree. Elevation 1600m. | G.F. Qin, J. Zhao & H.C. Wang |  |  |  |  |  |  |
| *A. violacea* | QZ05015 | 2005/9/17 | N | 34º00' N, 107º50' E. Shiwaitaoyuan, Taibai Mountain, Shannxi Prov. | *Magnolia liliflora*. Dead trunk. Elevation 1500m. | G.F. Qin, J. Zhao & H.C. Wang |  |  |  |  |  |  |
| *A. violacea* | QZ05017_1 | 2005/9/17 | N | 34º00' N, 107º50' E. Shiwaitaoyuan, Taibai Mountain, Shannxi Prov. | Living angiosperm roots. Elevation 1500m. | G.F. Qin, J. Zhao & H.C. Wang | PP442779 | PP444414 | PP443880 | PP356383 | PP443387 | PP442779 |
| *A. violacea* | W16024 | 2016/10/3 | N | 31º40' N, 110º25' E. Shennongjia, Hubei Prov. | *Quercus* sp. Dead stump. Elevation 2146m. | C.H. Zeng & M. Wang | MN463991 | PP444423 | MT674030 | MN170687 | MT674169 | MN463991 |
| *A. violacea* | QZ19098_7 | 2019/10/21 | N | 30º04' N, 110º32' E. Yangzixi, Houhe Nature Reserve, Wufeng, Hubei Prov. | Solitary on fallen trunk of *Cunninghamia lanceolata*. Elevation 1700m. | G.F. Qin | MT647119 |  | MT674020 | MT644849 |  | MT647119 |
| *A. violacea* | QZ19099 | 2019/10/21 | N | 30º04' N, 110º32' E. Yangzixi, Houhe Nature Reserve, Wufeng, Hubei Prov. | Solitary on roots of *Cunninghamia lanceolata*. Elevation 1700m. | G.F. Qin | MT647120 | PP444415 | MT674021 | MT644850 | MT674163 | MT647120 |
| *A. violacea* | QZ19107 | 2019/10/29 | N | 29º34' N, 103º37' E. Zhanglaoping, Emei Mountain, Sichuan Prov. | Caespitose on rotten angiosperm stump. Elevation 1700m. | G.F. Qin | MT647121 |  | MT674022 | MT644851 | MT674164 | MT647121 |
| *A. violacea* | QZ19108 | 2019/10/29 | N | 29º34' N, 103º21' E. Zhanglaoping, Emei Mountain, Sichuan Prov. | Caespitose on fallen angiosperm trunk. Elevation 1700m. | G.F. Qin | MT647122 | PP444416 | MT674023 | MT644852 | PP443388 | MT647122 |
| *A. violacea* | QZ19109 | 2019/10/29 | N | 29º33' N, 103º21' E. Chudianxia, Emei Mountain, Sichuan Prov. | Caespitose on dead angiosperm stand trunk. Elevation 1800m. | G.F Qin | MT647123 |  | MT674024 | MT644853 | MT674165 | MT647123 |
| *A. violacea* | QZ19110_4 | 2019/10/29 | N | 29º33' N, 103º21' E. Chudianxia, Emei Mountain, Sichuan Prov. | Caespitose on fallen angiosperm trunk. Elevation 1800m. | G.F. Qin | MT647124 | PP444417 | MT674025 | MT644854 | MT674166 | MT647124 |
| *A. violacea* | QZ19122 | 2019/10/31 | N | 30º04' N, 110º32' E. Yangzixi, Houhe Nature Reserve, Wufeng, Hubei Prov. | Solitary or caespitose on dead angiosperm trunk. Elevation 1700m. | G.F. Qin | MT647125 |  | MT674026 | MT644855 | MT674167 | MT647125 |
| *A. violacea* | QZ19123_3 | 2019/10/31 | N | 30º04' N, 110º32' E. Yangzixi, Houhe Nature Reserve, Wufeng, Hubei Prov. | Solitary or caespitose on dead trunk of *Quercus* sp. Elevation 1700m. | G.F. Qin | PP442780 | PP444418 | MT674027 | MT644856 | PP443389 | PP442780 |
| *A. violacea* | QZ19127_5 | 2019/10/31 | N | 30º04' N, 110º32' E. Yangzixi, Houhe Nature Reserve, Wufeng, Hubei Prov. | Angiosperms. Elevation 1800m. | G.F. Qin | MT647126 | PP444419 | MT674028 | MT644857 | PP443390 | MT647126 |
| *A. violacea* | QZ19132_4 | 2019/11/5 | N | 30º04' N, 110º32' E. Yangzixi, Houhe Nature Reserve, Wufeng, Hubei Prov. | Cluster at base of living angiosperm. Elevation 1700m. | G.F. Qin | MT647127 | PP444420 | MT674029 | MT644858 | MT674168 | MT647127 |
| *A. violacea* | 21SNJ11_1 | 2021/9/27 | N | 31º29' N, 110º25' E. Muyu, Shennongjia, Hubei Prov. | Angiosperms. Elevation 1689m. | J.W. Liu |  | PP444408 | PP443878 | PP356381 | PP443385 | PP442685 |
| *A. violacea* | 21SNJ25_2 | 2021/9/27 | N | 31º3' N, 110º2' E. Muyu, Shennongjia, Hubei Prov. | Angiosperms. Elevation 1923m. | J.W. Liu | PP442777 | PP444409 | PP443879 | PP356382 | PP443386 | PP442777 |
| *A. violacea* | QZ21057_4 | 2021/10/24 | N | 29º47' N, 95º41' E. Cangkong, Bomi, Xizang Autonomous Region | *Abies* sp. Elevation 3670m. | Y.C. Dai | PP442781 | PP444421 | PP443881 | PP356384 | PP443391 | PP442781 |
| *A. violacea* | QZ21058_2 | 2021/10/24 | N | 29º47' N, 95º41' E. Cangkong, Bomi, Xizang Autonomous Region | *Abies* sp. Elevation 3670m. | Y.C. Dai | PP442782 | PP444422 | PP443863 | PP356385 | PP443392 | PP442782 |
| *D. ectypa* | KK70011_13 |  |  | France |  | J.J. Guillaumin | PP442783 | PP444424 | PP444005 | PP356387 | PP443393 | PP442783 |
| *^*^D. ectypa* | FPL83.16 |  |  | Genome sequence. CFMR, USA |  |  | Armect1scaffold_4:552839-553593 | Armect1scaffold_4:2876593-2877893 | Armect1scaffold_4:2867873-2869338 | Armect1scaffold_30:3496-4495 | Armect1scaffold_3:197085-198344 | Armect1scaffold_3:1327039-1327603 |
| *D. tabescens* | QZ99122_1 | 1999/9/9 | I | 39º56' N, 116º19' E. Beijing Zoon. | Base of dying tree *of Salix bobylonica*. Dead stump of *Amygdalus triloba*. Elevation 40m. | G.F. Qin | MN463940 | PP444435 | MT674014 | MN170635 | MT674157 | MN463940 |
| *D. tabescens* | Dai 18153 | 2017/9/17 |  | 25º02' N, 113º45' E. Danxiashan Nature Reserve, Renhua, Guangdong Prov. | Rotten angiosperm wood | Y.C. Dai | MN463944 |  | MT674013 | MN170636 |  | MN463944 |
| *D. tabescens* | ACCC51415 |  |  | Sichuan Prov. | Unknown |  | PP442632 | PP444425 | PP443992 | PP356388 | PP443394 | PP442632 |
| *D. tabescens* | CS1095_53 |  |  | Italy, Cosenza | Unknown | R. Grillo | PP442633 | PP444428 | PP443993 |  | PP443395 | PP443673 |
| *D. tabescens* | LDM00018_3 | 2022/6/22 | I | Kunming, Yunnan Prov. | Unknown | J.W. Liu | PP442637 | PP444431 | PP443996 | PP356392 | PP443398 | PP443681 |
| *D. tabescens* | LJW2993_1 | 2022/6/22 | I | Kunming, Yunnan Prov. | Unknown | J.W. Liu | PP442638 | PP444432 | PP443997 | PP356393 | PP443399 | PP443682 |

*All the sequences listed in the Table S1 are new sequences except for the sequences of *D. ectypa* FPL83.16 from genome.
